# Interactions Between Enrichment Planted Seedlings and Naturally Occurring Trees in Selectively Logged Lowland Dipterocarp Forest

**DOI:** 10.1002/ece3.73439

**Published:** 2026-05-03

**Authors:** Charles J. Marsh, Ryan Veryard, Martin Svátek, Elena Fernandez‐Miranda Cagigal, Elia Godoong, Jakub Kvasnica, Radim Matula, Michael J. O'Brien, Martin Rejžek, Edgar C. Turner, Andy Hector

**Affiliations:** ^1^ Department of Biology University of Oxford Oxford UK; ^2^ Department of Biological Sciences and Centre for Nature‐Based Climate Solutions National University of Singapore Singapore Singapore; ^3^ Faculty of Forestry and Wood Technology Mendel University in Brno Brno Czech Republic; ^4^ Dendra Systems Unit A, Oakfield Industrial Estate Witney UK; ^5^ Faculty of Tropical Forestry Universiti Malaysia Sabah, Jalan UMS Kota Kinabalu Sabah Malaysia; ^6^ Department of Forest Ecology, Faculty of Forestry and Wood Sciences Czech University of Life Sciences Prague Prague Czech Republic; ^7^ Estación Experimental de Zonas Áridas Consejo Superior de Investigaciones Científicas, Carretera de Sacramento s/n Almería Spain; ^8^ The South East Asia Rainforest Research Partnership (SEARRP) Danum Valley Field Centre Lahad Datu Sabah Malaysia; ^9^ Department of Zoology University of Cambridge Cambridge UK; ^10^ Department of Biology and Leverhulme Centre for Nature Recovery University of Oxford Oxford UK

**Keywords:** basal area, canopy openness, Dipterocarpaceae, enrichment planting, selective logging, survival

## Abstract

Old‐growth forests in Southeast Asia are dominated by trees of the Dipterocarpaceae family which are targeted by selective logging. Their traits (supra‐annual mast fruiting, limited dispersal, and recalcitrant seeds that form no seed bank) mean they can have poor natural regeneration rates in some selectively logged forests. Enrichment planting is used to attempt to overcome this recruitment limitation and increase restoration success. However, it is still unclear what factors influence the performance of planted seedlings. Here, we analyse the growth and survival between 2012 and 2015 of 721 enrichment line‐planted seedlings from 16 species of dipterocarps within the selectively logged forest of the Sabah Biodiversity Experiment, alongside the location, size and identity of nearly 5000 naturally occurring trees within 10 m of focal planted seedlings. We analysed the survival and growth of enrichment planted dipterocarp seedlings in relation to three properties of the surrounding naturally occurring vegetation: (1) canopy openness; (2) the abundance of naturally occurring dipterocarps (proportion of total basal area); (3) the presence of nearby (< 10 m) large trees (basal area of the largest tree as a proportion of the total). Survival and growth rates of enrichment planted seedlings were positively associated with canopy openness and total basal area of surrounding trees. These results were consistent between the two planting cohorts. Increased survival and growth of enrichment planted seedlings in areas of Sabah Biodiversity Experiment with higher canopy openness (up to around 40%) is consistent with understory light as a limiting resource. The unexpected higher survival and growth of enrichment planted seedlings in forest areas with higher basal area of unlogged trees may be explained by the creation of patches that are better or worse for tree growth, with areas less heavily impacted by logging containing both more naturally occurring trees and providing better conditions for the survival and growth of planted seedlings.

## Graphical abstract

This study analyzed the growth and survival of 721 seedlings in Malaysian Borneo in relation to their local neighbourhood of surrounding matrix trees. We found that both were positively influenced by canopy openness and the basal area of surrounding trees, highlighting the importance of light availability and the benefits of areas with more unlogged trees in supporting seedling development.

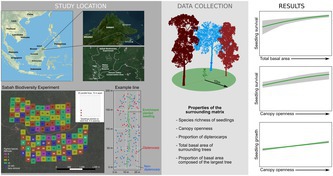

## 1 Introduction

The tropical lowland forests of Southeast Asia are dominated by trees of the Dipterocarpaceae, which make up over 50% of forest basal area and 60% of standing volume (Sist and Saridan 1999). These regions are regarded as carbon hotspots with high stocks in tree biomass. For instance, undisturbed lowland forests of Borneo exhibit aboveground biomass values approximately 60% greater than the Amazon (457.1 Mg ha^−1^ vs. 288.6 Mg ha^−1^; Slik et al. 2010). Dipterocarp physical traits, specifically hardwood durability, straight and tall trunks, and attractive appearance of timber, have resulted in the extensive extraction of mature dipterocarps across Southeast Asia (Estoque et al. 2019). Selective logging has been shown to remove between 55% and 66% of dipterocarp stock (individuals above 10 cm diameter at breast height (DBH); Saner et al. 2012), and this basal area is often initially replaced by fast‐growing pioneer species that contribute less to a forest's carbon stock (Saner et al. 2012).

The life history traits of many dipterocarps may hinder recovery following disturbance. Rather than fruiting annually, these trees undergo irregular ‘mast fruiting’ events every 2–10 years (Ashton et al. 1988). Dipterocarp seeds are often relatively large and have limited dispersal (despite usually being winged) and are also recalcitrant, forming a seedling bank instead of a soil seed bank (Umarani et al. 2015). Under ideal conditions, seeds germinate immediately but are known to suffer high mortality in open, dry, inundated, or otherwise disturbed areas (Appanah and Turnbull 1998). Logging can cause extensive damage to seedling banks directly but may also indirectly harm seedling survival by reducing soil quality through compaction (Hattori et al. 2013; Nussbaum et al. 1995). Disturbance from logging can also increase cover of ferns, vines and lianas suppressing succession (O'Brien et al. 2019). The decline of aboveground biomass and changes in tree communities via disturbance can also negatively impact soil physiochemical composition, particularly in terms of soil carbon, nitrogen, and phosphorous (Ngaba et al. 2020; Xu et al. 2018). Taken together, these characteristics may limit the ability of dipterocarps to recover following selective logging.

Regeneration of this timber is environmentally and economically important for countries with these resources. One associated technique aimed at accelerating recovery is the enrichment planting of the existing seedling bank with additional nursery‐grown stock. This is commonly done by planting along cleared lines through a forest within the pre‐existing matrix of seedlings and mature trees left after logging. It is an expensive process, costing between $1500 and $2500 ha^−1^ in lowland Sabah (Philipson et al. 2020) so it is important that this restoration method provides tangible benefits.

The effectiveness of such initiatives is based on the survival and growth of planted seedlings until they reach maturity. Therefore, predicting the outcome of enrichment planting is important for communities that depend on these forest products. Recent studies have investigated the response of dipterocarp seedling survival and growth to factors including water availability and frequency of rainfall (O'Brien et al. 2013, 2015), light availability (Philipson et al. 2012), and ectomycorrhizal associations (Saner et al. 2011). Studies have also investigated more applied factors, such as the diversity of species used in enrichment planting (Tuck et al. 2016) and the effect of liana removal (O'Brien et al. 2019). However, many studies have been conducted in easily controllable environments like shade houses (e.g., Ashton et al. 2006), which make experimental manipulations more feasible but exclude broader effects of the natural environment. Therefore, factors impacting seedling survival and growth need to be better understood within the context of the ecosystem where seedlings are planted.

In logged forests with enrichment planting, a mixture of factors describe the matrix of vegetation surrounding planted seedlings, including the number of surrounding trees, the size of these trees, and their taxonomic identity. These factors are partly a consequence of the logging history of the forest. In relatively undisturbed areas, we might expect fewer larger trees while in areas of intensive selective logging, we might expect a greater number of small trees, mostly pioneer species undergoing competitive release. We would expect a higher proportion of non‐dipterocarps due to the selective removal of commercially valuable dipterocarp species during logging and subsequent colonisation of pioneer non‐dipterocarp species. Although some non‐dipterocarp species are highly valued and have experienced extraction in dipterocarp‐dominated forests (Hayward et al. 2021), most timber harvesting within dipterocarp forests is driven by the extraction of the Dipterocarpaceae. The survival and growth of planted seedlings is likely to be influenced by the properties of the surrounding matrix vegetatation. Generally, increasing the surrounding basal area results in greater local competition and reduced seedling growth and survival (Kobe and Vriesendorp 2011; Peters 2003). Larger trees can compete strongly and asymmetrically with seedlings, often for light (Potvin and Dutilleul 2009; Velázquez and Wiegand 2020). Competition for resources may act alongside indirect effects (Bachelot et al. 2020): higher conspecific density can increase mortality levels, as species‐specific pests and pathogens can more easily find appropriate hosts (Connell 1971; Janzen 1970; Oshima et al. 2015). Seedling mortality can be most substantial closer to (within 10 m) adult trees (Murphy et al. 2017).

In contrast, positive density‐dependant effects can arise through facilitation, where larger trees create more suitable microclimates (Bruno et al. 2003). For instance, an established canopy can reduce extreme variations in temperature and humidity, reducing the vapour‐pressure deficit seedlings experience and potentially resulting in reduced water stress and drought‐related mortality in seedlings (Adams et al. 2017). Young, recently planted seedlings are expected to benefit most from such canopy‐related water stress mitigation, as they are smaller and less resistant to environmental perturbations (Turner 2001). An increased proportion of confamilial individuals could also reduce local mortality through predator satiation (Iku et al. 2017), especially given that up to 92% of dipterocarp species have been observed masting fruit simultaneously (Curran and Leighton 2000).

This study used a long‐term, field scale study (Sabah Biodiversity Experiment—see Methods) to investigate how the survival and growth of enrichment planted seedlings from 16 dipterocarp species were affected by properties of the surrounding naturally occurring vegetation. We mapped the size, and identity (dipterocarp or non‐dipterocarp) of naturally occurring trees (≥ 10 cm DBH) within the selectively logged vegetation between enrichment planting lines. We then used linear mixed‐effects models (and generalised versions) to estimate the effects on the survival and growth of enrichment planted seedling of the influence of canopy openness; the total impacts of all nearby trees (total basal area of all trees within 10 m); the impacts of nearby dipterocarps (i.e., confamilial trees only); and the effects of nearby large trees (the proportion of basal area belonging to the largest tree: ‘proportion largest individual’).

## 2 Materials and Methods

### 2.1 Study Site

**FIGURE 1:**
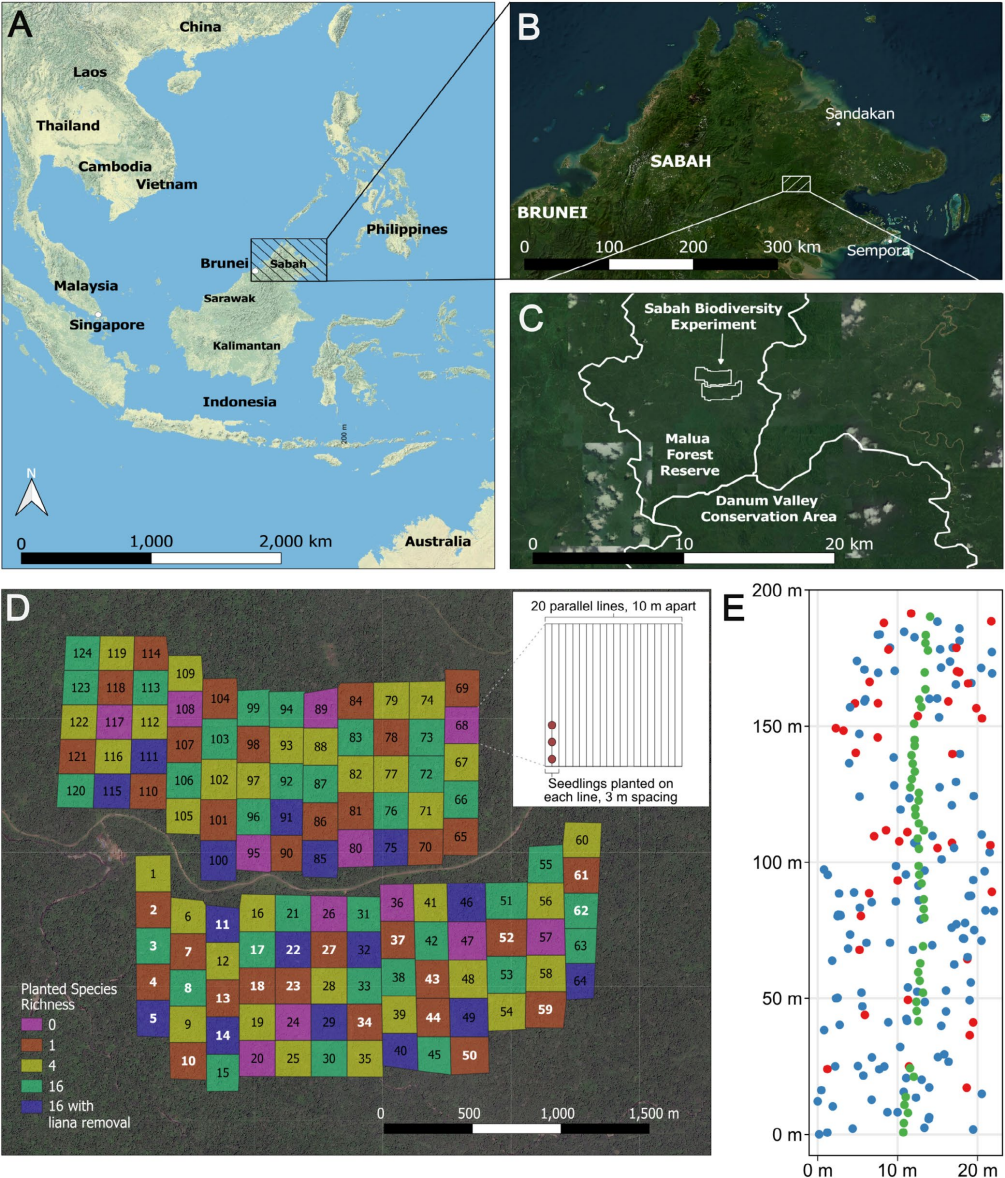
Location and design of the Sabah Biodiversity Experiment. (A) The Sabah Biodiversity Experiment is located within Sabah, Malaysian Borneo. (B, C) The experimental site exists within the Malua Forest Reserve, just north of the Danum Valley Conservation Area and approximately 70 km west of the town of Lahad Datu. (D) The Sabah Biodiversity Experiment consists of 124 four‐hectare plots split into two blocks separated by a logging road. Each plot has one of five possible treatments applied—unplanted controls (*0*), sites planted with either one, four, or 16 species (*1*, *4*, and *16*, respectively), and 16‐species plots which have also undergone an additional treatment of liana removal (*16 with liana removal*). In each enrichment planted plot, dipterocarp seedlings were planted every three m along 2 parallel lines 10 m apart. Plot treatment is indicated in the legend by colour. Plots with bold white labels are included in this analysis. (E) From each plot, either one (for single‐species plots) or two (for 16‐species plots) planting lines were selected for mapping. For each line, strips of 20 × 200 m (for single‐species plots) or 30 × 200 m (for 16‐species plots) were spatially mapped, determined by the distance between two adjacent planting lines (approximately 10 m) and the length of the planting line (200 m). Within each strip, all enrichment planted seedlings (green points), and surrounding matrix dipterocarp (red points) and non‐dipterocarp (blue points) trees with a DBH ≥ 10 cm, were spatially mapped.

The Sabah Biodiversity Experiment (Hector et al. 2011; Tuck et al. 2016) covers a 500 ha area in the southern part of the 35,000 ha Malua Forest Reserve in Sabah, Malaysian Borneo (05°05′20″N, 117°38′32″E, 102 m above sea level; Figure 1). This selectively logged forest is publicly owned and managed by Yayasan Sabah (The Sabah Foundation). Initial logging within the Malua Forest Reserve between 1984 and 1986 yielded an estimated pre‐logging timber volume of 193–221 m^3^ ha^−1^, predominantly composed of dipterocarps at 180–216 m^3^ ha^−1^ (Saner et al. 2012). The Malua Forest Reserve, except for the Sabah Biodiversity Experiment site, was relogged in 2007 (Wu et al. 2020). The region experiences an aseasonal climate with (as recorded in Danum Valley from 1986 to 2010) a mean annual temperature of 26.9°C, a mean daily minimum of 22.6°C, and a mean daily maximum of 31.3°C (O'Brien et al. 2024). The mean annual rainfall averaged 2854.7 mm (±90.5 s.e.), with monthly means fluctuating between 157.5 mm (April) and 315.1 mm (January) (O'Brien et al. 2024). The experimental site has a 0°–20° range in topography and the soil is classified as orthic acrisol, which is acidic (pH 5–6), highly weathered, and generally low in both nutrient content (81% base saturation) and organic carbon content (topsoil: 1.2%, and at 1 m depth: 0.6%; Saner et al. 2012).

### 2.2 Experimental Design

The Sabah Biodiversity Experiment has a randomised block design comprising 124 four‐hectare plots (200 m × 200 m) divided into a northern and southern block (60 and 64 plots respectively) separated by an old logging road (Figure 1d). Plot size was chosen to ensure that recommendations would be useful to policymakers and so that post‐logging forest conditions among plots would be more spatially independent (Tuck et al. 2016). Of these plots, 112 were randomly assigned to one of four treatments where individual seedlings were positioned along planting lines cleared within plots: enrichment planting with seedlings of one dipterocarp species (*n* = 32), four species (*n* = 32), 16 species (*n* = 32), and 16 species with ‘climber cutting’ (*n* = 16), where all lianas were removed by cutting at their base. Plots enrichment planted with 4 or 16 species used an equal number of seedlings of each species. The remaining plots were left as unplanted, naturally regenerating controls (*n* = 12).

Within each enrichment planted plot 20 parallel lines (running north to south) were cleared at 10 m intervals. Planting lines were cleared of vegetation to a width of two metres. A seedling was planted every three metres producing 66 planting points per line in flat areas (slightly more on uneven ground as needed to complete the planting line). After establishment, vegetation was re‐cut 1 m on either side of each line to allow monitoring of survival and growth. Following standard enrichment planting procedures, a first cohort of seedlings was planted (July 2002 to September 2003), followed by monitoring and planting of a second cohort to replace mortality (from January 2009 to July 2010 after assembling a collection of seedlings). Seedlings for replanting were grown in an on‐site nursery supplied with seedlings from local mast fruiting events. Approximately 44% of planting positions were replanted with a replacement seedling (based on a subset of more intensively monitored plots: 3, 5, 8, 11, 14, and 17).

### 2.3 Study Species

From a pool of 25 species available in the INFAPRO seedling nursery in Danum Valley, 16 species from the Dipterocarpaceae were selected for Sabah Biodiversity Experiment (Table A1). These species were chosen because they represent those found in the surrounding forest, cover a range of traits and ecological strategies, and were available in sufficient quantities locally at the time of planting (Tuck et al. 2016). To help provide practical recommendations, the Sabah Biodiversity Experiment used INFAPRO enrichment planting techniques. These seedlings were collected throughout the Ulu Segama‐Malua Forest Reserve, with the exception of *Hopea ferruginea* (from a seedling nursery south of Danum Valley at Lawasong, Sabah) and *Dipterocarpus conformis* (Tawau Hills area, Sabah). Seedlings were grown from seed from the 1998/99 masting and were around 2 years old at the time of planting. Before planting, seedlings were kept in ~5% light (Tuck et al. 2016). Seedlings were chosen to be as similar in size as possible: Initial sizes varied between species ranging from approximately 100 cm tall and 10 mm in basal diameter for larger seeded species (e.g., *Shorea macrophylla*), to approximately 20 cm tall and 4 mm in basal diameter for smaller seeded species (e.g., *Hopea ferruginea*, *Shorea gibbosa*). Enrichment planted seedlings are individually tagged.

### 2.4 Mapping of Seedlings and Matrix Trees

Because of the large size (500 ha) of the Sabah Biodiversity Experiment most data has been collected on the enrichment planted trees only. To date, it has only been possible to survey all enrichment planted seedlings in all plots on 3 occasions (2004, 2012, 2023). However, to provide more fine‐grained information on growth and survival more frequent surveys have been performed on subsets of plots. The properties and influence of the naturally occurring vegetation are less well documented. Saner et al. (2012) describes the naturally occurring background vegetation based on five transect lines (10 × 100 m) and compares it with matched transects in the old growth forest of nearby Danum Valley Conservation Area but did not survey enrichment planted seedlings. The data presented in this paper is the first to simultaneously measure both enrichment‐planted and naturally occurring trees in Sabah Biodiversity Experiment. Due to the logistical constraints of working at a remote tropical location and the large (500 ha) size of Sabah Biodiversity Experiment, it was only possible to survey a subset of the plots (in the western part of the southern block closer to the project Malua field camp).

We spatially mapped transects in 24 plots: 16 enrichment‐planted with a single species (one of each study species), and eight plots enrichment planted with a mixture of all 16 species. We mapped one planting line in single‐species plots and two adjacent planting lines in 16‐species plots. Two lines were mapped in 16‐species plots to increase the sample size for each individual species in the 16‐species plots, given the lower frequency of each species relative to single‐species plots. In single species plots, we spatially mapped circular areas of 10 m radius centred on each seedling along a single line 200 m in length (i.e., within rectangular strips of approximately 20 × 200 m; Figure 1e). In 16‐species plots, the circular mapped areas were contained within strips of 30 × 200 m, that encompassed two planting. Due to features of the challenging terrain, occasionally planting lines could not be exactly 10 m apart. In these few cases where the neighbouring line was significantly closer (< 5 m) to the target line, we also mapped beyond the planting line to maintain a constant mapped area 10 m in radius. Data on naturally occurring trees was filtered to only include measurements within a 10 m radius of an enrichment planted seedling. The choice of a 10 m radius aligns with the methodologies of comparable studies (e.g., Fortunel et al. 2016). The mapping process was carried out (by MS, JK, RM, MR and the team of Sabah Biodiversity Experiment research assistants) using a ground‐based laser Field‐Map system (Institute of Forest Ecosystem Research (IFER) Ltd., Jílové u Prahy, Czech Republic; Hédl et al. 2009) to obtain relative x, y, and z coordinates for each tree. The FieldMap system combines an Impulse 200 Standard laser rangefinder (equipped with a tilt sensor for vertical angle measurements), a MapStar Module II electronic compass (both from Laser Technology Inc., Colorado, USA), and the specialised mapping software Field‐Map v.11 (IFER, Czech Republic). Two rounds of mapping were begun in December 2012 and May 2015 with the survival and difference in size over this interval used as the response variables in our analyses (see below). Basal diameter was measured for all enrichment planted seedlings at 10 cm above the seedling base, and DBH was measured for naturally occurring trees at 130 cm above the tree base. The naturally occurring trees were identified as dipterocarps or non‐dipterocarps (it was not possible to identify them all in greater detail for this sub‐project). During 2013, percentage canopy openness was surveyed above each enrichment planted seedling using a canopy densiometer that uses a gridded concave mirrored surface to quantify the number of cells of canopy versus sky (Lemmon 1956). To better characterise canopy cover at any given point and avoid directional bias, four replicate measures were taken facing in the different cardinal directions.

### 2.5 Vegetation Matrix Variable Calculations

For each planted seedling, we quantified four properties of the surrounding naturally occurring (i.e., unplanted trees > 10 cm DBH within a 10 m radius). First, for each planted seedling we recorded the percentage canopy openness as the mean measurements from repeat canopy densiometer measurements (see above). Second, for each enrichment planted seedling we calculated the total basal area of all surrounding trees (which could be considered a proxy for local competition). Third, the proportion of the total basal area that belonged to dipterocarps surrounding each seedling was calculated (which could be considered a proxy for confamilial local competition). Finally, we calculated the proportion of the total surrounding basal area that belonged to the single largest tree within a 10 m radius of a planted seedling (which could be considered as a proxy for asymmetric local competition).

### 2.6 Statistical Analysis

We used linear (LMMs) and generalised linear mixed‐effects models (GLMMs) to assess the impact of the naturally occurring vegetation characteristics (including their interactions) on the growth and survival of planted seedlings between the 2012 and 2015 sampling periods. Of the 853 enrichment planted seedlings sampled, we analysed the response of 721 seedlings for analysis with information on all variables (i.e., to exclude the influence of those with incomplete information). Growth was calculated as relative growth rate (RGR), defined as the difference in log‐transformed basal diameter (mm) over time (years). To facilitate model fitting by reducing the chance of non‐convergence and singularities, canopy openness and total basal area were log‐transformed, and the latter was centred after transformation, and each value of total surrounding basal area had the mean total surrounding basal area subtracted from it, effectively making the new mean total surrounding basal area value zero. Our initial model formula (Wilkinson and Rogers 1973) for survival (*Model 1*) was:

$$\begin{array}{c} \mathrm{cohort}+\mathrm{richness}+\mathrm{openness}+p\_\mathrm{dip}+p\_\mathrm{ba}\_\max+\mathrm{ba}\_\mathrm{total}\\ +\mathrm{ba}\_\mathrm{total}:p\_\mathrm{dip}+\mathrm{ba}\_\mathrm{total}:p\_\mathrm{ba}\_\max+\left(1|\mathrm{plot}\right)+\left(1|\mathrm{species}\right) \end{array}$$

where *cohort* is a fixed factor of two levels (planting cohort 1 or 2), *richness* is a factor with two levels (single‐species or 16‐species mixtures), and o*penness* (canopy openness), *p_dip* (proportion dipterocarps), *p_ba_max* (proportion largest individual), and *ba_total* (total basal area of all trees) are covariates. Two variables separated by a colon indicate an interaction (e.g., *ba_total:p_dip*), and factors within parentheses–(1|*Factor*)–indicates random intercepts for levels of the specified factor. We used a GLMM with a binomial error distribution (variance function) to model variation in the binary mortality data (*Model 2*). The model for growth was identical except for the use of a Gaussian error distribution to model the continuous variation in the log‐transformed difference in size (RGR) in a linear mixed‐effects model. The sample size for the analysis of RGR was smaller than for survival because it was only possible to calculate relative growth rates for the subset of individuals alive in the 2015 census. Seedling cohort (1 or 2) was included in the analysis of both survival and growth to assess whether the cohorts behaved similarly or differently and to account for this aspect of our experimental design. *Plot* (a random factor with 24 levels) and *species* (a random factor with 16 levels) were fitted with random intercepts to reflect the structure of the data.

Due to the limited sample size for each species and because we only measured one single‐species plot for each species (which gives reduced information on individual species responses from this analysis compared to earlier analyses of a larger number of plots), we opted to treat species identity as a random effect for the purposes of this analysis to take advantage of the shrinkage through partial pooling that can help when sample sizes for sub‐sets of data (in our case species with low survival rates) are small. Consequently, this analysis places more emphasis on the general response of enrichment planted dipterocarp seedlings and has less ability (lower statistical power) to identify species‐specific differences than some of our previous analyses with larger sample sizes (Tuck et al. 2016). Interactions between the total basal area and both the proportion dipterocarps and proportion largest individual. We might have expected nonlinear relationships for some of our variables, such as canopy openness, however, exploratory plots suggested relationships were approximately linear. Correlations between canopy openness, total surrounding matrix area, proportion largest individual and proportion dipterocarps were carried out to detect potential multicollinearity. Variables were excluded sequentially from models based on Bayesian Information Criterion (BIC). Confidence intervals were estimated using bootstrapping, and we used the Bayesian information criterion (BIC) for model comparison (Schwarz 1978). All analysis was done in R (version 4.3.1; R Core Team 2023) using the ‘lme4’ package (version 1.1–34; Bates et al. 2015). Complete details of the analysis, including exploratory plots, sample sizes by species, model diagnostics, and outputs, are available as an R Markdown document and accompanying pdf file in the Data S1.

## 3 Results

### 3.1 Enrichment Planted Seedlings Versus Naturally Occurring Vegetation

**FIGURE 2:**
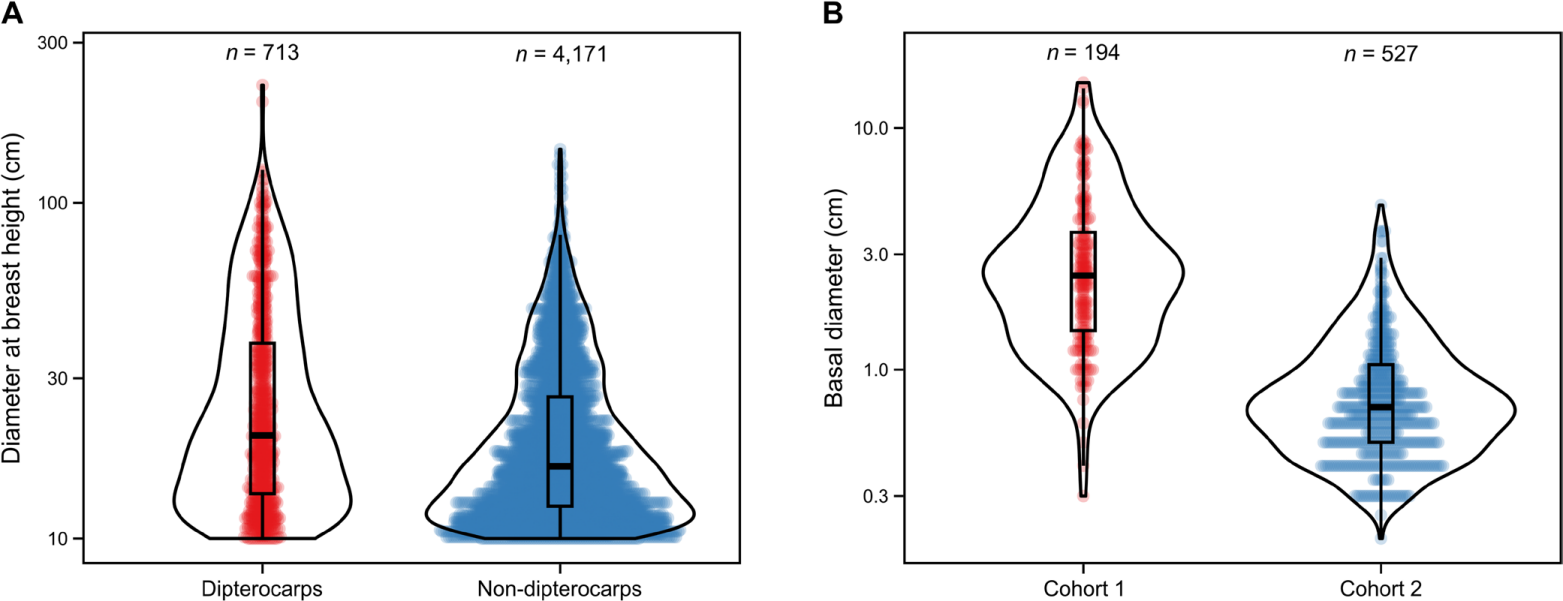
Comparisons of the sizes of surrounding dipterocarp and non‐dipterocarp tree matrix, and between planted dipterocarp seedlings. (A) The diameter at breast height (DBH) of dipterocarp (red) and non‐dipterocarp (blue) trees with DBH ≥ 10 cm within a 10 m radius of all planted seedlings. (B) Basal diameter of planted seedlings from cohort 1 (red) and cohort 2 (blue), categorised by their basal diameter (cm). Boxes encompass the 25th and 75th percentiles, whiskers extend to 1.5 times the interquartile range, and horizontal lines indicate the median. The number of individuals that make up each group (*n*) are indicated above each boxplot.

The majority of enrichment planted dipterocarp seedlings had basal diameters less than 10 cm (basal diameter: median = 0.9 cm; mean = 1.5 cm; maximum = 15.4 cm; Figure 2b), with only four seedlings reaching a basal diameter ≥ 10 cm by the end of the observation period. In total, from the 721 enrichment planted seedlings present in 2012, 544 seedlings (75.5%) survived to 2015 while 177 (24.5%) died (Tables A4, A5, A6). Cohort 1 (planted 2002) experienced a higher survival rate from 2012 to 15 than cohort 2 (planted 2008) where 169 of 194 seedlings from cohort 1 (87.1%) survived versus 375 of 527 (71.2%) for cohort 2 (375 seedlings).

**FIGURE A1:**
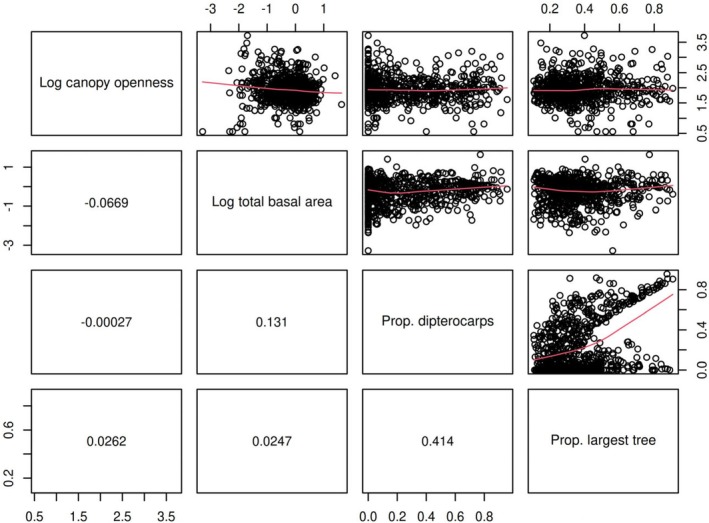
Correlation between combinations of canopy openness (*openness*), total surrounding matrix area (*ba_total*), proportion largest surrounding tree (*p_ba_max*), and proportion dipterocarps (*p_dip*). Statistically significant positive Pearson product–moment correlation coefficients were identified between the proportion largest surrounding tree and proportion dipterocarps (*r*_(719)_ = 0.414, 95% CI = 0.352 to 0.473) as well as between the proportion of trees belonging to dipterocarps and the surrounding total basal area (*r*_(719)_ = 0.131, 95% CI = 0.059 to 0.202).

Of the 4884 naturally occurring trees (> 10 cm DBH) present in the 12.4 ha surveyed area, only 713 (14.6%) were dipterocarps (14.6%), and 4171 (85.4%) were non‐dipterocarps. Among these naturally occurring trees, dipterocarps were generally larger (DBH median = 20.3 cm; mean = 30.2 cm; maximum = 224.0 cm) compared to non‐dipterocarps (median = 16.4 cm; mean = 22.2 cm; maximum = 144.2 cm; Figure 2a). Total basal area of trees (> 10 cm DBH) had a median value of 0.86 m^2^ (Q1 = 0.53 m^2^, Q3 = 1.24 m^2^; Table A2), and the proportion of the total basal area belonging to dipterocarps was low (median = 0.12, Q1 = 0.01, Q3 = 0.44; Table A2). The proportion largest surrounding tree and proportion dipterocarps were positively correlated (*r*_(719)_ = 0.414, 95% CI = 0.352–0.473; Figure A1 and Table A3), as were the proportion of trees belonging to dipterocarps and the surrounding total basal area (*r*_(719)_ = 0.131, 95% CI = 0.059–0.202). Canopy openness values were generally low (median = 6.75%, 1st quartile (Q1) = 5.50%, 3rd quartile (Q3) = 8.75%; Table A2).

As expected, the older, first‐cohort seedlings (2002) generally exhibited larger sizes (basal diameter: median = 24.5 mm; mean = 31.6 mm; range = 3–154 mm; Figure 2b) compared to the younger (2008) second cohort seedlings (median = 7.0 mm; mean = 8.7 mm; range = 2–48 mm; Figure 2b), although there were several instances of size overlap. Some cohort 1 seedlings had attained large basal diameters by 2015 (maximum = 154 mm), although the majority remained smaller.

### 3.2 Survival Analysis

**FIGURE 3:**
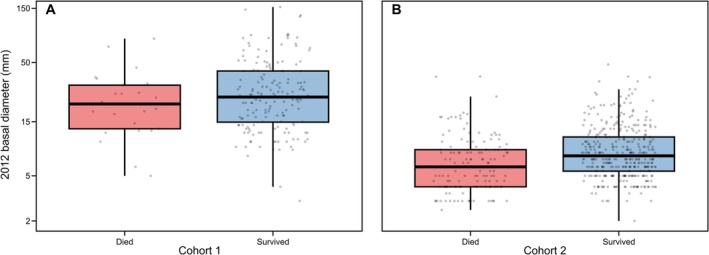
Sizes of seedlings that survived (blue) or died (red) between 2012 and 2015 for (A) cohort 1 and (B) cohort 2. Seedling size was measured as the basal diameter (mm) of planted seedlings in 2012. Boxes encompass the 25th and 75th percentiles, whiskers extend to 1.5 times the interquartile range, and horizontal lines indicate the median. Note that basal diameter is plotted on a log axis.

**FIGURE 4:**
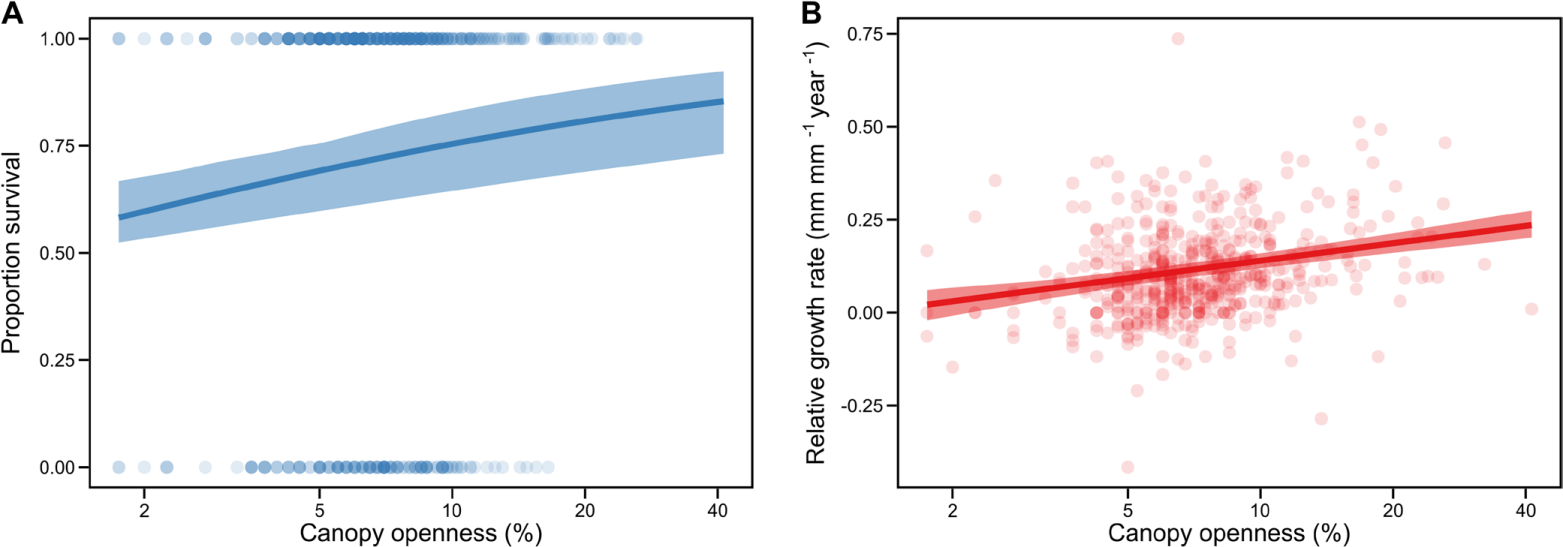
Estimated (A) survival and (B) relative growth rate (RGR) of dipterocarp seedlings in relation to canopy openness. Both predictions are made for cohort 2 seedlings, as they represent 527 of our 721 total seedlings and the observed relationship is conserved between cohorts (Figure A2). All other covariates are held at their mean values. Coloured polygons represent the 95% confidence interval obtained through bootstrapping, and points depict the observed survival and RGR for individual enrichment planted seedlings. For both plots, *n* = 527.

**FIGURE 5:**
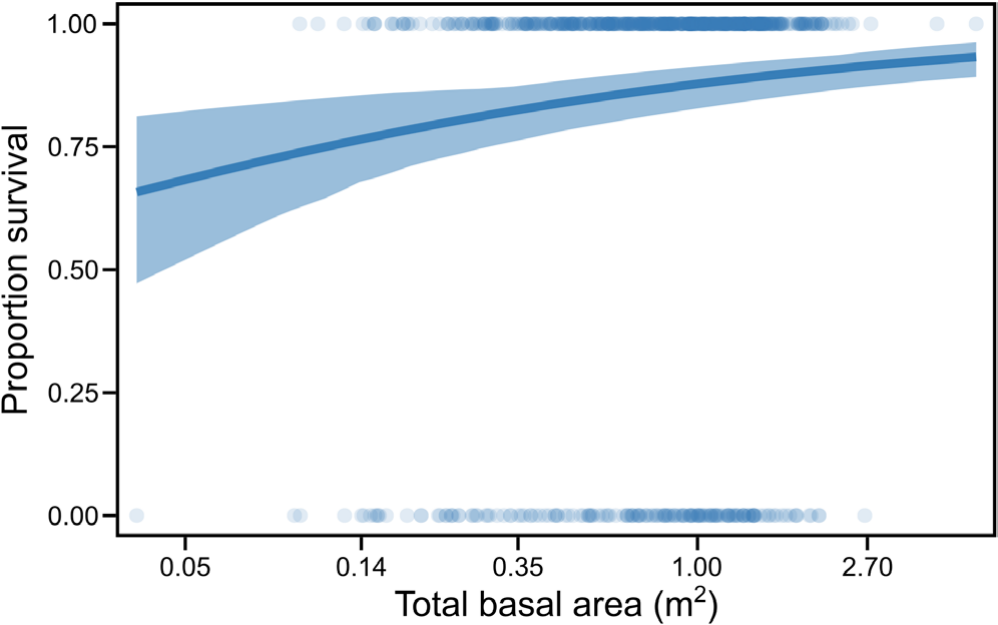
Predicted survival of dipterocarp seedlings in relation to the total basal area of matrix trees within a 10 m radius. Predictions are for cohort 2 seedlings, as they represent 527 of our 721 total seedlings and the observed relationship is conserved between cohorts (Figure A3), and canopy openness is held at its mean value. The blue polygon represents the 95% CI obtained through bootstrapping, and individual points depict the observed survival of planted seedlings. *n* = 527.

**FIGURE A2:**
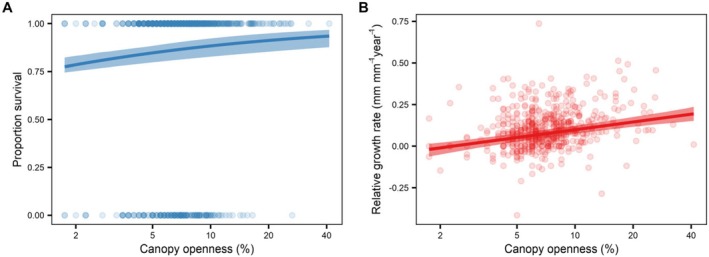
Estimated (A) survival and (B) RGR of dipterocarp seedlings in relation to canopy openness for cohort 1. All other covariates are held at their mean values. Coloured polygons represent the 95% confidence interval obtained through bootstrapping, and points depict the observed survival and RGR for individual enrichment‐planted seedlings. For both plots, *n* = 194.

**FIGURE A3:**
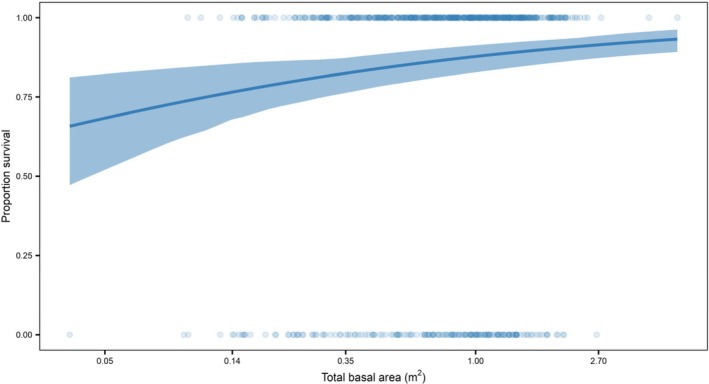
Estimated survival of dipterocarp seedlings in relation to the total basal area of matrix trees within a 10 m radius. Predictions are for cohort 1 seedlings, and canopy openness is held at its mean value. The blue polygon represents the 95% CI obtained through bootstrapping, and individual points depict the observed survival of planted seedlings. *n* = 194.

Model simplification for survival (*Model 1*), retained cohort, canopy openness, and total basal area within 10 m, whilst proportion dipterocarps (estimated change in log‐odds of survival per unit increase = −0.21, 95% CI = −0.92–0.51) and proportion largest surrounding tree (0.046, 95% CI = −0.17–0.11) were omitted. Older seedlings from cohort 1 exhibited higher 2012–2015 survival rates compared to younger seedlings from cohort 2 (mean survival rate with 95% CI at mean canopy openness and total basal area values: cohort 1 = 0.87, 0.82–0.92; cohort 2 = 0.72, 0.68–0.77). Within cohorts, seedlings that survived to 2015 tended to be larger in 2012 than those that died during the. 2012–15 interval (Figure 3). Canopy openness positively affected survival: across our observed range of canopy openness scores, predicted survival increased from approximately 0.58 to 0.86 (Figure 4a and Figure A2a). Basal area of naturally occurring trees had a positive, log‐linear effect on the survival of planted seedlings (Figure 5 and Figure A3), with predicted survival increasing from 0.43 to 0.85 over the observed range of total surrounding basal area values. Variance components for the random effects for plot and species were smaller than the residual error with plot having a slightly larger variance component than species (see supplementary R markdown document).

### 3.3 Growth (Basal Diameter)

**FIGURE A4:**
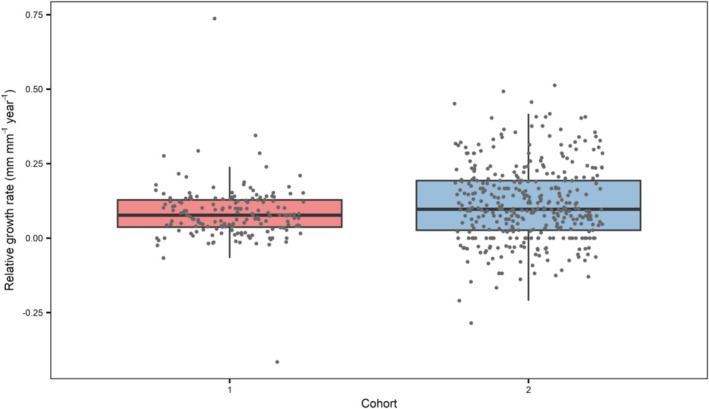
RGR in 2012 for cohorts 1 and 2. Points represent individual seedling values; whiskers extend 1.5 times the interquartile range (boxes). Thick lines are median values.

**FIGURE A5:**
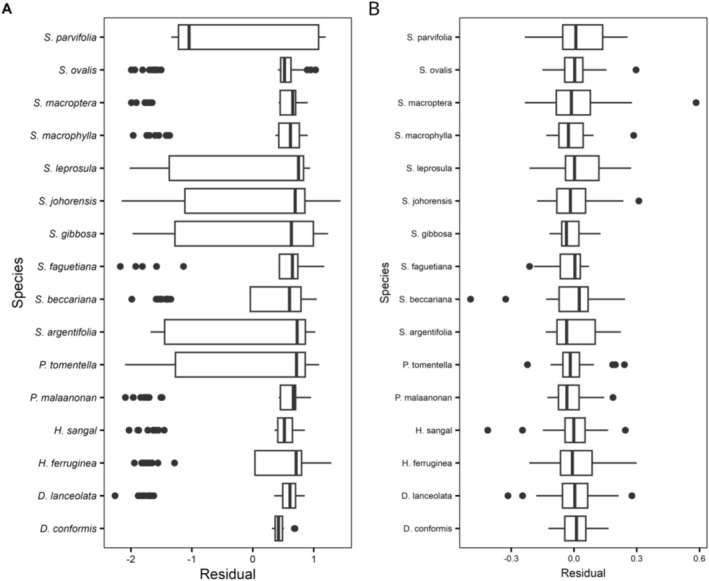
Residual plots for species identity in final survival (A) and growth (B) models. Boxplots contain median, 1st quartile, and 3rd quartiles. Whiskers extend from each quartile to the furthest value no greater than 1.5 times the interquartile range. Data beyond the end of whiskers are plotted as points.

Of the variables included in our initial growth model (*Model 2*), cohort and canopy openness were retained and total basal area (estimated change in RGR per unit increase = 0.011 mm mm^−1^ year^−1^, 95% CI = −0.0049 to 0.028 mm mm^−1^ year^−1^), proportion dipterocarps (−0.033 mm mm^−1^ year^−1^, 95% CI = −0.070 to 0.0037 mm mm^−1^ year^−1^), and proportion largest surrounding tree (0.052 mm mm^−1^ year^−1^, 95% CI = −0.011 to 0.11 mm mm^−1^ year^−1^) were excluded. Seedlings from cohort 2 demonstrated higher growth rates (mean RGR and 95% CI at mean openness value: cohort 1 = 0.076 mm mm^−1^ year^−1^, 0.056–0.10; cohort 2 = 0.12 mm mm^−1^ year^−1^, 0.10–0.14; Figure A4). Canopy openness positively affected growth (Figure 4b and Figure A2b). Across our observed range of canopy openness scores, RGR increased from 0.022 to 0.236 mm mm^−1^ year^−1^. The variance components for the random intercepts for species (SD = 0.04) and plot (SD = 0.02) were relatively small compared to the residual (0.11) (Figure A5).

## 4 Discussion

We investigated how the survival and growth of planted seedlings was related to key factors of the surrounding matrix trees in an enrichment planting experiment. We found that older seedlings planted in the first cohort (2002) had higher survival but lower growth rates than younger seedlings planted in cohort 2 (2008) during the observed period from 2012 to 15, reflecting the expected life‐history trajectory of enrichment planted seedlings where survival initially increases and relative growth rates decline. Canopy openness increased both seedling survival and growth. Our most unexpected results were a positive relationship between the total basal area of surrounding naturally occurring trees and the survival of enrichment planted seedlings, and the lack of a negative relationship between total basal area and growth, which we discuss below.

### 4.1 Seedling Cohort

We found that seedlings from cohort 1 (planted January 2002 to September 2003) had a higher survival rate from December 2012 to May 2015 than seedlings from cohort 2 (planted September 2008 to August 2009). This is expected given the u‐shaped mortality trend with age for most species—cohort 1 seedlings were planted 6 years earlier than cohort 2 and had already suffered a high level of mortality before this study's monitoring period (Tuck et al. 2016), so the surviving seedlings would be the healthiest from cohort 1's initial stock, as well as being generally larger. In contrast, the higher mortality in cohort 2 reflects their shorter time since planting, with potential more recent shock from transplantation (Close et al. 2005), restricted early root growth, and ultimately greater vulnerability to drought (Gilbert et al. 2001). A greater relative growth rate for cohort 2 seedlings than for cohort 1 is also expected, since relative growth rate is known to be generally size‐dependent (Philipson et al. 2012; Turnbull et al. 2012), larger seedlings should have lower relative growth rate values for many reasons (e.g., exhaustion of resources, build‐up of less productive structural tissues, increased self‐shading etc.). Cohort 1 seedlings were generally larger than cohort 2 (Figure 2e,f) and had correspondingly lower relative growth rates (Figure A2).

### 4.2 Canopy Openness

Within the range of canopy openness examined in this study (1.75%–41.25%), enrichment planted dipterocarp survival and growth were positively related to canopy openness, consistent with both previous results from the Sabah Biodiversity Experiment (Philipson et al. 2012, 2014) and with the general consensus that light is a key limiting resource within tropical rainforests (Whitmore and Brown 1996). Unexpectedly, we found no correlation between canopy openness and the total tree basal area. This is likely because areas with high vegetation cover, dominated by pioneers or other plants, were not included in the measurement of the total basal area since they had a DBH of less than 10 cm. Consequently, it is difficult to isolate the effects of light availability from other factors associated with the surrounding vegetation. We might expect the positive relationship to change with higher canopy openness levels than studied here, however, as further increases in light availability may increase drought‐related mortality or hinder the growth of shade‐tolerant species (Adams et al. 2017; O'Brien et al. 2013).

### 4.3 Species Differences

Although our survival modelling revealed no clear overall species‐specific differences on seedling survival (the random effect of *species* explained near‐zero variation within the model) this may reflect the reduced sample size and statistical power of this study (of a subset of trees in a subset of plots) relative to previous studies of the overall Sabah Biodiversity Experiment (e.g., Tuck et al. 2016). There is also evidence in the wider literature of species‐specific relationships between canopy cover and mortality. Qie et al. (2019) showed species‐specific responses to varying levels of degradation of naturally regenerating seedlings within Borneo. These results are similar to other evidence on the capacity of certain species to adapt to environmental conditions in recently logged forests (Clearwater et al. 1999), of which light availability is a primary driver, although Philipson et al. (2014) reported that seedlings did not show large species‐specific survival and growth rate responses to varying light conditions at the Sabah Biodiversity Experiment (Philipson et al. 2014).

In addition to the limited power of our analysis, the observed lack of detectable species‐specific light responses to survival and growth rates could be because the species selected for inclusion at the Sabah Biodiversity Experiment generally comprise shade‐tolerant species with potentially limited functional diversity. Additionally, the relatively homogenous area of the Sabah Biodiversity Experiment relative to the wider Malua Forest Reserve, especially at the within‐plot scale (4 ha), may limit the capacity for species to fully exploit their partitioned niches. In summary, while we do not observe large overall variation between species differences in survival and growth rates in this experiment this should not be over‐interpreted given the limitations of this study, and evidence for species differences from some of our previous analyses of Sabah Biodiversity Experiment and evidence for species differences from the wider literature.

### 4.4 Total Basal Area

The positive relationship between the survival of planted seedlings and total basal area was unexpected as in tropical forests, this relationship is generally negative (Peters 2003) with some variation across species (Murphy et al. 2017). However, for a similar site in Southeast Asia, the relationship was positive when only heterospecifics were considered (Peters 2003). As our site has undergone extensive removal of dipterocarps (Saner et al. 2012), many seedlings were surrounded by no (24.5% of all seedlings) or only a few dipterocarps (median proportion of basal area composed of dipterocarps = 0.11). Therefore, most interactions between planted dipterocarp seedlings and the surrounding naturally occurring trees were predominantly with non‐dipterocarps. At greater densities and higher proportions of dipterocarps, this result could change. However, other results in forests in Vietnam (Nguyen et al. 2016) and China (Chen et al. 2010; Lan et al. 2012) have suggested heterospecific facilitation, although Peters (2003) found no similar pattern in a neotropical sites. One potential direction for future research is to investigate whether the effects of total basal area differ in dipterocarp‐dominated forests of Southeast and East Asia from other tropical forests and, if so, why.

There are alternative explanations for our results, centred on the logging history of the forest. In Borneo, selective logging typically results in a highly heterogeneous mosaic characterised by patches with different levels of logging disturbance. Heavily logged areas have generally unfavourable conditions for seedlings of old‐growth dipterocarp species, particularly for the predominantly shade‐tolerant species planted at the Sabah Biodiversity Experiment. After logging, large trees are replaced by a low stratum of regrowth composed of fast‐growing pioneer species including ferns, grasses, and non‐woody vegetation. This new regrowth (not captured in our study due to the DBH limit of 10 cm) can limit canopy openness for seedlings whilst not providing a microclimatic buffer (Santos et al. 2024) and facilitating increased temperature and decreased humidity levels relative to old‐growth forests (Blonder et al. 2018) and could directly limit aboveground biomass accumulation (O'Brien et al. 2019). Heavily logged areas may also have legacy soil conditions due to vehicle or log landing‐related soil compaction (Hattori et al. 2013). Clark and Clark (2000) showed that both soil type and topography influenced stem size, stand density, and spatial heterogeneity of stems, and Gourlet‐Fleury et al. (2011) more recently showed that soil texture, depth, and hydrology constrained the amount of biomass stored in tropical moist forests. Separately, ectomycorrhizal fungi are known to impact the growth of dipterocarp trees (Saner et al. 2011; Turjaman et al. 2005), and have not yet been intensively surveyed across our sampled lines.

These effects could explain the positive relationship between seedling survival and total tree basal diameter if the study site is a mosaic of patches ranging from those that are heavily logged with low total basal area and poor seedling survival to less intensively logged patches that retain high basal area (and a higher proportion of dipterocarps) with higher survival of planted seedlings. In addition, the relatively consistent effects across two cohorts planted several years apart is also consistent with this patch mosaic hypothesis. The positive relationship of seedling survival with total basal area at this relatively early stage does not mean that the relationship could not reverse at a later stage as species interactions change as secondary succession after logging and replanting continues.

### 4.5 Implications for Enrichment Planting

Our findings have complex implications for enrichment planting within Southeast Asia. On the one hand, the positive associations of planted seedling survival and growth with canopy openness is expected and suggests that planted seedlings may do well in logged areas so long as they have suitable levels of canopy openness (dipterocarps as a relatively shade tolerant group do not generally do well in very open conditions). There is increasing recognition of the negative impacts of intensive selective logging on naturally regenerating tree mortality with moves towards reduced impact logging and similar less intensive systems (Kvasnica et al. 2023). On the other hand, we found seedling survival and growth to be highest in areas with high basal density of naturally occurring trees including a higher proportion of dipterocarps. This is consistent with planted seedlings doing better in lightly logged areas.

This complex picture emerges due to the lack of a clear negative relationship between canopy openness and total basal area (which one could naively expect to be negative if more basal area were positively related with lower levels of canopy openness). This may be a genuine effect (e.g., the patch mosaic scenario discussed above) or it may reflect the complex three‐dimensional light environment and the inability of simple metrics like canopy openness to fully capture the conditions experienced by planted seedlings. For example, although areas in need of restoration would often be expected to have higher levels of canopy openness, short seedlings may nevertheless experience shading from low layers of naturally regrowing shrubs and non‐woody vegetation that are not captured by standard tree surveys (e.g., stems > 10 cm DBH). This suggests the need for better methods for capturing the light conditions experienced by seedlings.

In general, this suggests that the areas where restoration works best may be those that least require it, where the impacts of logging are not too severe. However, there are large areas of selectively logged forests in existence which were logged under intensive schemes. It may therefore be necessary to tolerate lower survival and growth rates to restore the most degraded areas. To offset such a reduced planting success, higher planting densities may be required where mortality has occurred, but this will come with increased cost implications for decision‐makers.

## Author Contributions

**Charles J. Marsh:** conceptualization (equal), data curation (equal), formal analysis (equal), funding acquisition (equal), investigation (equal), visualization (equal), writing – review and editing (equal). **Ryan Veryard:** conceptualization (equal), data curation (equal), formal analysis (equal), funding acquisition (equal), investigation (equal), visualization (equal), writing – original draft (equal), writing – review and editing (equal). **Martin Svátek:** data curation (equal), funding acquisition (equal), investigation (equal), project administration (equal), writing – review and editing (equal). **Elena Fernandez‐Miranda Cagigal:** supervision (equal), writing – review and editing (equal). **Elia Godoong:** writing – review and editing (equal). **Jakub Kvasnica:** investigation (equal), writing – review and editing (equal). **Radim Matula:** writing – review and editing (equal). **Michael J. O'Brien:** conceptualization (equal), data curation (equal), investigation (equal), project administration (equal), writing – review and editing (equal). **Martin Rejžek:** writing – review and editing (equal). **Edgar C. Turner:** conceptualization (equal), supervision (equal), writing – review and editing (equal). **Andy Hector:** conceptualization (equal), formal analysis (equal), funding acquisition (equal), supervision (equal), writing – original draft (equal), writing – review and editing (equal).

## Funding

This work was supported by: Natural Environment Research Council grants NE/S007474/1 (RV), NE/K016458/1 (CM & AH) and NE/K016253/1 (AH). Agencia Estatal de Investigación de España (Ramon y Cajal, RYC2021‐032049‐I) (MOB). Ministry of Education, Youth and Sports of the Czech Republic (grant number: INTER‐TRANSFER LTT17017; MS, JK, MR, RM).

## Conflicts of Interest

The authors declare no conflicts of interest.

## Supporting information


**Data S1:** ece373439‐sup‐0001‐SupplementaryMaterial.pdf.

## Data Availability

All data needed to evaluate the conclusions in the paper are present in the paper and the Supporting Information—S1. The data for this study are published on Zenodo via the SEARRP Research Database community account and can be found via https://doi.org/10.5281/zenodo.13346029.

## Appendix A

**TABLE A1:** The sixteen dipterocarp species investigated at the Sabah Biodiversity Experiment. Note that the Dipterocarpaceae tribe Shoreae underwent a major taxonomic revision in 2022 (Ashton and Heckenhauer 2022). Both old species name are listed alongside each revised name in the below table to help readers connect current species names to those used in earlier publications at the Sabah Biodiversity Experiment.

Species name used in prior Sabah Biodiversity Experiment publications	Current species name	IUCN status	Date of census (year and status from prior assessment if available)
*Dipterocarpus conformis* Slooten	*Dipterocarpus conformis* Slooten	Endangered	2019
*Dryobalanops lanceolata* Burck	*Dryobalanops lanceolata* Burck	Least Concern	2019 (1998 EN)
*Hopea ferruginea* Parijs	*Hopea ferruginea* Parijs	Critically Endangered	1998
*Hopea sangal* Korth.	*Hopea sangal* Korth.	Vulnerable	2017 (1998 CR)
*Parashorea tomentella* Meijer	*Parashorea tomentella* (Symington) Meijer	Least Concern	2017
*Parashorea malaanonan* (Blanco) Merr.	*Parashorea malaanonan* (Blanco) Merr.	Least Concern	2019 (1998 CR)
*Shorea argentifolia* Sym.	*Rubroshorea argentifolia* (Symington) P.S.Ashton and J.Heck.	Least Concern	2019 (1998 EN)
*Shorea beccariana* Bruck	*Rubroshorea beccariana* (Burck) P.S.Ashton and J.Heck.	Least Concern	2019
*Shorea faguetiana* F.Heim.	*Richetia faguetiana* (F.Heim) P.S.Ashton and J.Heck.	Endangered	2017 (1998 EN)
*Shorea gibbosa* Brandis.	*Richetia gibbosa* (Brandis) P.S.Ashton and J.Heck.	Critically Endangered	1998
*Shorea johorensis* Foxw.	*Rubroshorea johorensis* (Foxw.) P.S.Ashton & J.Heck.	Critically Endangered	1998
*Shorea leprosula* Miq.	*Rubroshorea leprosula* (Miq.) P.S.Ashton and J.Heck.	Near Threatened	2017 (1998 EN)
*Shorea macrophylla* Ashton	*Rubroshorea macrophylla* (de Vriese) P.S.Ashton and J.Heck.	Least Concern	2019 (1998 VU)
*Shorea macroptera* King	*Rubroshorea macroptera* (Dyer) P.S.Ashton and J.Heck.	Least Concern	2017
*Shorea ovalis* Korth.	*Rubroshorea ovalis* (Korth.) P.S.Ashton and J.Heck.	Least Concern	2017
*Shorea parvifolia* Dyer.	*Rubroshorea parvifolia* (Dyer) P.S.Ashton and J.Heck.	Least Concern	2017

**TABLE A2:** Distribution of values for investigated covariates. For each studied covariate, the minimum and maximum observed values are provided, as well as the 1st and 3rd quartiles and median values.

Covariate	Minimum	1st quartile	Median	3rd quartile	Maximum
Canopy openness	1.75	5.50	6.75	8.75	41.25
Total basal area	0.04	0.53	0.86	1.24	5.10
Proportion dipterocarp	0.00	0.01	0.12	0.44	0.96
Proportion largest tree	0.11	0.24	0.34	0.46	0.91

**TABLE A3:** Correlation estimates (*r*) and 95% confidence intervals (CI) between the covariates included in this study. *df* = 719.

		*r*	2.5% CI	97.5% CI
Proportion dipterocarp	Proportion largest tree	0.414	0.352	0.473
	Basal area (logged and centred)	0.131	0.059	0.202
	Canopy openness (log scale)	−0.0003	−0.073	0.073
Proportion largest tree	Basal area (logged and centred)	0.025	−0.048	0.098
	Canopy openness (log scale)	0.026	−0.047	0.099
Basal area (logged and centred)	Canopy openness (log scale)	−0.067	−0.139	0.0061

**TABLE A4:** Number of individuals alive at the census in December 2012 by cohort.

Species	Cohort		Total
	1	2	
*Dipterocarpus conformis*	17	2	19
*Dryobalanops lanceolata*	7	62	69
*Hopea ferruginea*	5	47	52
*Hopea sangal*	28	35	63
*Parashorea tomentella*	13	48	61
*Parashorea malaanonan*	10	52	62
*Shorea argentifolia*	1	18	19
*Shorea beccariana*	24	28	52
*Shorea faguetiana*	7	17	24
*Shorea gibbosa*	8	29	37
*Shorea johorensis*	7	48	55
*Shorea leprosula*	10	26	36
*Shorea macrophylla*	14	22	36
*Shorea macroptera*	4	38	42
*Shorea ovalis*	37	23	60
*Shorea parvifolia*	2	32	34

**TABLE A5:** Number of individuals alive at the census of May 2015, by cohort.

Species	Cohort		Total
	1	2	
*Dipterocarpus conformis*	17	2	19
*Dryobalanops lanceolata*	6	51	57
*Hopea ferruginea*	4	34	38
*Hopea sangal*	25	29	54
*Parashorea tomentella*	11	32	43
*Parashorea malaanonan*	8	42	50
*Shorea argentifolia*	1	12	13
*Shorea beccariana*	23	16	39
*Shorea faguetiana*	5	13	18
*Shorea gibbosa*	4	18	22
*Shorea johorensis*	6	29	35
*Shorea leprosula*	5	21	26
*Shorea macrophylla*	13	15	28
*Shorea macroptera*	4	31	35
*Shorea ovalis*	34	13	47
*Shorea parvifolia*	2	14	16

**TABLE A6:** Number of individuals initially surveyed (December 2012), by plot planted species richness.

Species	Planted species richness		Total
	1	16	
*Dipterocarpus conformis*	9	10	19
*Dryobalanops lanceolata*	43	26	69
*Hopea ferruginea*	30	22	52
*Hopea sangal*	45	18	63
*Parashorea tomentella*	26	35	61
*Parashorea malaanonan*	38	24	62
*Shorea argentifolia*	11	8	19
*Shorea beccariana*	36	16	52
*Shorea faguetiana*	15	9	24
*Shorea gibbosa*	22	15	37
*Shorea johorensis*	29	26	55
*Shorea leprosula*	19	17	36
*Shorea macrophylla*	23	13	36
*Shorea macroptera*	24	18	42
*Shorea ovalis*	37	23	60
*Shorea parvifolia*	29	5	34
