# Correction to “Interactions Between Enrichment Planted Seedlings and Naturally Occurring Trees in Selectively Logged Lowland Dipterocarp Forest”

**DOI:** 10.1002/ece3.73880

**Published:** 2026-07-08

Marsh, C. J. , R. Veryard , M. Svátek , et al. 2026. “Interactions Between Enrichment Planted Seedlings and Naturally Occurring Trees in Selectively Logged Lowland Dipterocarp Forest.” Ecology and Evolution 16, 5: e73439. 10.1002/ece3.73439.42083650 PMC13135865

The affiliation of Martin Svátek and Martin Rejžek was incorrect. The correct affiliation is:

Faculty of Forestry and Wood Technology, Mendel University in Brno, Brno, Czech Republic.

The online version of this article has been corrected accordingly.

We apologize for this error.

