# Supplementary material for: Interactions Between Enrichment Planted Seedlings and Naturally Occurring Trees in Selectively Logged Lowland Dipterocarp Forest
Source: Ecol Evol. 2026 May 3;16(5):e73439. doi: 10.1002/ece3.73439 (PMC13135865; doi:10.1002/ece3.73439)
Supplement: Supplementary file 1 — Data S1: ece373439‐sup‐0001‐SupplementaryMaterial.pdf. [file ECE3-16-e73439-s001.pdf]

The impact of surrounding mature trees on dipterocarp seedling growth and survival
Supplementary material - analysis

Ryan Veryard et al.

05 January, 2026

Contents

1 Load packages **1**

2 Settings **2**

3 Loading data **3**

4 An overview of the data: **3**

5 Research questions **6**

6 Exploratory plots **6**
6.1 Line survey of plots . . . 6
6.2 Total area around trees . . . 8
6.3 Summarise seedling characeristics . . . 12
6.4 Surrounding matrix trees . . . 13
6.5 Seedling survival . . . 16
6.6 Seedlings growth rates . . . 18
6.7 Relationships between seedlings and matrix variables . . . 25

7 Analysis **35**
7.1 Survival model fitting . . . 35
7.2 Growth model fitting . . . 40

8 Results **47**
8.1 Survival . . . 47
8.2 Growth . . . 52

9 Session information **55**

1 Load packages

```
library(dplyr)
library(tidyr)
library(tibble)
library(stringr)
```

```r
library(forcats)
library(broom.mixed)
library(sf)
library(RColorBrewer)
library(gridExtra)
library(ggplot2)
library(patchwork)
library(plotrix)
library(lme4)
library(lattice)
library(blmeco)
library(MuMIn)
library(arm)
library(faraway)
```

2 Settings

Global settings for figures:

```r
### Theme for markdown document
theme_doc <- theme_bw() +
  theme(panel.background = element_blank(),
        panel.border     = element_rect(fill = NULL, colour = "black", linewidth = 0.5),
        strip.background = element_rect(fill = "white", colour = "black", linewidth = 0.5),
        panel.grid       = element_blank(),
        axis.ticks       = element_line(colour = "black", linewidth = 0.5),
        title            = element_text(family = "Arial", colour = "black", size = 12),
        axis.text        = element_text(family = "Arial", colour = "black", size = 7),
        axis.title       = element_text(family = "Arial", colour = "black", size = 10),
        strip.text       = element_text(family = "Arial", colour = "black", size = 8),
        legend.text      = element_text(family = "Arial", colour = "black", size = 10),
        legend.title     = element_text(family = "Arial", colour = "black", size = 10))

theme_set(theme_doc)

### Theme for saving figures in journal specifications
theme_pdf <- theme_bw() +
  theme(panel.background = element_blank(),
        panel.border     = element_rect(fill = NULL, colour = "black", linewidth = 0.5),
        strip.background = element_rect(fill = NULL, colour = "black", linewidth = 0.5),
        panel.grid       = element_blank(),
        axis.ticks       = element_line(colour = "black", linewidth = 0.5),
        title            = element_text(family = "Arial", colour = "black", size = 8),
        axis.text        = element_text(family = "Arial", colour = "black", size = 6),
        axis.title       = element_text(family = "Arial", colour = "black", size = 8),
        strip.text       = element_text(family = "Arial", colour = "black", size = 8),
        legend.text      = element_text(family = "Arial", colour = "black", size = 6),
        legend.title     = element_text(family = "Arial", colour = "black", size = 7))

### Plot colours
cols_2 <- c(brewer.pal(6, "Set1")[1:5], brewer.pal(7, "Set1")[7])

### Set seed for standardisating randomisations
```

```r
set.seed(999)
```

3 Loading data

```r
seedlings <- readRDS("Fieldmap_dataset.rds")
```

4 An overview of the data:

```r
dim(seedlings)
```

```
## [1] 721  76
```

```r
str(seedlings)
```

```
## 'data.frame':    721 obs. of  76 variables:
##  $ tree.id            : Factor w/ 721 levels "10.14.11N","10.14.12N",..: 200 214 218 219 221 222 224
##  $ IDPlots            : Factor w/ 24 levels "2","3","4","5",..: 1 1 1 1 1 1 1 1 1 1 ...
##  $ Line_number        : Factor w/ 14 levels "10","11","12",..: 1 1 1 1 1 1 1 1 1 1 ...
##  $ sp.plot            : Factor w/ 126 levels "argentifolia.11",..: 10 10 10 10 10 10 10 10 10 10 ...
##  $ sp.cohort          : Factor w/ 32 levels "argentifolia.1",..: 4 4 4 3 4 3 4 3 3 4 ...
##  $ richness           : Factor w/ 2 levels "mono","sixteen": 1 1 1 1 1 1 1 1 1 1 ...
##  $ X_m                : num  -0.249 -1.333 -1.993 -0.023 -2.158 ...
##  $ Y_m                : num  -0.985 -16.479 -24.872 -10.596 -28.333 ...
##  $ Z_m                : num  0.107 2.42 4.757 0.552 6.12 ...
##  $ genus              : Factor w/ 8 levels "Dipterocarpus",..: 8 8 8 8 8 8 8 8 8 8 ...
##  $ species            : Factor w/ 16 levels "argentifolia",..: 2 2 2 2 2 2 2 2 2 2 ...
##  $ survival           : num  1 1 1 1 0 1 0 1 1 1 ...
##  $ planting.date      : Date, format: "2009-01-01" "2009-01-01" ...
##  $ survey_2002        : Date, format: NA NA ...
##  $ survey_2011        : Date, format: "2012-12-13" "2012-12-13" ...
##  $ survey_2012        : Date, format: "2012-12-06" "2012-12-06" ...
##  $ survey_2015        : Date, format: "2015-05-14" "2015-05-14" ...
##  $ cohort             : Factor w/ 2 levels "1","2": 2 2 2 1 2 1 2 1 1 2 ...
##  $ age_2002           : 'difftime' num  NA NA NA 551 ...
##   ..- attr(*, "units")= chr "days"
##  $ age_2011           : 'difftime' num  1442 1442 1442 3800 ...
##   ..- attr(*, "units")= chr "days"
##  $ age_2012           : 'difftime' num  1435 1435 1435 3793 ...
##   ..- attr(*, "units")= chr "days"
##  $ age_2015           : 'difftime' num  2324 2324 2324 4682 ...
##   ..- attr(*, "units")= chr "days"
##  $ diam_2002          : num  NA NA NA 7.46 NA ...
##  $ diam_2011          : num  7.4 3.7 3.45 30.5 4.05 ...
##  $ diam_2012          : num  18.5 7 6 33 8 26.5 5 49.5 46 5 ...
##  $ diam_2015          : num  41 12 15 44.5 NaN 27 NaN 68.5 45.5 9 ...
##  $ DBH_2002           : num  NA NA NA NaN NA NaN NA NaN NaN NA ...
##  $ DBH_2011           : num  NaN NaN NaN 19.9 NaN ...
##  $ DBH_2012           : num  5 NaN NaN 26 NaN 27.5 NaN 39 18.5 NaN ...
##  $ DBH_2015           : num  31.5 5.5 3 33 NaN 11.5 NaN 56.5 32 8 ...
##  $ rgr_2002           : num  NA NA NA 0.000432 NaN ...
##  $ rgr_2011           : num  0.001941 0.001334 0.001666 0.000428 NaN ...
##  $ rgr_2012           : num  0.000895 0.000606 0.001031 0.000336 NaN ...
```

```
##  $ openness           : num  5.75 4.25 6 5 5.75 5 5 4.5 5.75 5 ...
##  $ ba_total_5         : num  0.0343 0.6065 0.1651 0.3324 0.1495 ...
##  $ ba_total_10        : num  0.268 0.867 0.754 1.087 0.779 ...
##  $ ba_total           : num  0.302 1.473 0.919 1.42 0.928 ...
##  $ ba_mean_5          : num  0.0343 0.1213 0.033 0.1662 0.0374 ...
##  $ ba_mean_10         : num  0.0893 0.0867 0.0838 0.1087 0.0649 ...
##  $ ba_mean            : num  0.124 0.208 0.117 0.275 0.102 ...
##  $ dbh_max_5          : num  209 641 315 641 315 641 559 434 434 228 ...
##  $ dbh_max_10         : num  558 665 559 665 559 665 321 665 641 559 ...
##  $ dbh_max            : num  558 665 559 665 559 665 559 665 641 559 ...
##  $ no_trees_5         : int  1 5 5 2 4 4 2 3 8 4 ...
##  $ no_trees_10        : int  3 10 9 10 12 8 13 13 6 6 ...
##  $ no_trees           : num  4 15 14 12 16 12 15 16 14 10 ...
##  $ ba_total_5_nondip  : num  0 0.607 0.165 0.332 0.15 ...
##  $ ba_total_10_nondip : num  0.268 0.867 0.754 1.017 0.779 ...
##  $ ba_total_nondip    : num  0.268 1.473 0.919 1.349 0.928 ...
##  $ ba_mean_5_nondip   : num  0 0.1213 0.033 0.1662 0.0374 ...
##  $ ba_mean_10_nondip  : num  0.0893 0.0867 0.0838 0.1271 0.0649 ...
##  $ ba_mean_nondip     : num  0.0893 0.208 0.1168 0.2933 0.1023 ...
##  $ dbh_max_5_nondip   : num  0 641 315 641 315 641 559 434 434 228 ...
##  $ dbh_max_10_nondip  : num  558 665 559 665 559 665 321 665 641 559 ...
##  $ dbh_max_nondip     : num  558 665 559 665 559 665 559 665 641 559 ...
##  $ no_trees_5_nondip  : int  0 5 5 2 4 4 2 3 8 4 ...
##  $ no_trees_10_nondip : int  3 10 9 8 12 7 13 13 6 5 ...
##  $ no_trees_nondip    : num  3 15 14 10 16 11 15 16 14 9 ...
##  $ ba_total_5_dip     : num  0.0343 0 0 0 0 ...
##  $ ba_total_10_dip    : num  0 0 0 0.0706 0 ...
##  $ ba_total_dip       : num  0.000343 0 0 0.000706 0 ...
##  $ ba_mean_5_dip      : num  0.0343 0 0 0 0 ...
##  $ ba_mean_10_dip     : num  0 0 0 0.0353 0 ...
##  $ ba_mean_dip        : num  0.0343 0 0 0.0353 0 ...
##  $ dbh_max_5_dip      : num  209 0 0 0 0 0 0 0 0 0 ...
##  $ dbh_max_10_dip     : num  0 0 0 215 0 215 0 0 0 173 ...
##  $ dbh_max_dip        : num  209 0 0 215 0 215 0 0 0 173 ...
##  $ no_trees_5_dip     : int  1 0 0 0 0 0 0 0 0 0 ...
##  $ no_trees_10_dip    : int  0 0 0 2 0 1 0 0 0 1 ...
##  $ no_trees_dip       : num  1 0 0 2 0 1 0 0 0 1 ...
##  $ openness_log       : num  1.75 1.45 1.79 1.61 1.75 ...
##  $ ba_total_log       : num  -1.1966 0.3876 -0.0844 0.3503 -0.0746 ...
##  $ ba_total_log_scaled: num [1:721, 1] -0.926 0.658 0.186 0.621 0.196 ...
##   ..- attr(*, "scaled:center")= num -0.271
##  $ prop_dip           : num  0.1135 0 0 0.0497 0 ...
##  $ ba_max             : num  0.245 0.347 0.245 0.347 0.245 ...
##  $ prop_ba_max        : num  0.809 0.236 0.267 0.245 0.264 ...
```

The data has the following columns:

- `tree.id`: the ID of the tree in question. This takes the format `[plot].[line].[number]` with cohort (Old [`O`] or new [`N`]) at the end.
- `IDPlots`: the plot number the tree in question is located in.
- `Line_number`: the specific line within a plot that the tree in question is located in.
- `sp.plot`: a column specifying both the species and the plot number of the tree in question, separated by a period.
- `sp.cohort`: a column specifying both the species and the cohort of the tree in question, separated by a comma.

- richness: if the plot the tree is located in is a plot enrichment-planted with one (mono) or 16 species (sixteen).
- X_m: relative x coordinate - the first position of Field-Map had x,y,z-coordinates (0,0,0).
- Y_m: relative y coordinate.
- Z_m: relative z coordinate.
- genus: the genus-level identity of the seedling.
- species: the species-level identity of the seedling.
- survival: if the seedling was alive by 2015 (1 = alive, 0 = dead).
- planting date: the date of planting of the seedling.
- survey_[year]: the date of the survey for a specified year's census.
- cohort: if this seedling was part of the initial planted cohort planted in 2002 (cohort 1) or the second cohort (cohort 2) which was planted later to replace those that had died.
- age_[year]: the age (in days) of seedlings at the time of their survey in the specified year.
- diam_[year]: basal diameter of seedling in the specified year, measured in mm.
- DBH_[year]: the DBH of seedling in the specified year, measured in mm.
- rgr_[year]: the relative growth rate of seedling, calculated as (ln(diameter in 2015)-ln(diameter in [year]) / (age in days in 2015 - age in days in [year]).
- openess: the average canopy openness score across 4 cardinal directions, taken during the second census.
- ba_total[_n]: the total basal area of surrounding trees, either within 5m (_5), between 5 and 10m (_10), or within 10m (no suffix).
- ba_mean[_n]: the mean basal area of surrounding trees, either within 5m (_5), between 5 and 10m (_10), or within 10m (no suffix).
- dbh_max[_n]: thedbh of the largest single neighbouring tree, either within 5m (_5), between 5 and 10m (_10), or within 10m (no suffix).
- no_trees[_n]: the number of trees either within 5m (_5), between 5 and 10m (_10), or within 10m (no suffix).
- note that the previous four main bullets are repeated again for specifically non-dipterocarp surrounding unplanted trees (with columns containing a _nondip suffix), or dipterocarp trees (_dip suffix).
- also note that columns that lack either a _dip or _nondip suffix are given in cm or cm^2, whilst those with a _dip or _nondip suffix are given in mm or mm^2.
- openess_log: the openness score now expressed on a natural log scale.
- ba_total_log: the ba_total score now expressed on a natural log scale.
- ba_total_log_scaled: the ba_total_log score but now scaled using the scale() function (center = TRUE, scale = FALSE).
- prop_dip: the proportion of basal area of surrounding trees that belong to dipterocarps.
- ba_max: the basal area of the largest tree, calculated as `pi * ((seedlings$dbh_max / 10) ^ 2) / 40000`.
- prop_ba_max: the proportion of the basal area that belongs to the largest single tree.

The tidying process left 721 seedlings in total. 24 plots have been measured; 16 single-species plots and eight 16-species plots. Note that six of the eight 16-species plots are the Sabah Biodiversity Experiment's 'intensive monitoring plots', which have been monitored much more frequently than the experiment has. There are a few other things to note, which may have relevance in the analyses:

- There is only one measurement for each single-species plot, and so species effects are confounded with both plot and richness.
- Plots can be made up of any combination of 1st cohort seedlings, 2nd cohort seedlings, or both.
- 16-species plots were sampled across two lines, less than the number of lines sampled for single-species plots. This means that there may be less environmental variation sampled within each 16-species plot than within each single-species plot.
- Relative growth rates are measured between the two high-resolution mapping measures in 2012 and 2015 as 2002 census measurements do not include the 2nd cohort seedlings and the 2011 census occurred over many months.

5 Research questions

For this study, our initial research questions are:

1. What is the effect of total surrounding matrix density (the intensity of local competition) on the growth and survival of seedlings?
2. What is the effect of the proportional of basal area that is dipterocarp (confamilial density) on the growth and survival of seedlings? And how does the proportion of surrounding dipterocarps impact the relationship between surrounding matrix density and growth and survival?
3. What is the effect of size (proportion of surrounding area belonging to the largest tree) on the growth and survival of seedlings? And how does the proportion of the maximum tree impact the relationship between surrounding matrix density and growth and survival?
4. How does canopy openness affect the growth and survival of enrichment-planted seedlings?

6 Exploratory plots

6.1 Line survey of plots

Plots 2, 3, 4, 5, 7, 8, 10, 11, 13, 14, 17, 18, 22, 23, 27, 34, 37, 43, 44, 50, 52, 59, 61, 62 were mapped. Whilst single-species enrichment-planted plots were mapped along a single line, plots planted with 16 species were mapped over two to ensure as many species were sampled as possible. We can visualise these planting lines:

6.1.1 Intensively-surveyed plots

As the intensively-surveyed plots (3, 5, 8, 10, 14, and 17) are typically involved in more research than other plots at the Sabah Biodiversity Experiment, we can view those here as well:

```
seedlings %>%
  filter(IDPlots %in% c("3", "5", "8", "11", "14", "17")) %>%
  ggplot(aes(X_m, Y_m, colour = Line_number)) +
  theme_doc +
  theme(legend.position = "none",
        axis.title.x=element_blank(),
        axis.title.y=element_blank()) +
  facet_wrap(.~ IDPlots, scales = "free_y", ncol = 6) +
  scale_color_brewer(palette = "Set1") +
  geom_point()
```

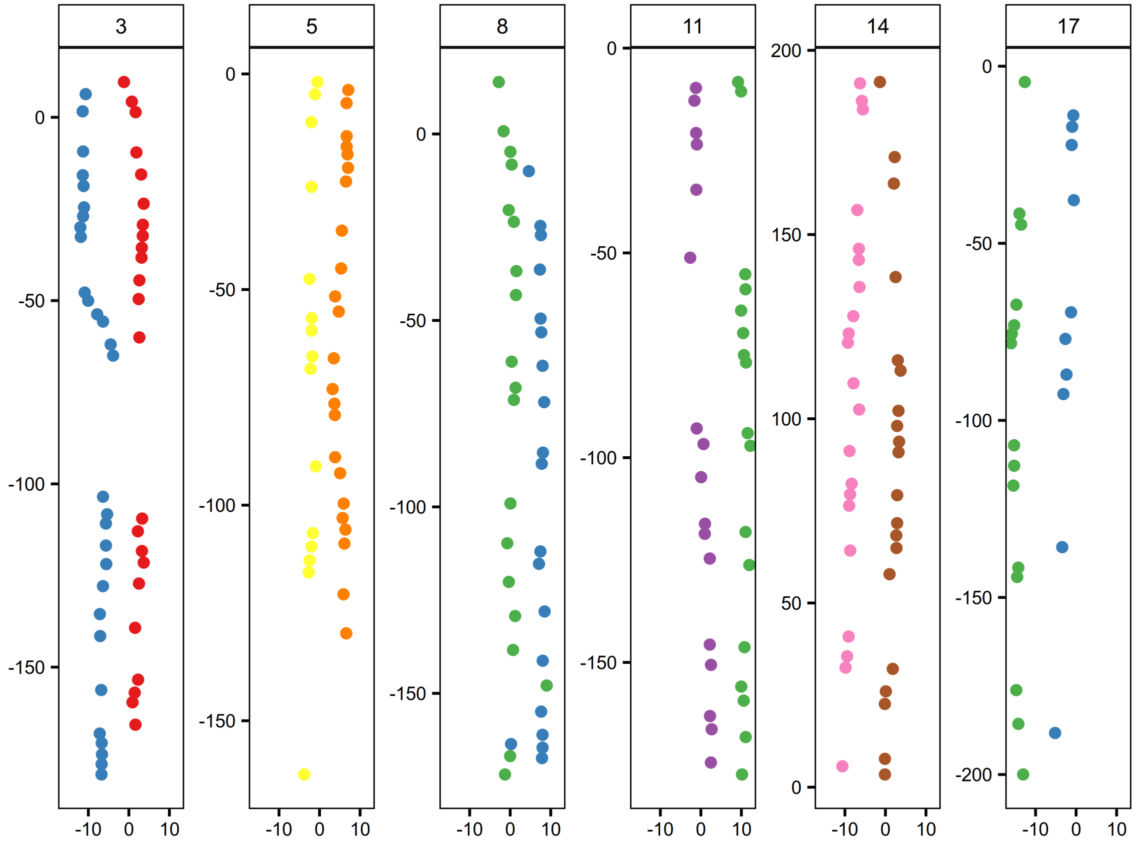

Focussing on just one plot, we can visualise all the trees too:

```
planting_locations <- read.csv("SBE2012_2015.csv") %>%
  filter(is.na(Standing_dead2012)) %>%
  filter(IDPlots == 4) %>%
  mutate(type = case_when(Dipterocarp == "seedling" ~ "Planted",
                          Dipterocarp == 0          ~ "Natural: non-dipterocarp",
                          Dipterocarp == 1          ~ "Natural: dipterocarp",
                          .default = "Planted")) %>%
  dplyr::select(IDPlots, ID_field, X_m, Y_m, Source_natural_planted, Dipterocarp, type)%>%
  mutate(X_m = X_m - min(X_m),
         Y_m = Y_m - min(Y_m))

# Define a custom function to add "m" unit
add_unit <- function(x) {
  paste0(x, " m")
}

example_line <- planting_locations %>%
  ggplot(aes(Y_m, X_m, colour = type )) +
  theme_doc +
  theme(legend.position = "bottom") +
  coord_fixed() +
  scale_x_continuous(labels = add_unit, name = "Y") +
  scale_y_continuous(labels = add_unit, breaks = c(0,10,20), name = "X") +
  scale_colour_brewer(palette = "Set1") +
  geom_point()
```

```
example_line
```

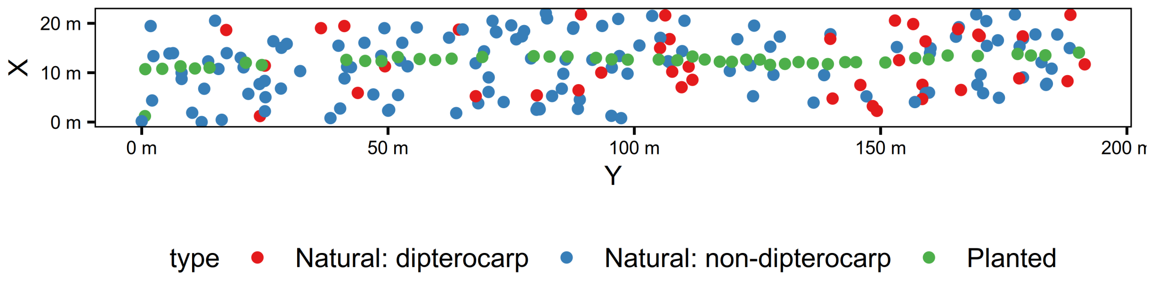

6.2 Total area around trees

We can calculate the total area covered by a 10 m search radius around all of our trees for each plot, taking into account the potential overlap in areas between species:

```
# Define the tree coordinates
tree_coordinates <- seedlings %>%
  dplyr::select(IDPlots, X_m, Y_m) %>%
  rename("x" = "X_m", "y" = "Y_m")
rownames(tree_coordinates ) <- NULL

# Define the constant coordinate position
# (any coordinate works here, so I've just chosen some for Oxford)
latitude  <- 51.7522200
longitude <- -1.2559600

tree_coordinates <- data.frame(
  x = tree_coordinates$x / 111111 + latitude,  # 1 degree latitude ~ 111111 meters
  y = tree_coordinates$y / (111111 * cos(latitude * pi/180)) + longitude,
  IDPlots = tree_coordinates$IDPlots
)

plot_ids <- as.numeric(as.character(unique(tree_coordinates$IDPlots)))

# Create an empty list to store the results and iterate over each plot
results <- list()
for (plot_id in plot_ids) {
  # Subset the data for the current plot
  plot_data <- subset(tree_coordinates, IDPlots == plot_id)

  # Define the tree coordinates for the current plot as a spatial data frame
  tree_coordinates_current <- data.frame(
    x = plot_data$x,  # Example x-coordinates of trees for the current plot
    y = plot_data$y   # Example y-coordinates of trees for the current plot
  )

  # Define the tree coordinates as a spatial data frame
  tree_sfc <- st_as_sf(tree_coordinates_current, coords = c("x", "y"))
  st_crs(tree_sfc) <- 4326  # Set the coordinate reference system (CRS)
```

```
  # Buffer the trees with a 10-meter radius
  tree_buffer <- st_buffer(tree_sfc, dist = 10)

  # Merge overlapping buffers
  merged_buffer <- st_union(tree_buffer)

  # Calculate the total area within the merged buffer
  total_area <- st_area(st_make_valid(merged_buffer))

  # Convert the area to hectares
  # total_area_hectares <- total_area / 10000
  total_area_hectares <- units::set_units(total_area, "ha")

  # Store the result in the list
  results[[plot_id]] <- total_area_hectares
}
results[sapply(results, is.null)] <- NA

results <- data.frame(IDPlots = seq_along(results),
                      area_ha = unlist(results, use.names = FALSE)) %>%
  filter(!is.na(area_ha))

# Print the results for each plot
results
```

```
##    IDPlots   area_ha
## 1        2 0.5841344
## 2        3 0.7501127
## 3        4 0.6180969
## 4        5 0.5527803
## 5        7 0.3806775
## 6        8 0.6829803
## 7       10 0.6198236
## 8       11 0.6192056
## 9       13 0.4739396
## 10      14 0.7364680
## 11      17 0.5551300
## 12      18 0.4720044
## 13      22 0.4621439
## 14      23 0.5057593
## 15      27 0.4835586
## 16      34 0.2564491
## 17      37 0.5962814
## 18      43 0.3142936
## 19      44 0.5541961
## 20      50 0.3787394
## 21      52 0.2273124
## 22      59 0.5491974
## 23      61 0.5146203
## 24      62 0.5283704
```

And we can then calculate the total area surveyed in this work:

```
area_covered <- as.numeric(results %>% summarise(sum(area_ha)))
```

In total, we surveyed 12.42 ha of forest. This estimate seems reasonable, as there are 32 lines surveyed total and each is roughly 200m long. If the surveyed area it was a perfect rectangle that would be 12.8 hectares, so this is a reasonable estimate given the amount of overlap we can see. For instance, here is the area covered by plot 62:

```
plot(merged_buffer)
```

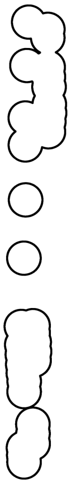

By dividing by the total basal area for dipterocarps and non-dipterocarps we can calculate the basal area per hectare for each:

```
trees <- read.csv("SBE2012_2015.csv") %>%
  filter(Source_natural_planted == "n") %>%
  # trees$ID_field[trees$ID_field == "unknown"] <- NA
  filter(Dipterocarp == 0 | Dipterocarp == 1) %>%
  filter(!is.na(DBH_mm2012)) %>% filter(is.na(Standing_dead2012))

### Dipterocarp area
dip <- sort(trees$DBH_mm2012[trees$Dipterocarp == 1]) / 10
dip <- cumsum(apply(data.frame(min = seq(10, 225, 5), max = seq(15, 230, 5)),
                    1,
                    function(x) {trees <- dip[(dip >= x[1]) & (dip < x[2])]
                    trees <- pi * (trees ^ 2) / 40000
                    sum(trees)}))
dip_density <- max(dip) / area_covered
```

```r
### Non-dipterocarps
nondip <- sort(trees$DBH_mm2012[trees$Dipterocarp == 0]) / 10
nondip <- cumsum(apply(data.frame(min = seq(10, 225, 5), max = seq(15, 230, 5)),
                       1,
                       function(x) {trees <- nondip[(nondip >= x[1]) & (nondip < x[2])]
                       trees <- pi * (trees ^ 2) / 40000
                       sum(trees)}))
nondip_density <- max(nondip) / area_covered
```

This tells us that we have a density of 6.866 square meters per hectare for dipterocarp and 18.97 for non-dipterocarp. Note that this is only for stems with a diameter at breast height greater than 10 cm.

We will also create a table summarising our key matrix variables:

```r
# Canopy openness
open <- seedlings %>%
  summarise(min    = min(openness),
            q1     = quantile(openness, 0.25),
            median = quantile(openness, 0.5),
            q3     = quantile(openness, 0.75),
            max    = max(openness)) %>%
  mutate(type = "Openness")

# Basal area
ba <- seedlings %>%
  summarise(min    = min(ba_total),
            q1     = quantile(ba_total, 0.25),
            median = quantile(ba_total, 0.5),
            q3     = quantile(ba_total, 0.75),
            max    = max(ba_total)) %>%
  mutate(type = "ba_total")

# Proportion dipterocarps
prop_dip <- seedlings %>%
  summarise(min    = min(prop_dip),
            q1     = quantile(prop_dip, 0.25),
            median = quantile(prop_dip, 0.5),
            q3     = quantile(prop_dip, 0.75),
            max    = max(prop_dip)) %>%
  mutate(type = "prop_dip")

# Proportion basak area as largest tree
prop_max <- seedlings %>%
  summarise(min    = min(prop_ba_max),
            q1     = quantile(prop_ba_max, 0.25),
            median = quantile(prop_ba_max, 0.5),
            q3     = quantile(prop_ba_max, 0.75),
            max    = max(prop_ba_max)) %>%
  mutate(type = "prop_ba_max")

table_s2 <- rbind(open, ba, prop_dip, prop_max) %>%
  dplyr::select(type, everything())
table_s2
```

```
##          type         min         q1     median         q3         max
```

```
## 1    Openness 1.75000000 5.5000000 6.7500000 8.7500000 41.2500000
## 2    ba_total 0.03763715 0.5276239 0.8620307 1.2371228  5.1016144
## 3    prop_dip 0.00000000 0.0000000 0.1227028 0.4378817  0.9576114
## 4 prop_ba_max 0.10792747 0.2397908 0.3358136 0.4593149  0.9066919
```

```
### Save for Table A2
write.csv(table_s2, "table_s2.csv", row.names = FALSE, quote = FALSE)
```

6.3 Summarise seedling characteristics

There are 721 measured. 436 seedlings were from single-species plots, and 285 from multi-species plots.

```
seedlings %>%
  mutate(species = paste(genus, species)) %>%
  summarise("Single species" = sum(richness == "mono"),
            "Multi-species"  = sum(richness == "sixteen"),
            "Total"     = n(),
            .by = species)
```

```
##                    species Single species Multi-species Total
## 1        Shorea beccariana             36            16    52
## 2       Shorea macrophylla             23            13    36
## 3     Parashorea tomentella            26            35    61
## 4           Shorea gibbosa             22            15    37
## 5     Parashorea malaanonan            38            24    62
## 6        Shorea johorensis             29            26    55
## 7             Hopea sangal             45            18    63
## 8            Shorea ovalis             37            23    60
## 9      Shorea argentifolia             11             8    19
## 10         Hopea ferruginea            30            22    52
## 11 Dryobalanops lanceolata             43            26    69
## 12        Shorea faguetiana            15             9    24
## 13        Shorea macroptera            24            18    42
## 14         Shorea leprosula            19            17    36
## 15        Shorea parvifolia            29             5    34
## 16 Dipterocarpus conformis              9            10    19
```

194 seedlings were from cohort 1 and 527 from cohort 2.

```
seedlings %>%
  mutate(species = paste(genus, species)) %>%
  summarise("Cohort 1" = sum(cohort == 1),
            "Cohort 2" = sum(cohort == 2),
            "Total"    = n(),
            .by = species)
```

```
##                    species Cohort 1 Cohort 2 Total
## 1        Shorea beccariana       24       28    52
## 2       Shorea macrophylla       14       22    36
## 3     Parashorea tomentella      13       48    61
## 4           Shorea gibbosa        8       29    37
## 5     Parashorea malaanonan      10       52    62
## 6        Shorea johorensis        7       48    55
## 7             Hopea sangal       28       35    63
## 8            Shorea ovalis       37       23    60
## 9      Shorea argentifolia        1       18    19
```

```
## 10        Hopea ferruginea        5       47    52
## 11 Dryobalanops lanceolata        7       62    69
## 12       Shorea faguetiana        7       17    24
## 13       Shorea macroptera        4       38    42
## 14        Shorea leprosula       10       26    36
## 15       Shorea parvifolia        2       32    34
## 16 Dipterocarpus conformis       17        2    19
```

Cohort 1 seedlings are generally much larger than second cohort seedlings:

```
bind_rows(
  seedlings %>%
    summarise(cohort = "All",
              median = median(diam_2012, na.rm = TRUE),
              mean   = mean(diam_2012, na.rm = TRUE),
              se     = std.error(diam_2012, na.rm = TRUE),
              min    = min(diam_2012, na.rm = TRUE),
              max    = max(diam_2012, na.rm = TRUE),
              count  = n()),
  seedlings %>%
    group_by(cohort) %>%
    summarise(median = median(diam_2012, na.rm = TRUE),
              mean   = mean(diam_2012, na.rm = TRUE),
              se     = std.error(diam_2012, na.rm = TRUE),
              min    = min(diam_2012, na.rm = TRUE),
              max    = max(diam_2012, na.rm = TRUE),
              count  = n())
)
```

```
##   cohort median      mean        se min max count
## 1    All    9.0 14.961972 0.6521293   2 154   721
## 2      1   24.5 31.637306 1.8188751   3 154   194
## 3      2    7.0  8.736944 0.2581947   2  48   527
```

6.4 Surrounding matrix trees

In total, 4,884 trees were recorded within 10m of the planted seedlings, with almost 6 times as many non-dipterocarps than dipterocarps:

```
trees <- read.csv("SBE2012_2015.csv") %>%
  filter(Source_natural_planted == "n") %>%
  filter(Dipterocarp == 0 | Dipterocarp == 1) %>%
  filter(!is.na(DBH_mm2012)) %>%
  filter(is.na(Standing_dead2012)) %>%
  mutate(ID_field = case_when(ID_field == "unknown" ~ NA, .default = ID_field))

trees %>%
  mutate(Dipterocarp = ifelse(Dipterocarp == 1, "Dipterocarp", "Non-dipterocarp")) %>%
  group_by(Dipterocarp) %>%
  summarise(total = n()) %>%
  mutate(percent = total * 100 / sum(total))
```

```
## # A tibble: 2 x 3
##   Dipterocarp     total percent
##   <chr>           <int>   <dbl>
## 1 Dipterocarp       713    14.6
```

```
## 2 Non-dipterocarp  4171    85.4
```

Looking at the surrounding matrix trees, dipterocarps appear to be generally larger but at lower frequencies and have an overall lower basal area density:

```
trees %>%
  mutate(Dipterocarp = case_when(Dipterocarp == 1 ~ "Dipterocarp",
                                 Dipterocarp == 0 ~ "Non-dipterocarp")) %>%
  mutate(dbh_2012_cm = DBH_mm2012 / 10) %>%
  group_by(Dipterocarp) %>%
  summarise(median = median(dbh_2012_cm, na.rm = TRUE),
            mean   = mean(dbh_2012_cm, na.rm = TRUE),
            se     = std.error(dbh_2012_cm, na.rm = TRUE),
            min    = min(dbh_2012_cm, na.rm = TRUE),
            max    = max(dbh_2012_cm, na.rm = TRUE),
            count  = n())
```

```
## # A tibble: 2 x 7
##   Dipterocarp     median  mean    se   min   max count
##   <chr>            <dbl> <dbl> <dbl> <dbl> <dbl> <int>
## 1 Dipterocarp       20.3  30.2 0.924    10   224    713
## 2 Non-dipterocarp   16.4  22.2 0.233    10  144.  4171
```

Generate a figure (Figure 2) showing the size distributions of seedlings from different cohorts and dipterocarps vs non-dipterocarps:

```
p1 <- trees %>%
  mutate(Dipterocarp = case_when(Dipterocarp == 1 ~ "Dipterocarps",
                                 Dipterocarp == 0 ~ "Non-dipterocarps")) %>%
  ggplot(aes(y = DBH_mm2012 / 10, x = Dipterocarp)) +
  theme_doc +
  scale_y_log10(limits = c(10, 275)) +
  # coord_flip() +
  labs(y = "Diameter at breast height (cm)", x = NULL) +
   scale_colour_manual(values = c("Dipterocarps" = cols_2[1],
                                  "Non-dipterocarps" = cols_2[2])) +
  ggbeeswarm::geom_beeswarm(aes(colour = Dipterocarp),
                            cex = 0.4, alpha = 0.25, show.legend = FALSE) +
  geom_violin(fill = NA) +
  geom_boxplot(fill = NA, outliers = FALSE, width = 0.08) +
   geom_text(data = data.frame(Dipterocarp = c("Dipterocarps", "Non-dipterocarps"),
                               n = c(sum(trees$Dipterocarp == 1),
                                     sum(trees$Dipterocarp == 0))),
            aes(y = 275, label = paste0('paste(italic("n"), " = ", ', n, ')')),
            parse = TRUE, family = "Arial", colour = "black", size = 6, size.unit = "pt")

# p2 <- all_dips %>%
#   ggplot(aes(y = size_cm, x = Source_natural_planted)) +
#   theme_doc +
#   # coord_flip() +
#   labs(y = "Tree diameter (cm)", x = NULL) +
#   ggbeeswarm::geom_beeswarm(alpha = 0.5,
#                             cex = 0.2, fill = cols_1[1:2]) +
#   geom_violin(alpha = 0.5) +
#   geom_boxplot(alpha = 0.5, outliers = FALSE, width = 0.08)
```

```
p3 <- seedlings %>%
   mutate(cohort = ifelse(cohort == "1", "Cohort 1", "Cohort 2"),
          diam_2012_cm = diam_2012 / 10) %>%
   ggplot(aes(y = diam_2012_cm, x = cohort)) +
   theme_doc +
   scale_y_log10() +
   # coord_flip() +
   labs(y = "Basal diameter (cm)", x = NULL) +
   scale_colour_manual(values = c("Cohort 1" = cols_2[1], "Cohort 2" = cols_2[2])) +
   ggbeeswarm::geom_beeswarm(aes(colour = cohort),
                             cex = 0.4, alpha = 0.25, show.legend = FALSE) +
   geom_violin(fill = NA) +
   geom_boxplot(fill = NA, outliers = FALSE, width = 0.08) +
   geom_text(data = data.frame(cohort = c("Cohort 1", "Cohort 2"),
                               n = c(sum(seedlings$cohort == 1),
                                     sum(seedlings$cohort == 2))),
             aes(y = 20, label = paste0('paste(italic("n"), " = ", ', n, ')')),
             parse = TRUE, family = "Arial", colour = "black", size = 6, size.unit = "pt")

fig2 <- p1 + p3 +
  plot_layout(ncol = 2, axes = "collect_y", tag_level = "new") +
  plot_annotation(tag_levels = "A") &
  theme(plot.tag = element_text(family = "Arial", face = "bold",
                                colour = "black", size = 10))
fig2
```

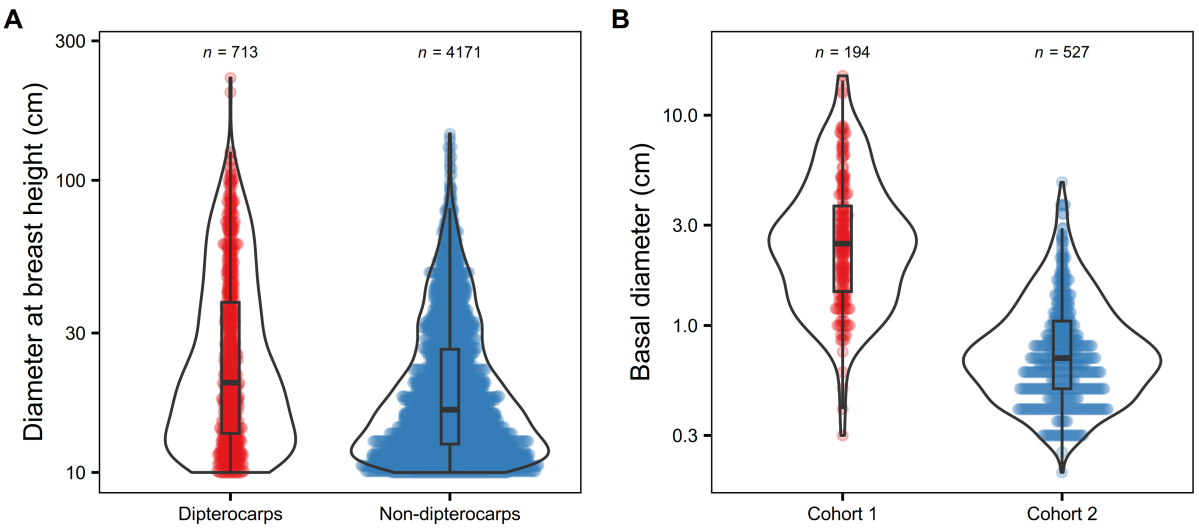


```
fig2 <- fig2 +
  theme_pdf

fig2 <- (p1 + theme_pdf) + (p3 + theme_pdf) +
  plot_layout(ncol = 2, axes = "collect_y", tag_level = "new") +
  plot_annotation(tag_levels = "A") &
  theme(plot.tag = element_text(family = "Arial", face = "bold",
                                colour = "black", size = 10))
```

```r
ggsave("Figures/Final/fig_2.png", ,
       bg = "white", width = 180, height = 75, units = "mm", dpi = 600)

ggsave("Figures/Final/fig_2.pdf", fig2,
       bg = "white", width = 180, height = 75, units = "mm", dpi = 600, cairo_pdf)
```

6.5 Seedling survival

Of the 721 planted seedlings, 177 seedlings died between the two censuses (24.55%), leaving 544 seedlings that survived.

```r
seedlings %>%
  filter(survival == 1) %>%
  mutate(rgr  = rgr_2012 * 365.25) %>% # so now rgr is expressed in years
  filter(!is.na(rgr)) %>%
  mutate(species = paste(genus, species)) %>%
  summarise("Cohort 1" = sum(cohort == 1),
            "Cohort 2" = sum(cohort == 2),
            "Total"    = n(),
            .by = species)
```

```
##                     species Cohort 1 Cohort 2 Total
## 1         Shorea beccariana       23       16    39
## 2     Parashorea tomentella       11       32    43
## 3            Shorea gibbosa        4       18    22
## 4     Parashorea malaanonan        8       42    50
## 5        Shorea macrophylla       13       15    28
## 6         Shorea johorensis        6       29    35
## 7             Shorea ovalis       34       13    47
## 8       Shorea argentifolia        1       12    13
## 9              Hopea sangal       25       29    54
## 10         Hopea ferruginea        4       34    38
## 11 Dryobalanops lanceolata        6       51    57
## 12        Shorea macroptera        4       31    35
## 13         Shorea leprosula        5       21    26
## 14 Dipterocarpus conformis       17        2    19
## 15        Shorea parvifolia        2       14    16
## 16        Shorea faguetiana        5       13    18
```

In cohort 1 there was an 87.1% survival rate (169 out of 194 seedlings). Cohort 2 had a 71.2% survival rate (275 out of 527 seedlings).

```r
seedlings %>%
  group_by(cohort) %>%
  summarise(total    = n(),
            survived = sum(survival)) %>%
  mutate(percent = survived * 100 / total)
```

```
## # A tibble: 2 x 4
##   cohort total survived percent
##   <fct>  <int>    <dbl>   <dbl>
## 1 1        194      169    87.1
## 2 2        527      375    71.2
```

Seedlings that survived between 2012 and 2015 appear to have been slightly larger in 2012 than those that died:

```r
seedlings %>%
  group_by(cohort, survival) %>%
  mutate(survival = case_when(survival == 0 ~ "Died",
                              survival == 1 ~ "Survived")) %>%
  reframe(median = median(diam_2012, na.rm = TRUE),
          mean   = mean(diam_2012, na.rm = TRUE),
          se     = std.error(diam_2012, na.rm = TRUE),
          min    = min(diam_2012, na.rm = TRUE),
          max    = max(diam_2012, na.rm = TRUE),
          count  = n())
```

```
## # A tibble: 4 x 8
##   cohort survival median  mean    se   min   max count
##   <fct>  <chr>     <dbl> <dbl> <dbl> <dbl> <dbl> <int>
## 1 1      Died       21.5 25.6  3.59    5    81      25
## 2 1      Survived   24.8 32.5  2.01    3   154     169
## 3 2      Died        6    7.3  0.444   2.5  37.5   152
## 4 2      Survived    7.5  9.30 0.310   2    48     375
```

Generate Figure 3 and save:

```r
### Figure 3
### Panel labels
labels <- data.frame(x = 0.5, y = 145,
                     cohort = c("Cohort 1", "Cohort 2"),
                     text = c("A", "B"))

### Plot
fig_3 <- seedlings %>%
  filter(!is.na(diam_2012)) %>%
  mutate(survival = case_when(survival == 1 ~ "Survived",
                              survival == 0 ~ "Died"),
         cohort   = case_when(cohort == 1 ~ "Cohort 1",
                              cohort == 2 ~ "Cohort 2")) %>%
  ggplot(aes(survival, diam_2012, fill = survival)) +
  theme_doc +
  theme(strip.background = element_blank(),
        strip.placement  = "outside",
        legend.position  = "none") +
  facet_wrap(~ cohort, strip.position = "bottom") +
  scale_y_continuous(trans = "log", breaks = c(2, 5, 15, 50, 150)) +
  labs(x = NULL, y = "2012 basal diameter (mm)", title = NULL) +
  scale_fill_brewer(palette = "Set1") +
  geom_boxplot(alpha = 0.5, outlier.shape = NA) +
  geom_jitter(height = 0, width = 0.25, size = 0.5, colour = "black", alpha = 0.25) +
  geom_text(data = labels, aes(x = x, y = y, label = text, fill = NULL),
            family = "Arial", size = 10, size.unit = "pt" , fontface = "bold", colour = "black")

fig_3
```

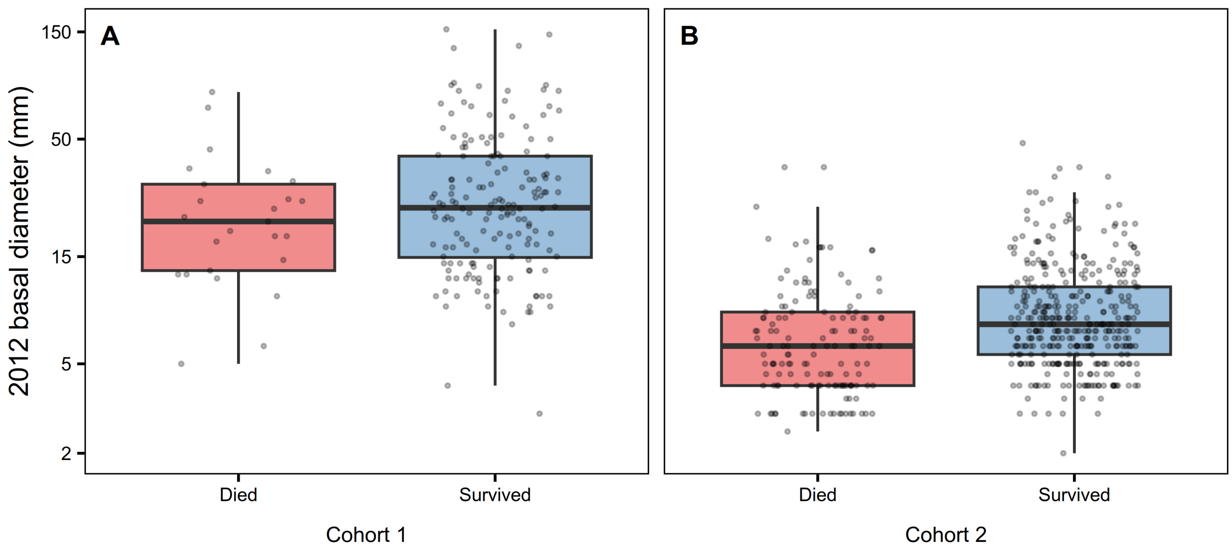


```
### Save
fig_3 <- fig_3 +
  theme_pdf +
  theme(strip.background = element_blank(),
        strip.placement  = "outside",
        legend.position  = "none")

ggsave("Figures/Final/fig_3.pdf", fig_3,
       width = 180, height = 75, units = "mm", bg = "white", dpi = 600, cairo_pdf)

ggsave("Figures/Final/fig_3.png", fig_3,
       width = 180, height = 75, units = "mm", bg = "white", dpi = 600)
```

6.6 Seedlings growth rates

Convert growth rates to mm per year:

```
growth <- seedlings %>%
  filter(survival == 1) %>%
  mutate(rgr  = rgr_2012 * 365.25) %>% # so now RGR is expressed in years
  filter(!is.na(rgr))
```

There are no clear difference can be immediately seen between the RGR values of different cohorts, although cohort 2 does have a slightly larger median value:

```
growth %>%
  group_by(cohort) %>%
  summarise(mean   = mean(rgr, na.rm = TRUE),
            median = median(rgr, na.rm = TRUE),
            se     = std.error(rgr, na.rm = TRUE),
            min    = min(rgr, na.rm = TRUE),
            max    = max(rgr, na.rm = TRUE))
```

```
## # A tibble: 2 x 6
##   cohort   mean median      se    min   max
##   <fct>   <dbl>  <dbl>   <dbl>  <dbl> <dbl>
## 1 1      0.0836 0.0770 0.00710 -0.416 0.737
```

```
## 2 2      0.114  0.0970 0.00659 -0.285 0.513
```

Plot growth rates between cohorts (Figure A1) and save:

```
### Figure A1
fig_s1 <- growth %>%
  mutate(num = row_number()) %>%
  mutate(size = diam_2015 - diam_2012) %>%
  ggplot(aes(cohort, rgr)) +
  theme_doc +
  labs(y = expression(paste("Relative growth rate (mm ",mm^-1, " ",year^-1,")")),
       x = "Cohort") +
  scale_fill_brewer(palette = "Set1") +
  geom_boxplot(aes(fill = cohort), alpha = 0.5, outlier.shape = NA, show.legend = FALSE) +
  geom_jitter(height = 0, width = 0.25, size = 0.5, colour = "grey40", show.legend = FALSE)

fig_s1
```

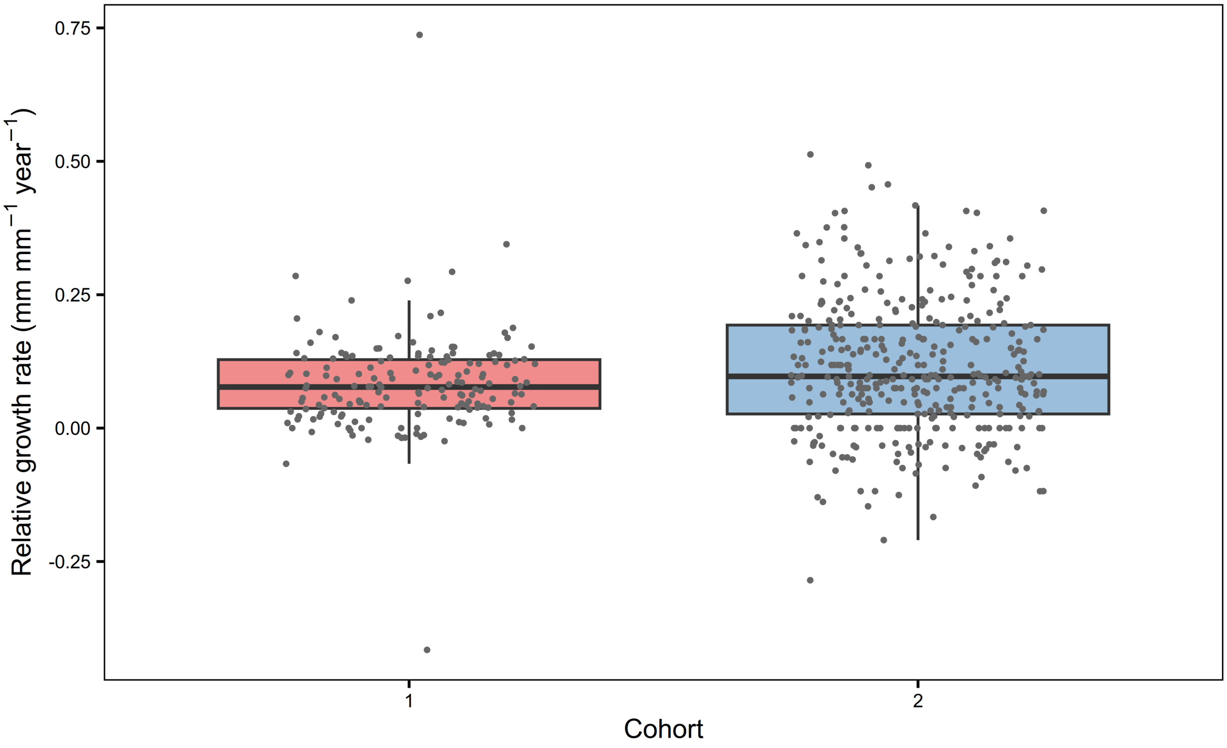


```
### Save the plots
fig_s1 <- fig_s1 +
  theme_pdf

ggsave("Figures/Final/SI/fig_s1.pdf", fig_s1,
       width = 170, height = 100, units = "mm", bg = "white", dpi = 600, device = cairo_pdf)

ggsave("Figures/Final/SI/fig_s1.png", fig_s1,
       width = 170, height = 100, units = "mm", bg = "white", dpi = 600)
```

If we also include species as a facet, we see that, whilst there are some species-specific differences between cohorts, the sample sizes for some cohort x species combinations are too low to make any inferences:

```
growth %>%
  mutate(num = row_number()) %>%
  mutate(size = diam_2015 - diam_2012) %>%
  ggplot(aes(cohort, rgr)) +
  theme_doc +
  theme(legend.position = "none") +
  facet_wrap(~species, scales = "free_y") +
  labs(y = expression(paste("RGR (mm ",mm^-1, " ",yr^-1,")")),
       x = "Cohort") +
  scale_fill_brewer(palette = "Set1") +
  geom_boxplot(aes(fill = cohort), alpha = 0.5, outlier.shape = NA) +
  geom_jitter(height = 0, width = 0.25, size = 0.5, colour = "grey40")
```

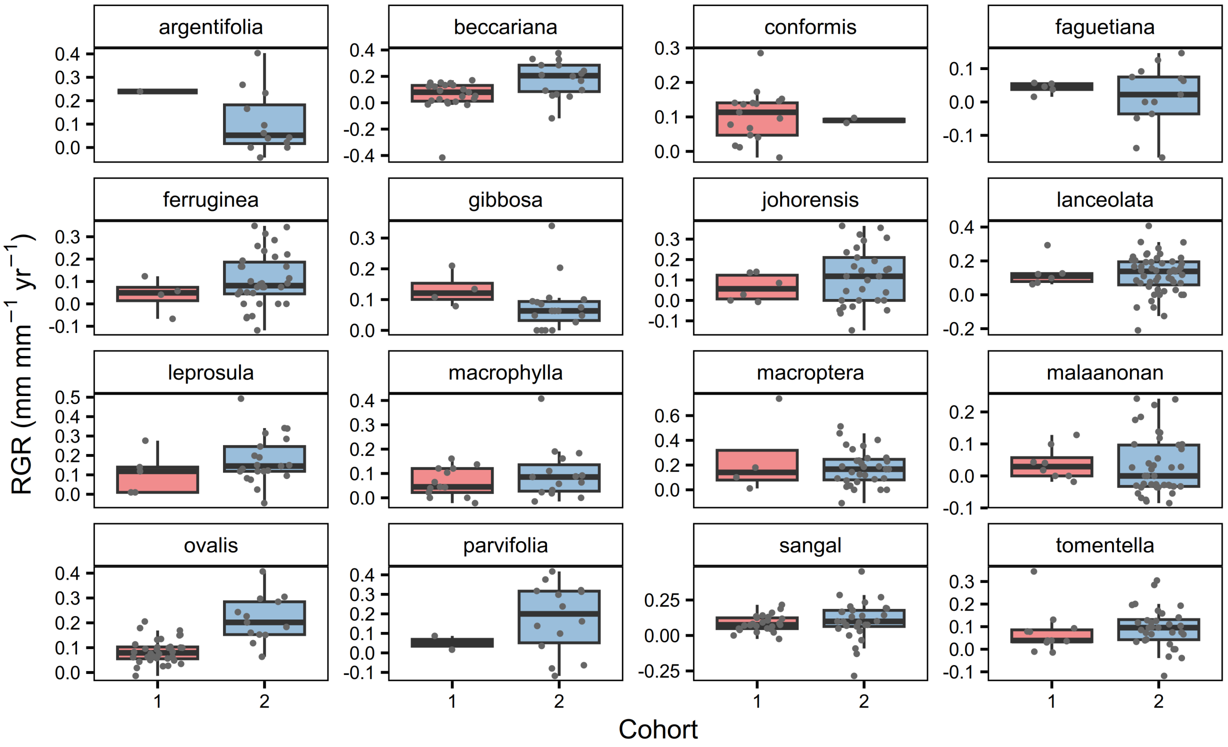

These two plots give a general look into our data. In the next two plots the variables `species` and `IDPlots` are swapped between x axis and facets for the additional perspective.

First by plot:

```
ggplot(seedlings,
       aes(species, rgr_2012, group = cohort)) +
  theme_doc +
  theme(axis.text.x     = element_text(angle = 90, vjust = 0.5, size = 7),
        legend.position = "bottom") +
  facet_wrap(~IDPlots) +
  labs(x = "Species", y = "RGR 2012-2015", colour = "Cohort") +
  geom_hline(aes(yintercept = 0)) +
  scale_colour_brewer(palette = "Set1") +
  geom_point(aes(colour = cohort), alpha = 0.7)
```

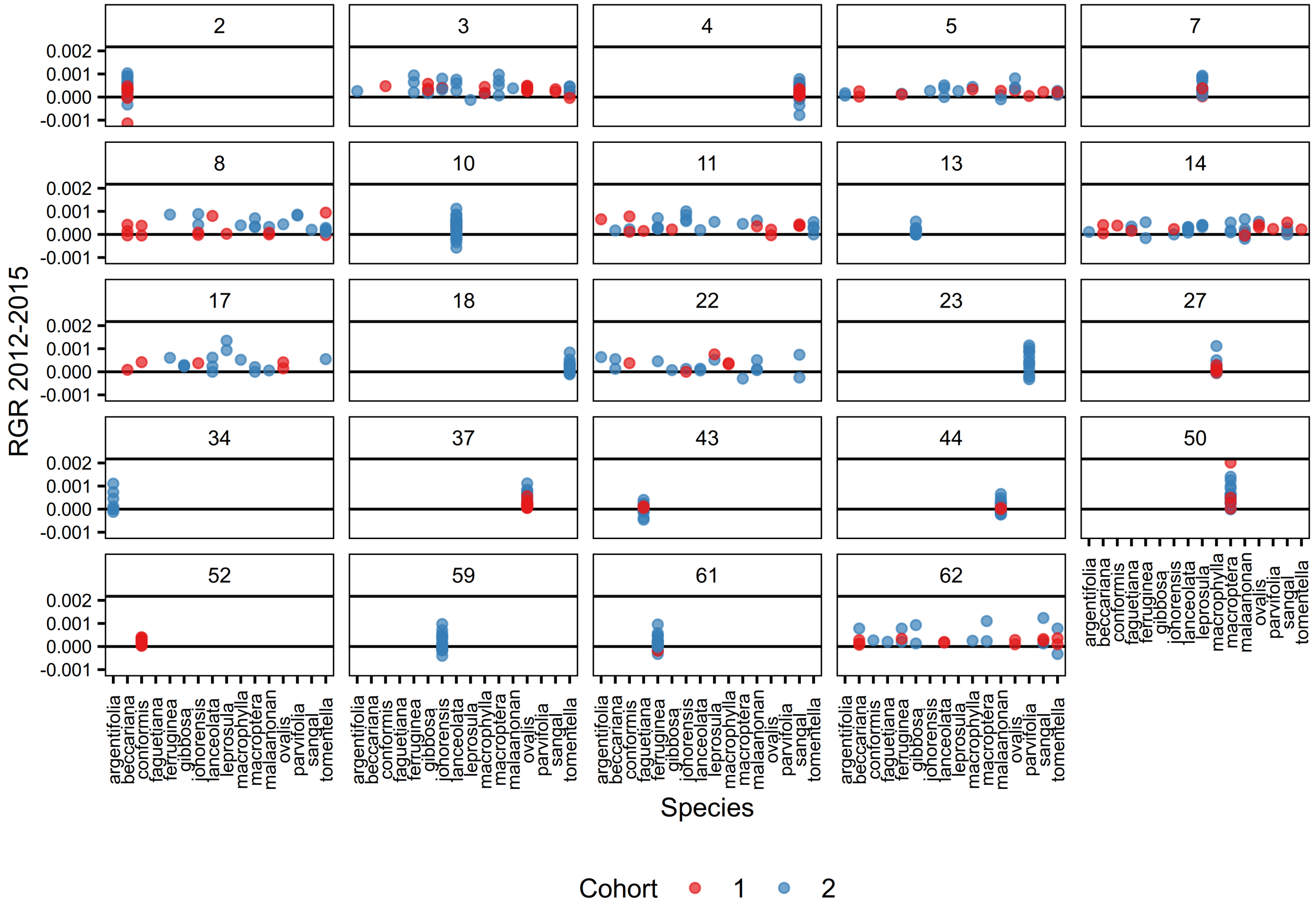

Then by species:

```
ggplot(seedlings,
       aes(IDPlots, rgr_2012, group = cohort)) +
  theme_doc +
  theme(axis.text.x      = element_text(angle = 90, vjust = 0.5, size = 7),
        legend.position = "bottom") +
  facet_wrap(~species) +
  labs(x = "Plot", y = "RGR 2012-2015", colour = "Cohort") +
  geom_hline(aes(yintercept = 0)) +
  scale_colour_brewer(palette = "Set1") +
  geom_point(aes(colour = cohort), alpha = 0.7)
```

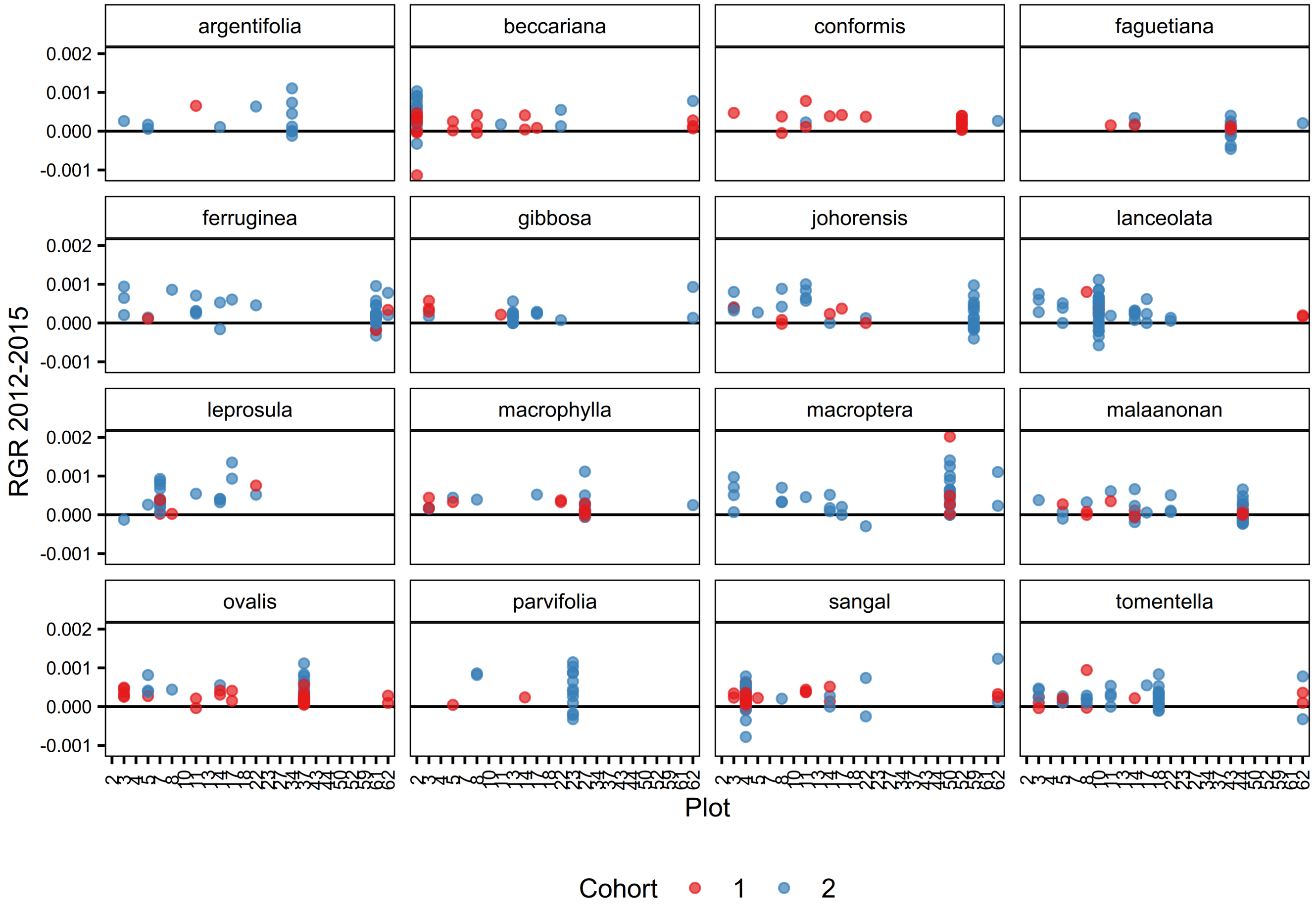

As cohort 2 trees are younger than cohort 1 we should look at the data longitudinally in these initial plots. We cannot find a clear and immediate difference in growth rates between cohorts due to large amount of intergroup variation observed.

```
seedlings_long <- data.frame(id       = rep(seedlings$tree.id, 4),
                             plots    = rep(seedlings$IDPlots, 4),
                             richness = rep(seedlings$richness, 4),
                             species  = rep(seedlings$species, 4),
                             cohort   = rep(seedlings$cohort, 4),
                             age      = c(as.numeric(seedlings$age_2002),
                                          as.numeric(seedlings$age_2011),
                                          as.numeric(seedlings$age_2012),
                                          as.numeric(seedlings$age_2015)),
                             log_diam = log(c(seedlings$diam_2002,
                                              seedlings$diam_2011,
                                              seedlings$diam_2012,
                                              seedlings$diam_2015)))
```

Plot log diameter against seedling age:

```
seedlings_long %>%
  ggplot(aes(age/365.25, log_diam, colour = cohort)) +
  theme_doc +
  facet_wrap(~species) +
```

```r
  scale_x_continuous(breaks = c(2,4,6,8,10,12)) +
  labs(x = "Age (years)", y = "Basal diameter (ln(mm))", colour = "Cohort") +
  scale_colour_brewer(palette = "Set1") +
  geom_point(shape = 21, fill = NA, size = 1) +
  geom_smooth(method = "lm", se = FALSE, size = 0.5, fullrange = TRUE)
```

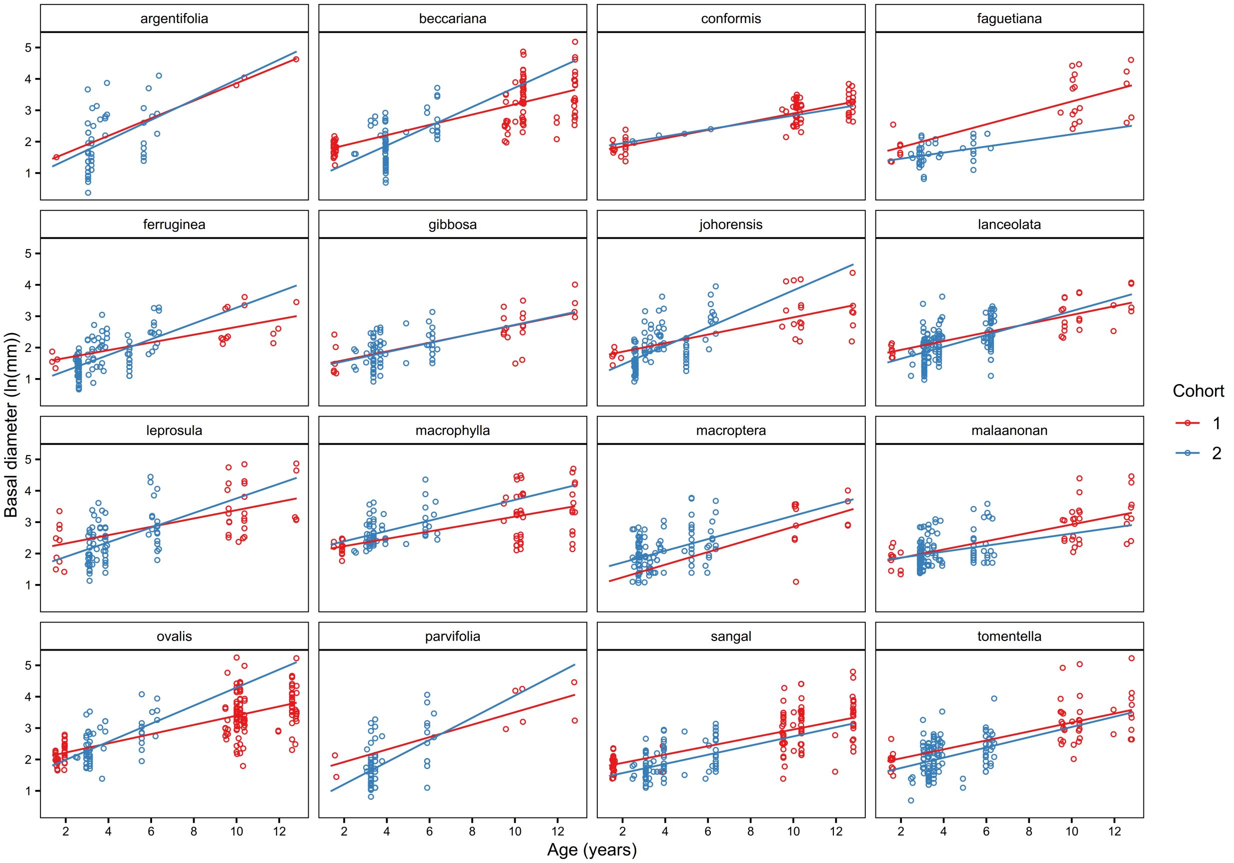

The above plot also highlights the overlap between the 2011 and 2012 measurements due to the long time taken to complete the 2011 measurements. This is one reason why we prefer to use 2012 measurements as a comparative baseline.

Repeating the above plots but now looking at richness (whether plots are enrichment-planted with one or 16 species) reveals no clear effect at a glance. There are potentially more negative values with the single-species enrichment-planted plots, and they may have more variation too, but there is a lot of within-group variation which makes this difficult to determine immediately.

```r
ggplot(seedlings,
       aes(IDPlots, rgr_2012, group = richness)) +
  theme_doc +
  theme(axis.text.x = element_text(angle = 90, vjust = 0.5, size = 8)) +
  facet_wrap(~species) +
  labs(x = "Plot", y = "RGR 2012-2015", colour = "Richness") +
  scale_colour_brewer(palette = "Set1") +
  geom_hline(aes(yintercept = 0)) +
  geom_point(aes(colour = richness), alpha = 0.7)
```

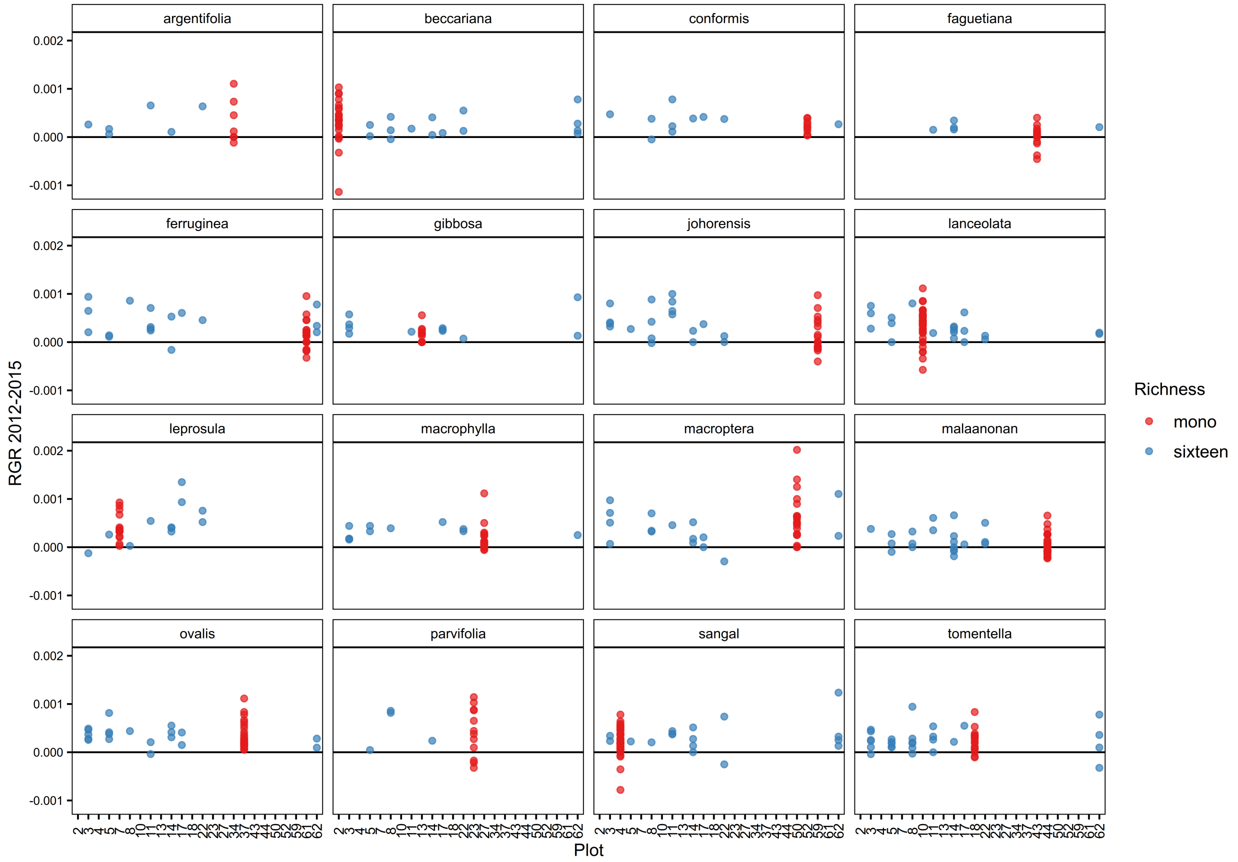

Looking at this longitudinally, we see a similar outcome as we did for cohort. No differences in growth rates between plots can be easily identified. This contrasts with other work done (e.g. Veryard et al. (2023)), but we could attribute this difference to the relatively small sampling period (between 2012 and 2015). It is possible that if there were sufficient size data in 2002 we would see a different result than what is seen here.

```
seedlings_long %>%
  ggplot(aes(age/365.25, log_diam, colour = richness)) +
  theme_doc +
  facet_wrap(~species) +
  scale_x_continuous(breaks = c(2,4,6,8,10,12)) +
  labs(x = "Age (years)", y = "Basal diameter (ln(mm))", colour = "Richness") +
  scale_colour_brewer(palette = "Set1") +
  geom_point(shape = 21, fill = NA, size = 1) +
  geom_smooth(method = "lm", se = FALSE, size = 0.5, fullrange = TRUE)
```

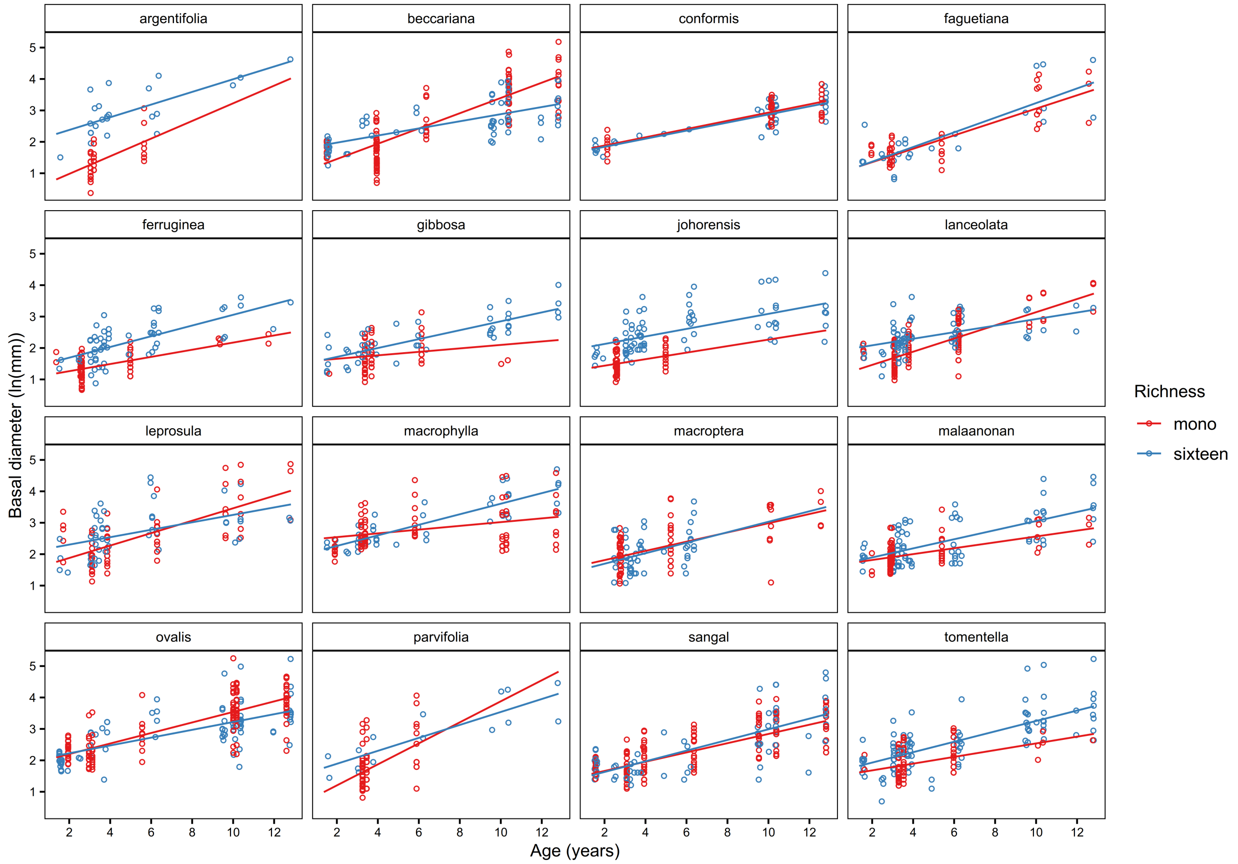

6.7 Relationships between seedlings and matrix variables

There is a clear positive relationship between canopy openness and survival, as well as total basal area, although extreme basal area values (large or small) may be skewing these initial plots. There is no clear effect on survival of the proportion of dipterocarps or the proportion of basal area from the largest tree, however.

```
seedlings %>%
  pivot_longer(cols = c(ba_total_log_scaled, prop_dip, prop_ba_max, openness_log),
               names_to = "Var",
               values_to = "Value") %>%
  ggplot(aes(Value, survival)) +
  facet_wrap(~ Var, scales = "free_x") +
  coord_cartesian(ylim = c(-0.05, 1.05)) +
  # geom_smooth() +
  stat_smooth(formula = "y ~ x", method = "glm", se = TRUE,
              method.args = list(family = "quasibinomial")) +
  geom_point(size = 0.5, alpha = 0.5)
```

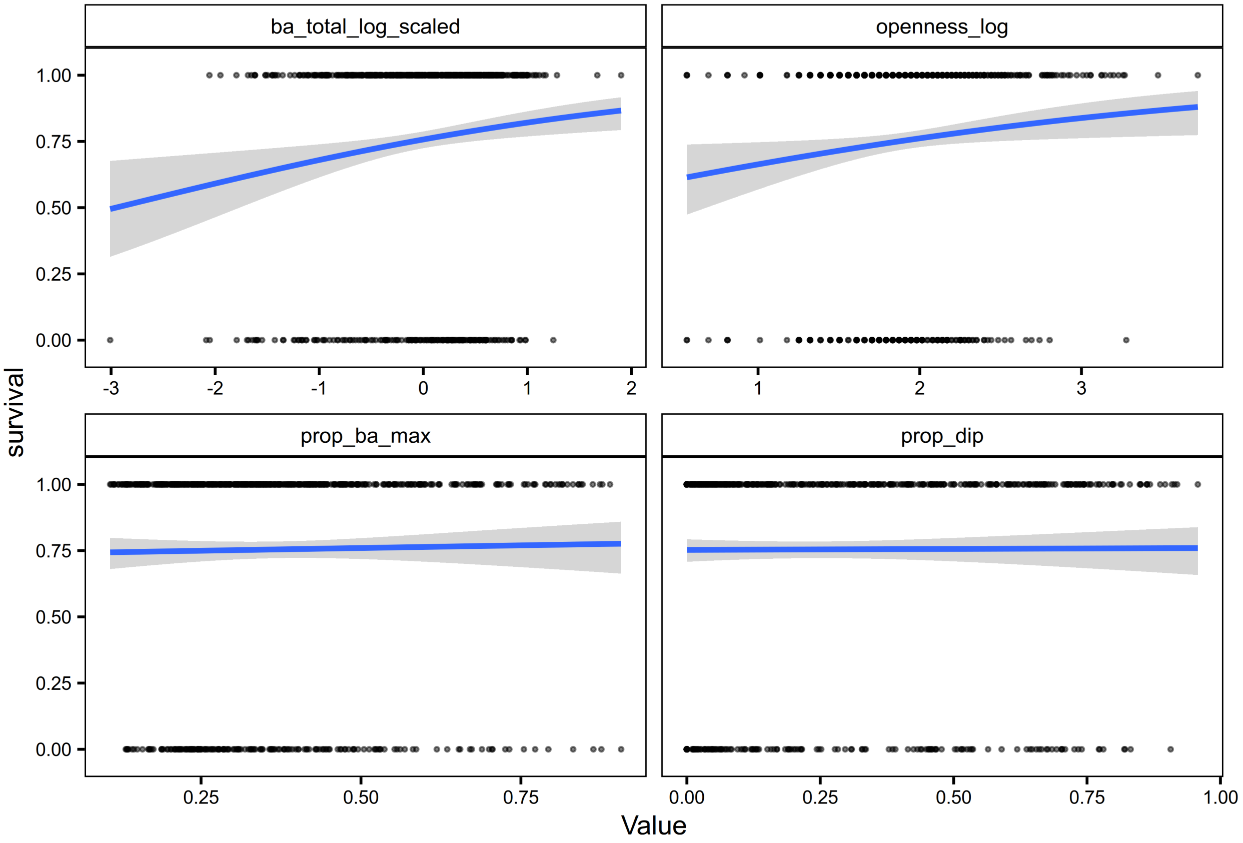

There is no immediately apparent relationship between RGR and basal area, the proportion surrounding basal area composed of the largest tree, or the proportion surrounding trees that are dipterocarps (although we have several plots where there are no dipterocarps present). We do, however, see an initial effect of canopy openness on RGR, but with much variation:

```
seedlings %>%
  pivot_longer(cols = c(ba_total_log_scaled, prop_dip, prop_ba_max, openness_log),
               names_to = "Var",
               values_to = "Value") %>%
  ggplot(aes(Value, rgr_2012)) +
  facet_wrap(~ Var, scales = "free_x") +
  # coord_cartesian(ylim = c(-0.05, 1.05)) +
  # geom_smooth() +
  stat_smooth(formula = "y ~ x", method = "glm", se = TRUE,
              method.args = list(family = "gaussian")) +
  geom_point(size = 0.5, alpha = 0.5)
```

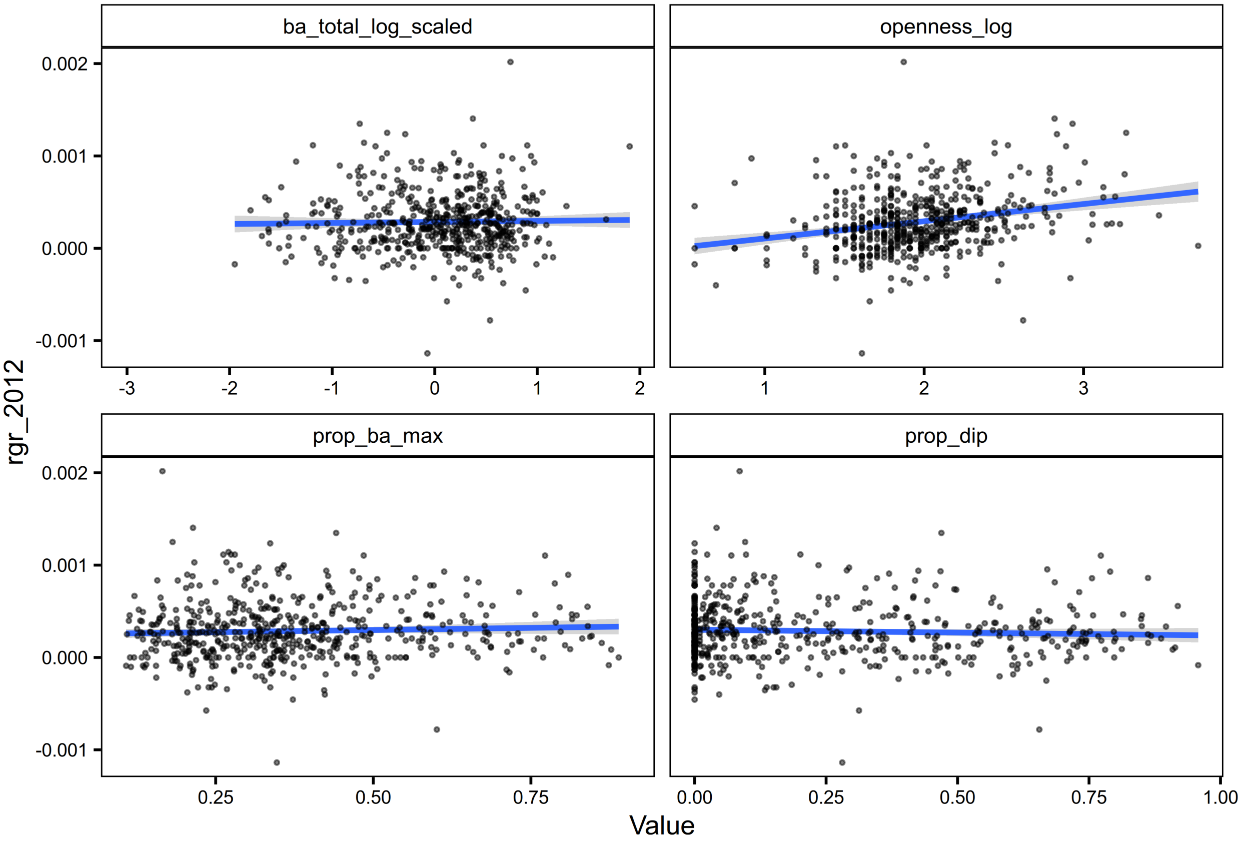

6.7.1 Dipterocarps and non-dipterocarps, and distances from focal tree

Data was collected specifying if surrounding matrix trees are dipterocarp or non-dipterocarp species and their relative locations to each planted tree (either under 5 m away or between 5 and 10 m away). We can plot if the relationship between RGR and the previously visualised variables is dependent on either of these additional parameters.

```
seedlings_total <- seedlings %>%
  dplyr::select(-ba_total_log, -ba_total_log_scaled) %>%
  pivot_longer(cols = starts_with(c("ba_total", "ba_mean", "dbh_max")),
               names_to = "type", values_to = "score") %>%
  dplyr::select(tree.id, IDPlots, rgr_2012, type, score, survival) %>%
  mutate(distance = case_when(grepl("5", type)  ~ "Within 5m",
                              grepl("10", type) ~ "Within 5-10m",
                              .default = "All trees")) %>%
  mutate(dip_nondip = case_when(grepl("nondip", type) ~ "Non-dipterocarps",
                                grepl("dip", type)    ~ "Dipterocarps",
                                .default = "All trees")) %>%
  filter(score > 0) %>%
  mutate(score_log = log(score))
```

There is no clear relationship with total basal area:

```
seedlings_total %>%
  filter(grepl("ba_total", type)) %>%
  ggplot(aes(log(score), rgr_2012)) +
  theme_doc +
```

```
  theme(axis.text.x = element_text(angle = 90, vjust = 0.5)) +
  facet_grid(distance ~ dip_nondip, scales = "free_x") +
  labs(y = expression(paste("RGR (mm ",mm^-1, " ",yr^-1,")")),
       x = "Log total basal area") +
  stat_smooth(formula = "y ~ x + I(x ^ 2)", method = "glm", se = TRUE,
              method.args = list(family = "gaussian")) +
  geom_point(size = 1, alpha = 0.5)
```

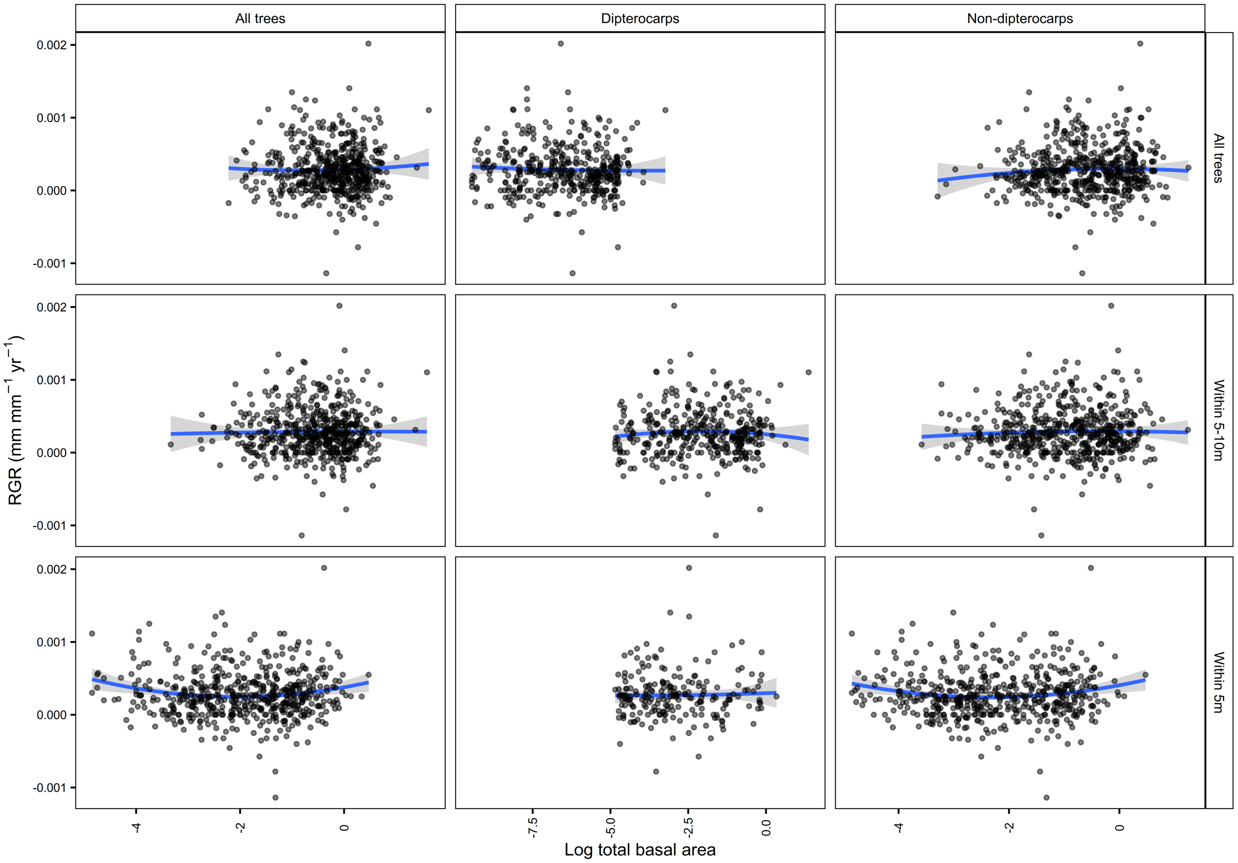

There is also no clear relationship with mean basal area:

```
seedlings_total %>%
  filter(grepl("ba_mean", type)) %>%
  ggplot(aes(log(score), rgr_2012)) +
  theme_doc +
  theme(axis.text.x = element_text(angle = 90, vjust = 0.5)) +
  facet_grid(distance ~ dip_nondip, scales = "free_x") +
  labs(y = expression(paste("RGR (mm ",mm^-1, " ",yr^-1,")")),
       x = "Log mean basal area") +
  stat_smooth(formula = "y ~ x + I(x ^ 2)", method = "glm", se = TRUE,
              method.args = list(family = "gaussian")) +
  geom_point(size = 1, alpha = 0.5)
```

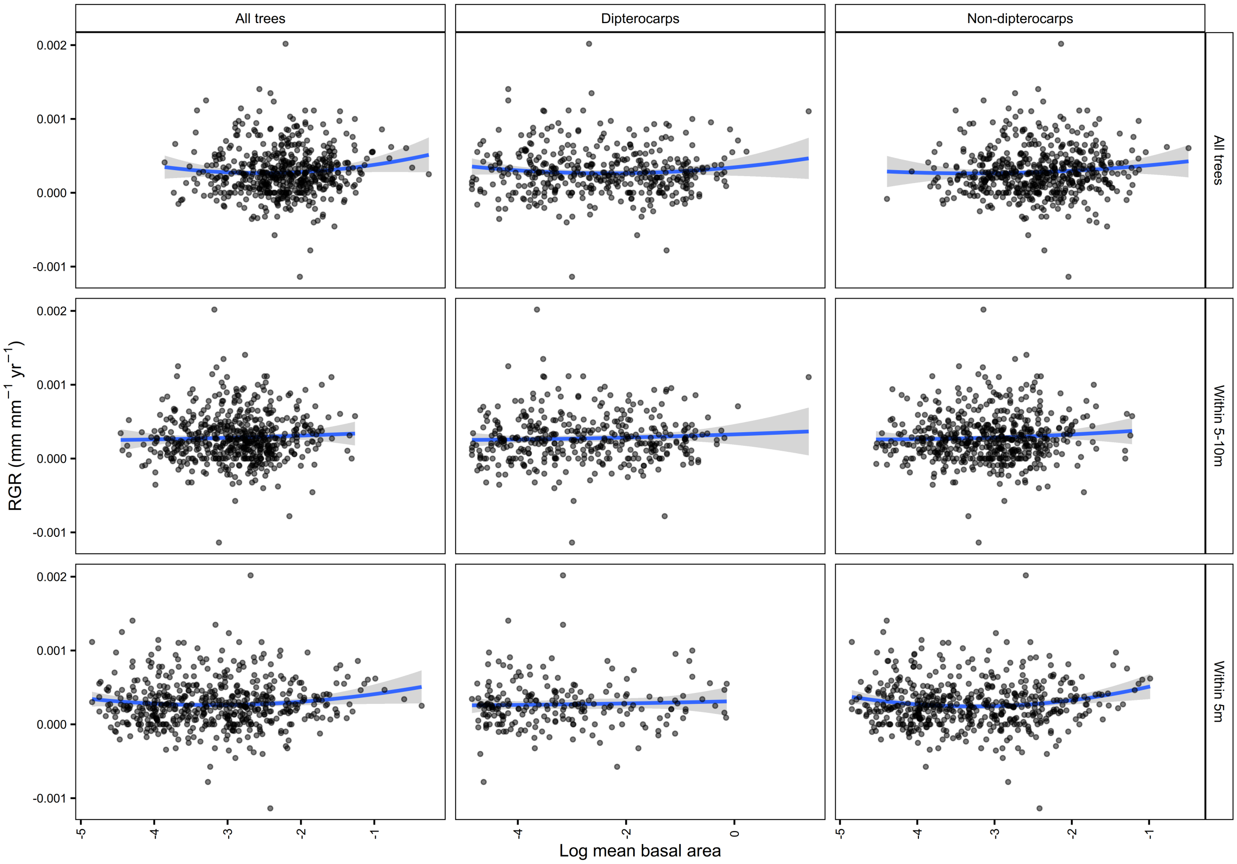

And there is also no clear relationship with DBH of largest tree:

```
seedlings_total %>%
  filter(grepl("dbh_max", type)) %>%
  ggplot(aes(log(score), rgr_2012)) +
  theme_doc +
  theme(axis.text.x = element_text(angle = 90, vjust = 0.5)) +
  facet_grid(distance ~ dip_nondip, scales = "free_x") +
  labs(y = expression(paste("RGR (mm ",mm^-1, " ",yr^-1,")")),
       x = "Log DBH largest tree") +
  stat_smooth(formula = "y ~ x + I(x ^ 2)", method = "glm", se = TRUE,
              method.args = list(family = "gaussian")) +
  geom_point(size = 1, alpha = 0.5)
```

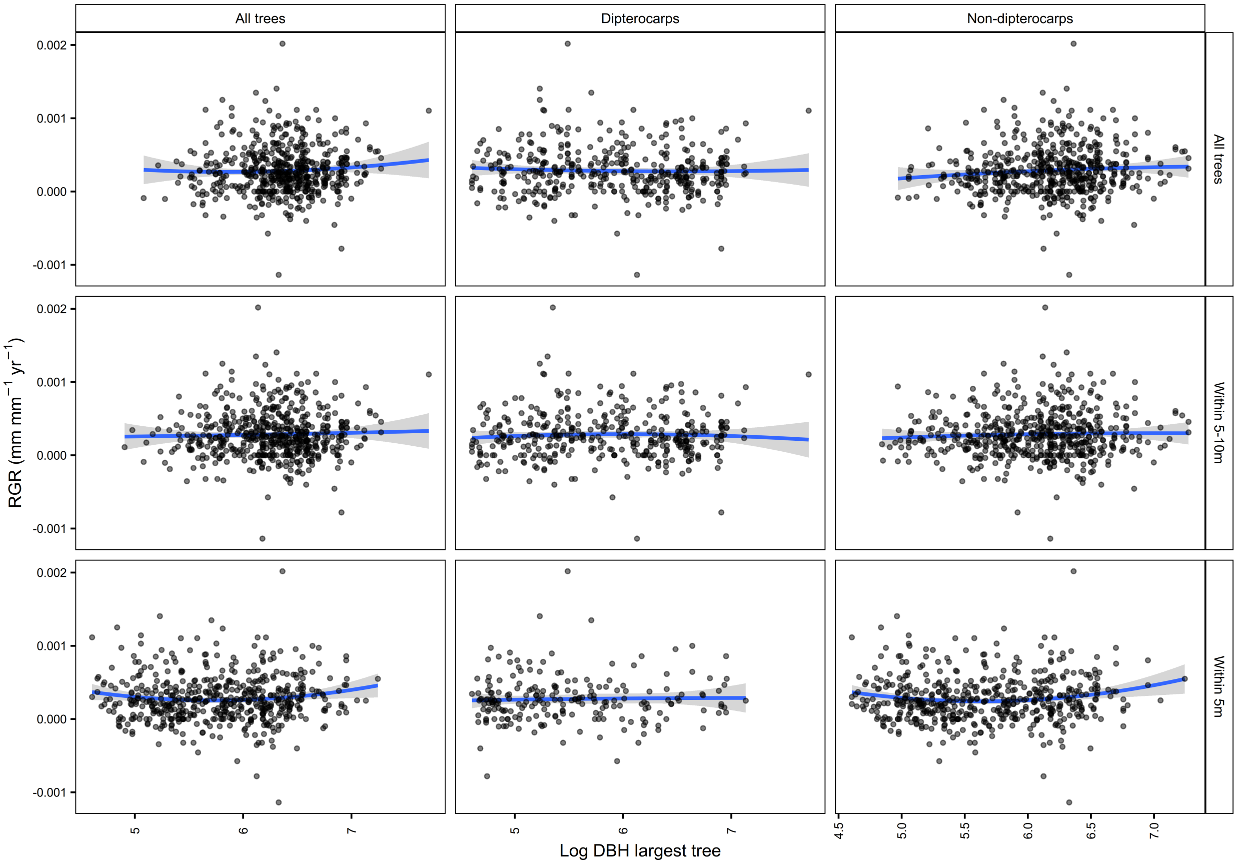

6.7.2 Survival and dipterocarp, non-dipterocarp, and distances

Repeating the same question asked concerning if the relationships between total basal area, mean basal area, and the size of the largest tree and survival varies for the distance of trees from the focal enrichment-planted seedling and the family of the tree (dipterocarps vs. non-dipterocarps).

There are potentially some positive relationships between survival and total non-dipterocarp, but not dipterocarp, basal area, but they are potentially due to some extreme values of basal area, particularly for very low scores.

```
seedlings_total %>%
  filter(grepl("ba_total", type)) %>%
  ggplot(aes(log(score), survival)) +
  theme_doc +
  theme(axis.text.x = element_text(angle = 90, vjust = 0.5)) +
  facet_grid(distance ~ dip_nondip, scales = "free_x") +
  labs(y = "Survival",
       x = "Log total basal area") +
  coord_cartesian(ylim = c(-0.05, 1.05)) +
  # geom_smooth() +
  stat_smooth(formula = "y ~ x + I(x ^ 2)", method = "glm", se = TRUE,
              method.args = list(family = "quasibinomial")) +
  geom_point(size = 1, alpha = 0.5)
```

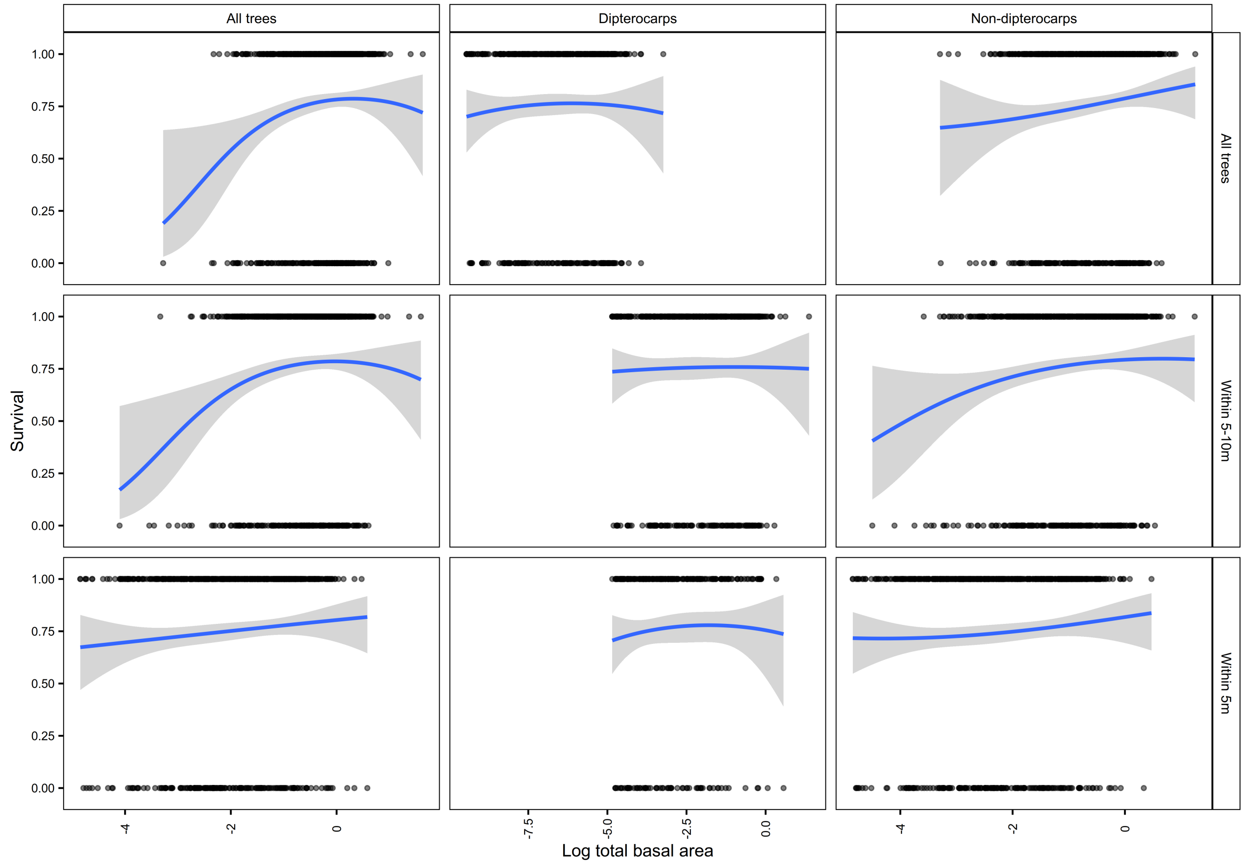

Similar to above, any differences appear due to individual values at tails, with the bulk of data not showing much difference between groups.

```
seedlings_total %>%
  filter(grepl("ba_mean", type)) %>%
  ggplot(aes(log(score), survival)) +
  theme_doc +
  theme(axis.text.x = element_text(angle = 90, vjust = 0.5)) +
  facet_grid(distance ~ dip_nondip, scales = "free_x") +
  labs(y = "Survival",
       x = "Log mean basal area") +
  coord_cartesian(ylim = c(-0.05, 1.05)) +
  # geom_smooth() +
  stat_smooth(formula = "y ~ x + I(x ^ 2)", method = "glm", se = TRUE,
              method.args = list(family = "quasibinomial")) +
  geom_point(size = 1, alpha = 0.5)
```

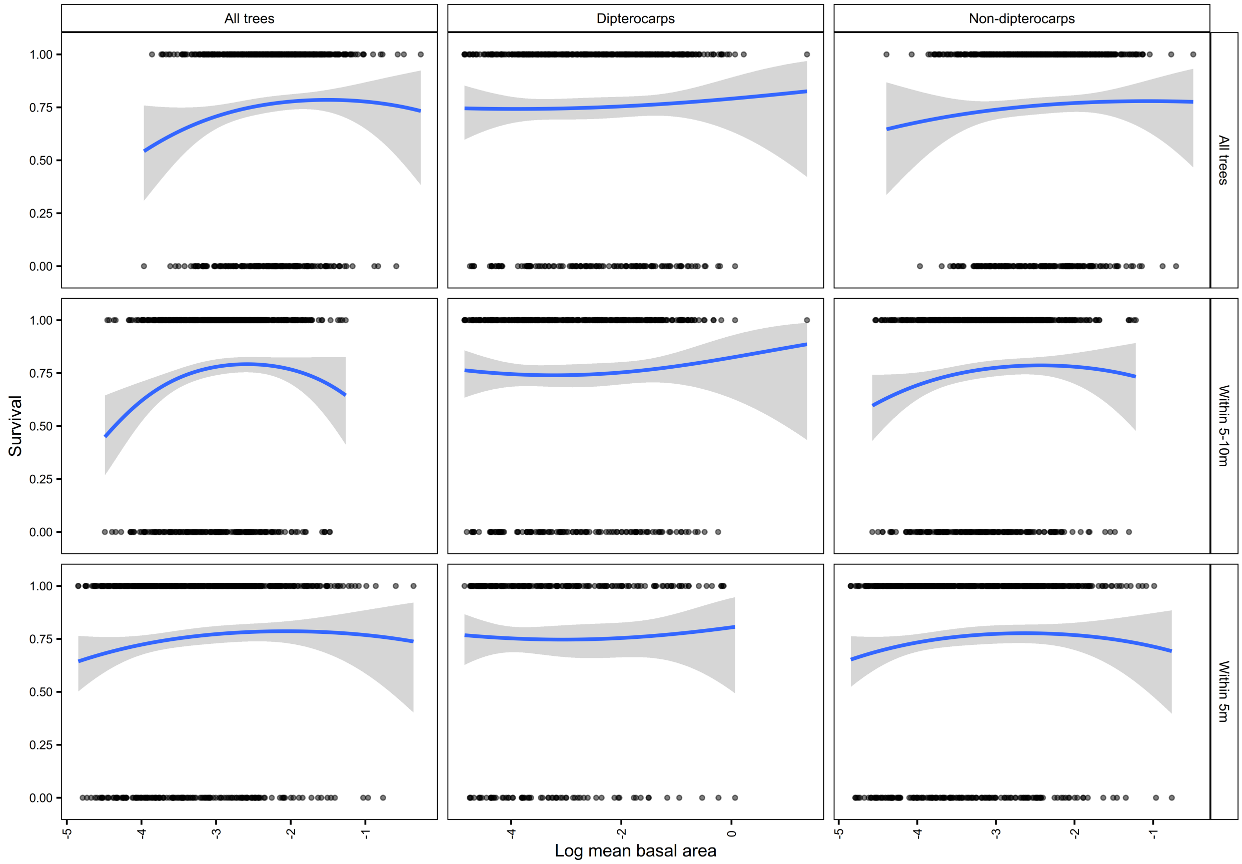

The same trend is seen across values, with differences largely due to extreme values at the tail ends rather than anything resulting from the bulk of our data.

```
seedlings_total %>%
  filter(grepl("dbh_max", type)) %>%
  ggplot(aes(log(score), survival)) +
  theme_doc +
  theme(axis.text.x = element_text(angle = 90, vjust = 0.5)) +
  facet_grid(distance ~ dip_nondip, scales = "free_x") +
  labs(y = "Survival",
       x = "Log DBH largest tree") +
  coord_cartesian(ylim = c(-0.05, 1.05)) +
  # geom_smooth() +
  stat_smooth(formula = "y ~ x + I(x ^ 2)", method = "glm", se = TRUE,
              method.args = list(family = "quasibinomial")) +
  geom_point(size = 1, alpha = 0.5)
```

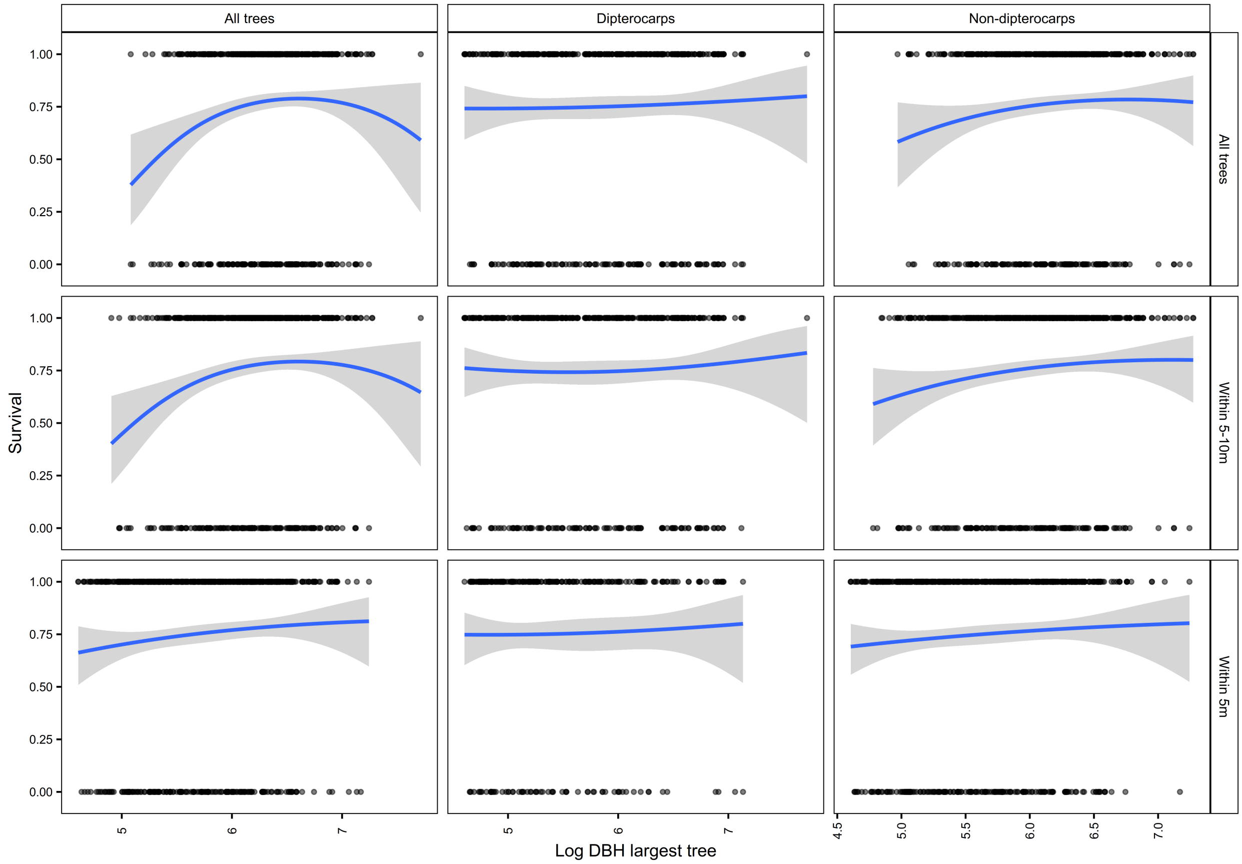

Correlations between the covariates (Figure A3). There is a correlation between proportion of dipterocarps and proportion of basal area of the largest tree, but only where the proportion of basal area as dipterocarps was very high.

```
### Fig. A3
fig_s3_data <- seedlings %>%
  dplyr::select(openness_log, ba_total_log, prop_dip, prop_ba_max) %>%
  rename("Log canopy openness"  = "openness_log",
         "Log total basal area" = "ba_total_log",
         "Prop. dipterocarps"   = "prop_dip",
         "Prop. largest tree"   = "prop_ba_max")

pairs(fig_s3_data,
      lower.panel = function(x, y) {
        par(usr = c(0, 1, 0, 1))
        text(0.5, 0.5, signif(cor(x, y), 3))},
      upper.panel = panel.smooth)
```

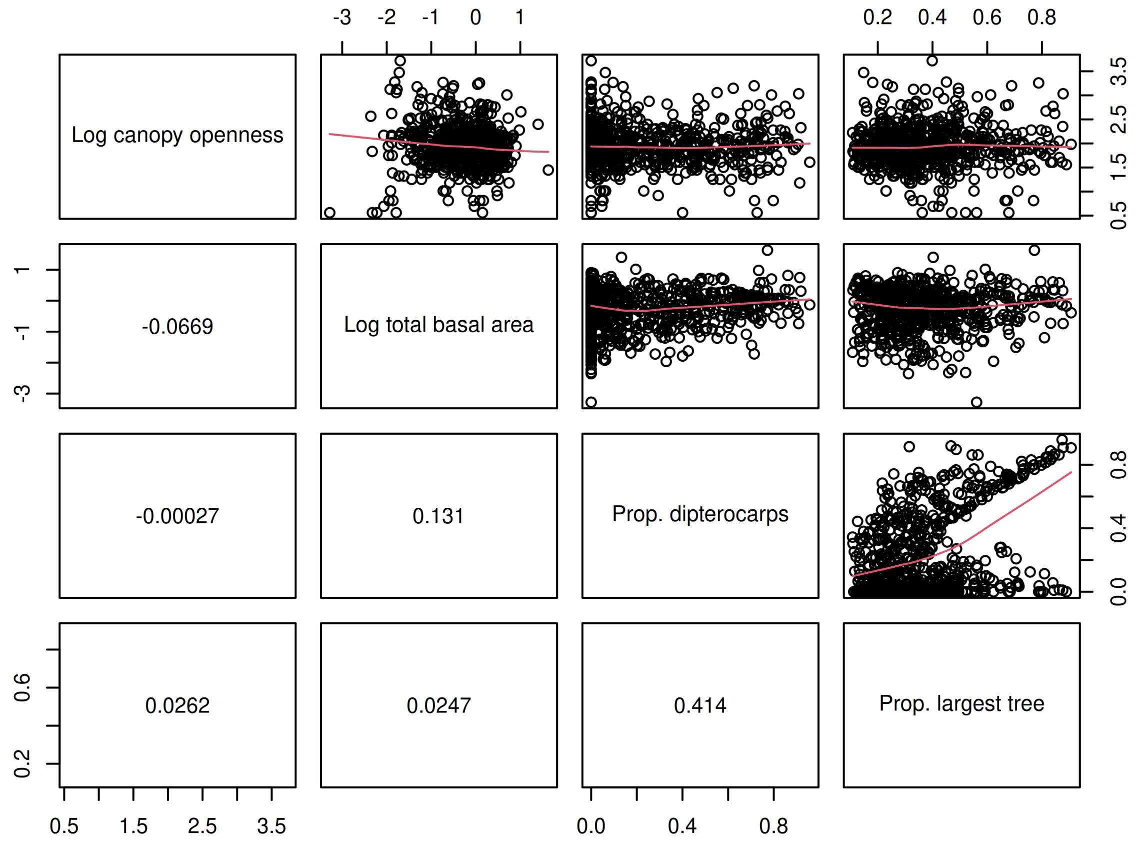


```r
### Save
png("Figures/Final/SI/fig_s3.png",
    width = 170, height = 130, units = "mm", bg = "white", res = 600)
pairs(fig_s3_data,
      lower.panel = function(x, y) {
        par(usr = c(0, 1, 0, 1))
        text(0.5, 0.5, signif(cor(x, y), 3))},
      upper.panel = panel.smooth)
dev.off()
```

```
## png
##   2
```

```r
pdf("Figures/Final/SI/fig_s3.pdf",
    width = 17 / 2.54, height = 13 / 2.54, bg = "white")
pairs(fig_s3_data,
      lower.panel = function(x, y) {
        par(usr = c(0, 1, 0, 1))
        text(0.5, 0.5, signif(cor(x, y), 3))},
      upper.panel = panel.smooth)
dev.off()
```

```
## png
##   2
```

7 Analysis

We use mixed effects models with plot and species as the random effects. Cohort and richness are added as covariates. We include canopy openness (logged) and total basal area (logged and centered). We also include the proportion of the total basal area that is made up of dipterocarps and the proportion of the total basal area that is made up of the largest tree. As we are also interested in a possible interaction between total basal area and both proportion dipterocarps and proportion maximum tree, the interaction terms are included.

7.1 Survival model fitting

Fitting our initial model:

```
mod1 <- glmer(survival ~ cohort + richness + openness_log + prop_dip + prop_ba_max +
                ba_total_log_scaled + ba_total_log_scaled:prop_dip +
                ba_total_log_scaled:prop_ba_max + (1|IDPlots) + (1 | species),
              control = glmerControl(optimizer = "bobyqa",
                                     optCtrl = list(maxfun = 1e+6)),
              data = seedlings, "binomial")
```

This will give us exact estimates for all our questions within a single model. Simplifying the model down using the Bayesian Information Criterion (BIC), as it is the most conservative method between itself and the Akaike Information Criterion (AIC).

```
mod2 <- update(mod1, .~. - prop_ba_max:ba_total_log_scaled)
anova(mod1,mod2) # can be dropped
```

```
## Data: seedlings
## Models:
## mod2: survival ~ cohort + richness + openness_log + prop_dip + prop_ba_max + ba_total_log_scaled + (
## mod1: survival ~ cohort + richness + openness_log + prop_dip + prop_ba_max + ba_total_log_scaled + ba
##      npar    AIC    BIC  logLik -2*log(L)  Chisq Df Pr(>Chisq)
## mod2   10 777.71 823.52 -378.86    757.71
## mod1   11 777.93 828.32 -377.96    755.93 1.7849  1     0.1815
```

```
mod3 <- update(mod2, .~. - prop_ba_max)
anova(mod3, mod2) # can be dropped
```

```
## Data: seedlings
## Models:
## mod3: survival ~ cohort + richness + openness_log + prop_dip + ba_total_log_scaled + (1 | IDPlots) +
## mod2: survival ~ cohort + richness + openness_log + prop_dip + prop_ba_max + ba_total_log_scaled + (
##      npar    AIC    BIC  logLik -2*log(L)  Chisq Df Pr(>Chisq)
## mod3    9 776.42 817.65 -379.21    758.42
## mod2   10 777.71 823.52 -378.86    757.71 0.7097  1     0.3995
```

```
mod4 <- update(mod3, .~. - richness)
anova(mod4, mod3) # can be dropped
```

```
## Data: seedlings
## Models:
## mod4: survival ~ cohort + openness_log + prop_dip + ba_total_log_scaled + (1 | IDPlots) + (1 | speci
## mod3: survival ~ cohort + richness + openness_log + prop_dip + ba_total_log_scaled + (1 | IDPlots) +
##      npar    AIC    BIC  logLik -2*log(L)  Chisq Df Pr(>Chisq)
## mod4    8 775.55 812.19 -379.77    759.55
## mod3    9 776.42 817.65 -379.21    758.42 1.1253  1     0.2888
```

```
mod5 <- update(mod4, .~. - prop_dip:ba_total_log_scaled)
anova(mod5, mod4) # can be dropped
```

```
## Data: seedlings
## Models:
## mod5: survival ~ cohort + openness_log + prop_dip + ba_total_log_scaled + (1 | IDPlots) + (1 | speci
## mod4: survival ~ cohort + openness_log + prop_dip + ba_total_log_scaled + (1 | IDPlots) + (1 | speci
##      npar    AIC    BIC  logLik -2*log(L) Chisq Df Pr(>Chisq)
## mod5    7 777.21 809.28 -381.61    763.21
## mod4    8 775.55 812.19 -379.77    759.55 3.663  1    0.05563 .
## ---
## Signif. codes:  0 '***' 0.001 '**' 0.01 '*' 0.05 '.' 0.1 ' ' 1
```

```r
mod5 <- update(mod4, .~. - prop_dip:ba_total_log_scaled)
anova(mod5, mod4) # can be dropped
```

```
## Data: seedlings
## Models:
## mod5: survival ~ cohort + openness_log + prop_dip + ba_total_log_scaled + (1 | IDPlots) + (1 | speci
## mod4: survival ~ cohort + openness_log + prop_dip + ba_total_log_scaled + (1 | IDPlots) + (1 | speci
##      npar    AIC    BIC  logLik -2*log(L) Chisq Df Pr(>Chisq)
## mod5    7 777.21 809.28 -381.61    763.21
## mod4    8 775.55 812.19 -379.77    759.55 3.663  1    0.05563 .
## ---
## Signif. codes:  0 '***' 0.001 '**' 0.01 '*' 0.05 '.' 0.1 ' ' 1
```

```r
mod6 <- update(mod5, .~. - prop_dip)
anova(mod6, mod5) # can be dropped
```

```
## Data: seedlings
## Models:
## mod6: survival ~ cohort + openness_log + ba_total_log_scaled + (1 | IDPlots) + (1 | species)
## mod5: survival ~ cohort + openness_log + prop_dip + ba_total_log_scaled + (1 | IDPlots) + (1 | speci
##      npar    AIC    BIC  logLik -2*log(L)  Chisq Df Pr(>Chisq)
## mod6    6 775.53 803.01 -381.76    763.53
## mod5    7 777.21 809.28 -381.61    763.21 0.3152  1     0.5745
```

```r
mod7 <- update(mod5, .~. - ba_total_log_scaled)
anova(mod7, mod6) # cannot be dropped
```

```
## Data: seedlings
## Models:
## mod7: survival ~ cohort + openness_log + prop_dip + (1 | IDPlots) + (1 | species)
## mod6: survival ~ cohort + openness_log + ba_total_log_scaled + (1 | IDPlots) + (1 | species)
##      npar    AIC    BIC  logLik -2*log(L)  Chisq Df Pr(>Chisq)
## mod7    6 784.08 811.57 -386.04    772.08
## mod6    6 775.53 803.01 -381.76    763.53 8.5535  0
```

Extracting estimated survival effect of prop_ba_max

```r
summary(mod2)$coefficients[6,1]
```

```
## [1] 0.5198631
```

Extracting estimated survival effect of prop_dip

```r
summary(mod5)$coefficients[4,1]
```

```
## [1] -0.2059566
```

```r
summary(mod5)$coefficients[4,1] - 1.96*summary(mod5)$coefficients[4,2] # lower interval
```

```
## [1] -0.922428
```

```r
summary(mod5)$coefficients[4,1] + 1.96*summary(mod5)$coefficients[4,2] # upper interval
```

```
## [1] 0.5105147
```

So our final survival model is:

```r
survival_mod <- glmer(survival ~ cohort + openness_log + ba_total_log_scaled +
                        (1|IDPlots) + (1|species),
                      control = glmerControl(optimizer = "bobyqa",
                                             optCtrl = list(maxfun = 1e+1)),
                      data = seedlings, "binomial")
```

7.1.1 Survival model inspection

```r
summary(survival_mod)
```

```
## Generalized linear mixed model fit by maximum likelihood (Laplace
##   Approximation) [glmerMod]
##  Family: binomial  ( logit )
## Formula: survival ~ cohort + openness_log + ba_total_log_scaled + (1 |
##     IDPlots) + (1 | species)
##    Data: seedlings
## Control: glmerControl(optimizer = "bobyqa", optCtrl = list(maxfun = 10))
##
##       AIC       BIC    logLik -2*log(L)  df.resid
##     778.3     805.8    -383.1     766.3       715
##
## Scaled residuals:
##     Min      1Q  Median      3Q     Max
## -3.4453  0.2522  0.4582  0.5946  1.3494
##
## Random effects:
##  Groups  Name        Variance Std.Dev.
##  IDPlots (Intercept) 0.1335   0.3654
##  species (Intercept) 0.1794   0.4235
## Number of obs: 721, groups:  IDPlots, 24; species, 16
##
## Fixed effects:
##                     Estimate Std. Error z value Pr(>|z|)
## (Intercept)           0.9832     0.5155   1.907 0.056486 .
## cohort2              -0.9046     0.2500  -3.619 0.000296 ***
## openness_log          0.4532     0.2347   1.931 0.053446 .
## ba_total_log_scaled   0.4018     0.1429   2.811 0.004935 **
## ---
## Signif. codes:  0 '***' 0.001 '**' 0.01 '*' 0.05 '.' 0.1 ' ' 1
##
## Correlation of Fixed Effects:
##             (Intr) cohrt2 opnns_
## cohort2     -0.382
## openness_lg -0.864 -0.013
## b_ttl_lg_sc -0.031  0.052  0.031
```

```
## optimizer (bobyqa) convergence code: 1 (bobyqa -- maximum number of function evaluations exceeded)
## Model failed to converge with max|grad| = 2.73845 (tol = 0.002, component 1)
## maxfun < 10 * length(par)^2 is not recommended.
```

The model r squared:

```r
r.squaredGLMM(survival_mod)
```

```
##                   R2m       R2c
## theoretical 0.0727327 0.1532567
## delta       0.0486236 0.1024559
```

We do not have any serious overdispersion:

```r
dispersion_glmer(survival_mod)
```

```
## [1] 1.01518
```

Our random effects do not explain much variation. Specifically, `IDPlots` explains little and `species` explains nothing. The latter is the cause of the singular fit in our model.

```r
mean(ranef(survival_mod)$IDPlots[, 1])
```

```
## [1] -9.940558e-05
```

```r
mean(ranef(survival_mod)$species[, 1])
```

```
## [1] -0.0002003156
```

Our residual plots look good:

```r
par(mfrow = c(1, 2))
binnedplot(fitted(survival_mod), resid(survival_mod))
halfnorm(residuals(survival_mod))
```

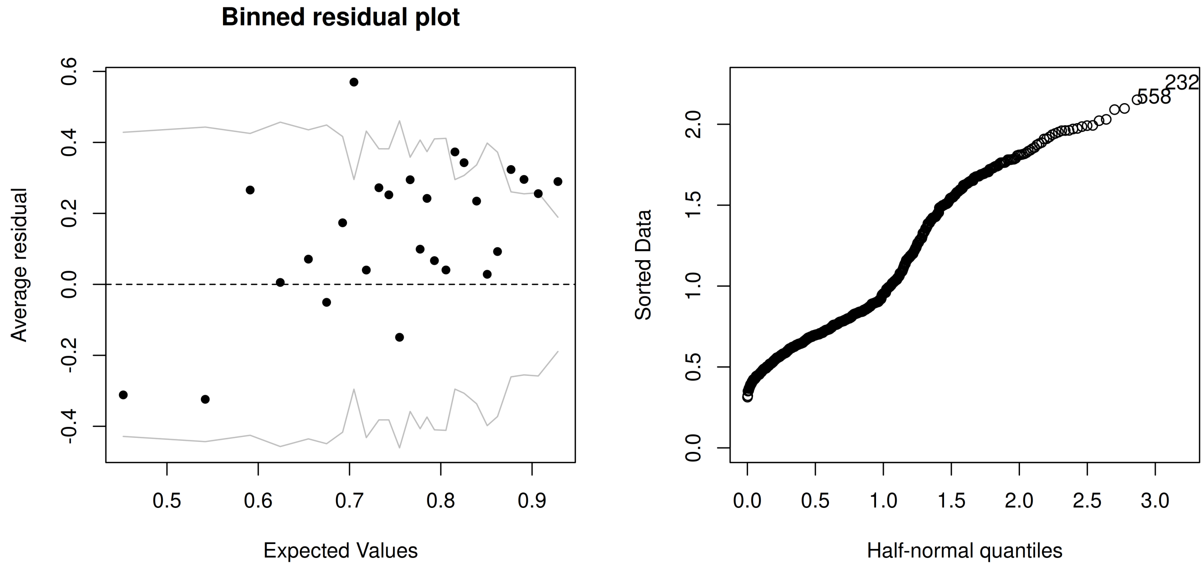

Residuals of random effects:

```r
par(mfrow = c(1, 2))
p1 <- qqmath(~ranef(survival_mod)$IDPlots[, 1]  ,
             distribution = qnorm, prepanel = prepanel.qqmathline,
```

```
             panel = function(x, ...) {
               panel.qqmathline(x, ...)
               panel.qqmath(x, ...)
             }, main = "QQ of IDPlots",
             xlab = "Theoretical quantiles", ylab = "Standardised residuals")
p2 <- qqmath(~ranef(survival_mod)$species[, 1]  ,
             distribution = qnorm, prepanel = prepanel.qqmathline,
             panel = function(x, ...) {
               panel.qqmathline(x, ...)
               panel.qqmath(x, ...)
             }, main = "QQ of species",
             xlab = "Theoretical quantiles", ylab = "Standardised residuals")
grid.arrange(p1, p2, ncol = 2)
```

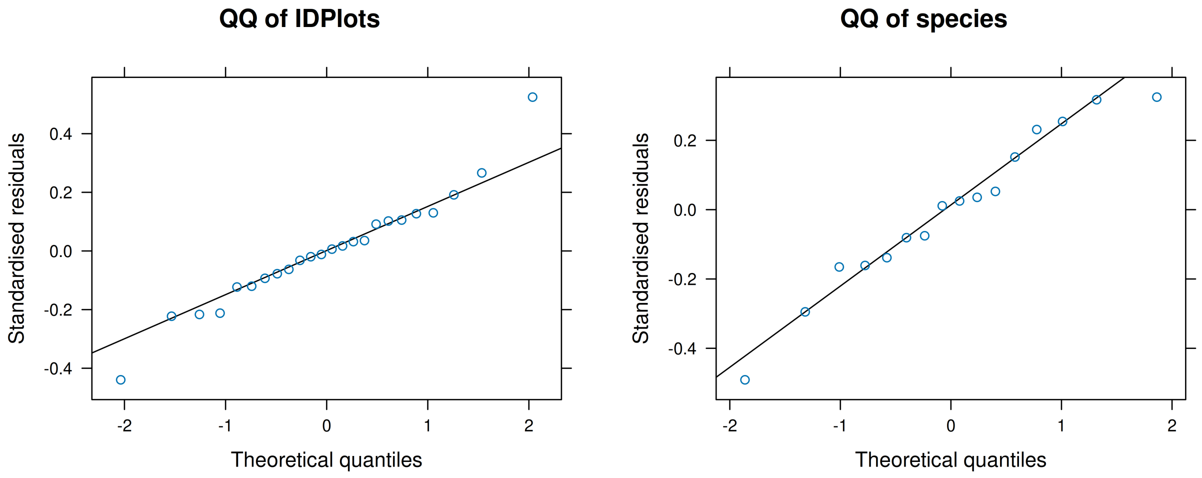

Residuals across both random effects

```
survival_mod_augmented <- augment(survival_mod) %>%
  left_join(dplyr::select(seedlings, genus, species), by = "species") %>%
  mutate(sciname = paste(strtrim(genus, 1), species, sep = ". "))

p1 <- ggplot(survival_mod_augmented, aes(IDPlots, .resid)) +
  theme_doc +
  coord_flip() +
  labs(y = "Residual", x = "Plot ID") +
  geom_boxplot()

p2 <- ggplot(survival_mod_augmented, aes(sciname, .resid)) +
  theme_doc +
  theme(axis.text.y = element_text(face = "italic")) +
  coord_flip() +
  labs(y = "Residual", x = "Species") +
  geom_boxplot()

grid.arrange(p1, p2, ncol = 2)
```

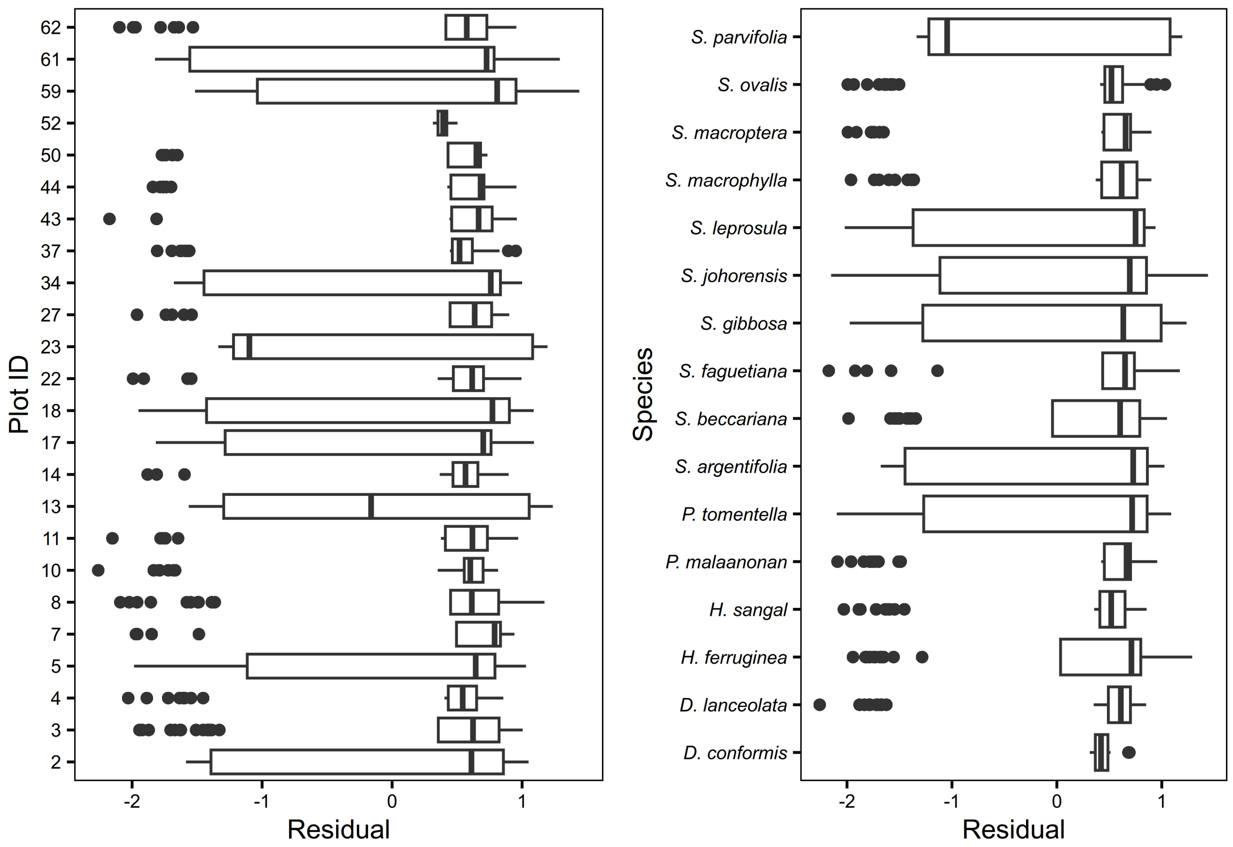


```
### Save species residuals for saving as fig. S5 later
fig_s5a <- p2
```

7.2 Growth model fitting

We create a subset of the `seedlings` dataset, as we only want the trees that survived to 2015 and so will give non-NA RGR values:

```
growth <- seedlings %>%
  filter(survival == 1) %>%
  mutate(rgr = rgr_2012 * 365.25) %>% # so now rgr is expressed in years
  filter(!is.na(rgr))
```

Our initial main model is:

```
mod1 <- lmer(rgr ~ cohort + richness + openness_log + prop_dip + prop_ba_max +
               ba_total_log_scaled + ba_total_log_scaled:prop_dip +
               ba_total_log_scaled:prop_ba_max + (1|IDPlots) + (1|species),
             data = growth)
```

Again, we see an effect of cohort, openness, and now also proportion dipterocarps. Removing terms one at a time until the model is no longer improved:

```
mod2 <- update(mod1, .~. - prop_ba_max:ba_total_log_scaled)
anova(mod2, mod1) # can be dropped
```

```
## Data: growth
## Models:
```

```
## mod2: rgr ~ cohort + richness + openness_log + prop_dip + prop_ba_max + ba_total_log_scaled + (1 | II
## mod1: rgr ~ cohort + richness + openness_log + prop_dip + prop_ba_max + ba_total_log_scaled + ba_tota
##      npar     AIC     BIC logLik -2*log(L) Chisq Df Pr(>Chisq)
## mod2   11 -842.89 -795.68 432.44   -864.89
## mod1   12 -840.90 -789.40 432.45   -864.90 0.008  1     0.9286
```

```
mod3 <- update(mod2, .~. - prop_dip:ba_total_log_scaled)
anova(mod3, mod2) # can be dropped
```

```
## Data: growth
## Models:
## mod3: rgr ~ cohort + richness + openness_log + prop_dip + prop_ba_max + ba_total_log_scaled + (1 | II
## mod2: rgr ~ cohort + richness + openness_log + prop_dip + prop_ba_max + ba_total_log_scaled + (1 | II
##      npar     AIC     BIC logLik -2*log(L) Chisq Df Pr(>Chisq)
## mod3   10 -843.99 -801.07 431.99   -863.99
## mod2   11 -842.89 -795.68 432.44   -864.89   0.9  1     0.3428
```

```
mod4 <- update(mod3, .~. - ba_total_log_scaled)
anova(mod4, mod3) # can be dropped
```

```
## Data: growth
## Models:
## mod4: rgr ~ cohort + richness + openness_log + prop_dip + prop_ba_max + (1 | IDPlots) + (1 | species)
## mod3: rgr ~ cohort + richness + openness_log + prop_dip + prop_ba_max + ba_total_log_scaled + (1 | II
##      npar     AIC     BIC logLik -2*log(L)  Chisq Df Pr(>Chisq)
## mod4    9 -844.09 -805.47 431.05   -862.09
## mod3   10 -843.99 -801.07 431.99   -863.99 1.8966  1     0.1685
```

```
mod5 <- update(mod4, .~. - prop_ba_max)
anova(mod5, mod4) # can be dropped
```

```
## Data: growth
## Models:
## mod5: rgr ~ cohort + richness + openness_log + prop_dip + (1 | IDPlots) + (1 | species)
## mod4: rgr ~ cohort + richness + openness_log + prop_dip + prop_ba_max + (1 | IDPlots) + (1 | species)
##      npar     AIC     BIC logLik -2*log(L)  Chisq Df Pr(>Chisq)
## mod5    8 -843.52 -809.19 429.76   -859.52
## mod4    9 -844.09 -805.47 431.05   -862.09 2.5694  1     0.1089
```

```
mod6 <- update(mod5, .~. - prop_dip)
anova(mod6, mod5) # can be dropped
```

```
## Data: growth
## Models:
## mod6: rgr ~ cohort + richness + openness_log + (1 | IDPlots) + (1 | species)
## mod5: rgr ~ cohort + richness + openness_log + prop_dip + (1 | IDPlots) + (1 | species)
##      npar     AIC     BIC logLik -2*log(L)  Chisq Df Pr(>Chisq)
## mod6    7 -842.42 -812.37 428.21   -856.42
## mod5    8 -843.52 -809.19 429.76   -859.52 3.1062  1      0.078 .
## ---
## Signif. codes:  0 '***' 0.001 '**' 0.01 '*' 0.05 '.' 0.1 ' ' 1
```

```
mod7 <- update(mod6, .~. - richness)
anova(mod7, mod6) # can be dropped
```

```
## Data: growth
## Models:
## mod7: rgr ~ cohort + openness_log + (1 | IDPlots) + (1 | species)
```

```
## mod6: rgr ~ cohort + richness + openness_log + (1 | IDPlots) + (1 | species)
##      npar     AIC     BIC logLik -2*log(L)  Chisq Df Pr(>Chisq)
## mod7    6 -842.31 -816.56 427.16   -854.31
## mod6    7 -842.42 -812.37 428.21   -856.42 2.1029  1      0.147
```

```
mod8 <- update(mod6, .~. - openness_log)
anova(mod8, mod7) # cannot be dropped
```

```
## Data: growth
## Models:
## mod8: rgr ~ cohort + richness + (1 | IDPlots) + (1 | species)
## mod7: rgr ~ cohort + openness_log + (1 | IDPlots) + (1 | species)
##      npar     AIC     BIC logLik -2*log(L)  Chisq Df Pr(>Chisq)
## mod8    6 -812.67 -786.92 412.33   -824.67
## mod7    6 -842.31 -816.56 427.16   -854.31 29.647  0
```

Extracting total basal area effects. Since we have centered values that doesn't change the impact of a unit increase, so we can take values directly from the resulting tables:

```
summary(mod3)$coefficients[7, 1]
```

```
## [1] 0.0112775
```

```
confint(mod3)[10, 1:2]
```

```
##        2.5 %       97.5 %
## -0.004895079  0.027892630
```

Extracting values for prop_ba_max

```
summary(mod4)$coefficients[6, 1]
```

```
## [1] 0.0515422
```

```
confint(mod4)[9, 1:2]
```

```
##       2.5 %      97.5 %
## -0.01135944  0.11309916
```

Extracting values for prop_dips

```
summary(mod5)$coefficients[5, 1]
```

```
## [1] -0.03288467
```

```
confint(mod5)[8, 1:2]
```

```
##        2.5 %       97.5 %
## -0.069734256  0.003712823
```

Our final model is relatively simple, only maintaining the effect of cohort and canopy openness.

```
growth_mod <- lmer(rgr ~ cohort + openness_log +
                     (1 | IDPlots) + (1 | species),
                   data = growth)
```

7.2.1 Growth model inspection

```
summary(growth_mod)
```

```
## Linear mixed model fit by REML ['lmerMod']
## Formula: rgr ~ cohort + openness_log + (1 | IDPlots) + (1 | species)
```

```
##    Data: growth
##
## REML criterion at convergence: -832.6
##
## Scaled residuals:
##     Min      1Q  Median      3Q     Max
## -4.6432 -0.5497 -0.0947  0.5183  5.4830
##
## Random effects:
##  Groups   Name        Variance  Std.Dev.
##  IDPlots  (Intercept) 0.0013010 0.03607
##  species  (Intercept) 0.0002742 0.01656
##  Residual             0.0113352 0.10647
## Number of obs: 540, groups:  IDPlots, 24; species, 16
##
## Fixed effects:
##              Estimate Std. Error t value
## (Intercept)  -0.05718    0.02590  -2.207
## cohort2       0.04096    0.01094   3.745
## openness_log  0.06772    0.01178   5.750
##
## Correlation of Fixed Effects:
##             (Intr) cohrt2
## cohort2     -0.263
## openness_lg -0.877 -0.038
```

The model r squared:

```r
r.squaredGLMM(growth_mod)
```

```
##             R2m       R2c
## [1,] 0.08451883 0.1962173
```

Residual plots, separated by cohort:

```r
p1 <- xyplot(resid(growth_mod, type = "pearson")[growth$cohort == 1] ~
               fitted(growth_mod)[growth$cohort == 1],
             type = c("p", "smooth", "g"), col.line = "black",
             main = "Tukey-Anscombe - Cohort 1",
             xlab = "Fitted values", ylab = "Residuals")
p2 <- xyplot(resid(growth_mod, type = "pearson")[growth$cohort == 2] ~
               fitted(growth_mod)[growth$cohort == 2],
             type = c("p", "smooth", "g"), col.line = "black",
             main = "Tukey-Anscombe - Cohort 2",
             xlab = "Fitted values", ylab = "Residuals")
p3 <- xyplot(sqrt(abs(resid(growth_mod)))[growth$cohort == 1] ~
               fitted(growth_mod)[growth$cohort == 1],
             type = c("p", "smooth", "g"), col.line = "black",
             main = "Scale location - Cohort 1",
             xlab = "Fitted values", ylab = "sqrt(|Standardised Residuals|)")
p4 <- xyplot(sqrt(abs(resid(growth_mod)))[growth$cohort == 2] ~
               fitted(growth_mod)[growth$cohort == 2],
             type = c("p", "smooth", "g"), col.line = "black",
             main = "Scale location - Cohort 2",
             xlab = "Fitted values", ylab = "sqrt(|Standardised Residuals|)")
grid.arrange(p1, p2, p3, p4, ncol = 2)
```

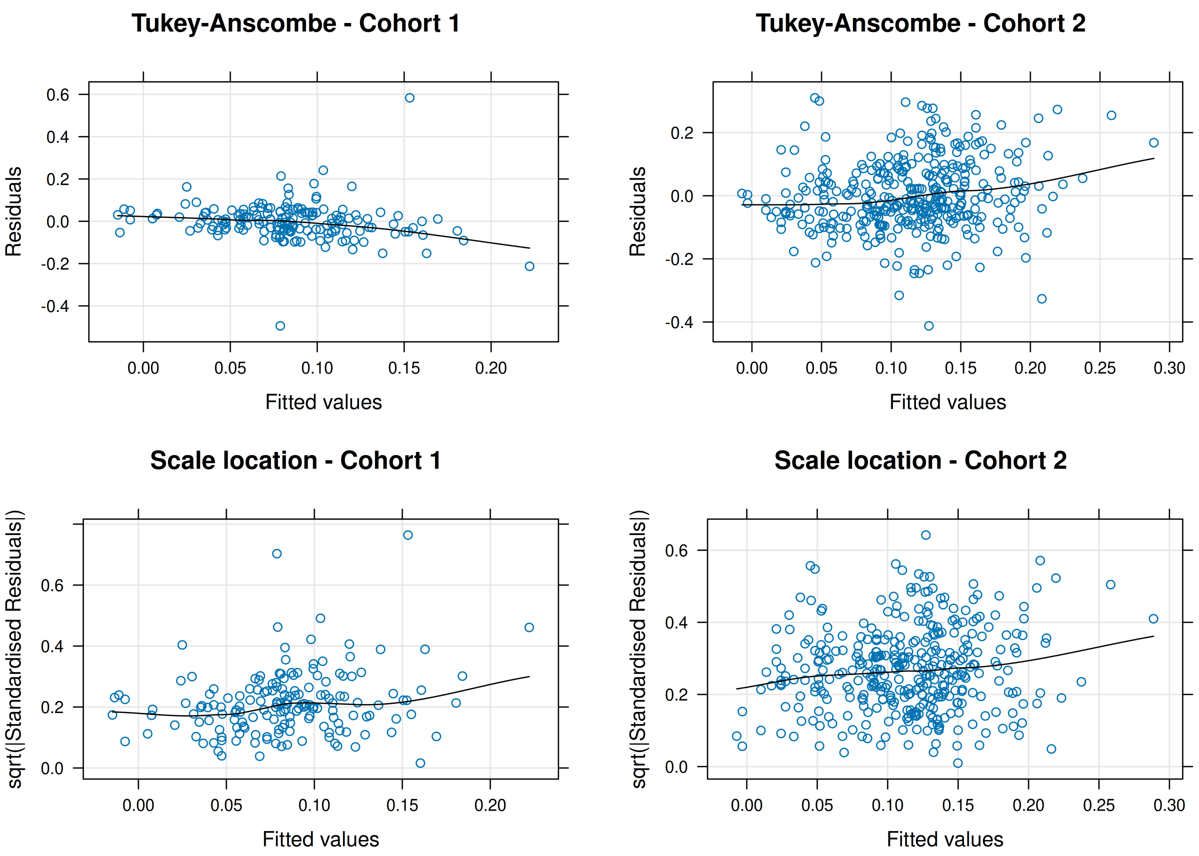

QQ plots for cohort as well as our random parameters:

```
p1 <- qqmath(~resid(growth_mod, type = "pearson")[growth$cohort == 1],
             distribution = qnorm,
             prepanel = prepanel.qqmathline,
             panel = function(x, ...) {
               panel.qqmathline(x, ...)
               panel.qqmath(x, ...)
             }, main = "QQ of residuals - Cohort 1",
             xlab = "Theoretical quantiles", ylab = "Standardised residuals")
p2 <- qqmath(~resid(growth_mod, type = "pearson")[growth$cohort == 2],
             distribution = qnorm,
             prepanel = prepanel.qqmathline,
             panel = function(x, ...) {
               panel.qqmathline(x, ...)
               panel.qqmath(x, ...)
             }, main = "QQ of residuals - Cohort 2",
             xlab = "Theoretical quantiles", ylab = "Standardised residuals")
p3 <- qqmath(~ranef(growth_mod)$IDPlots[, 1]   ,
             distribution = qnorm,
             prepanel = prepanel.qqmathline,
             panel = function(x, ...) {
               panel.qqmathline(x, ...)
               panel.qqmath(x, ...)
             }, main = "QQ of IDPlots",
             xlab = "Theoretical quantiles", ylab = "Standardised residuals")
```

```
p4 <- qqmath(~ranef(growth_mod)$species[, 1]   ,
             distribution = qnorm,
             prepanel = prepanel.qqmathline,
             panel = function(x, ...) {
               panel.qqmathline(x, ...)
               panel.qqmath(x, ...)
             }, main = "QQ of species",
             xlab = "Theoretical quantiles", ylab = "Standardised residuals")
grid.arrange(p1, p2, p3, p4, ncol = 2)
```

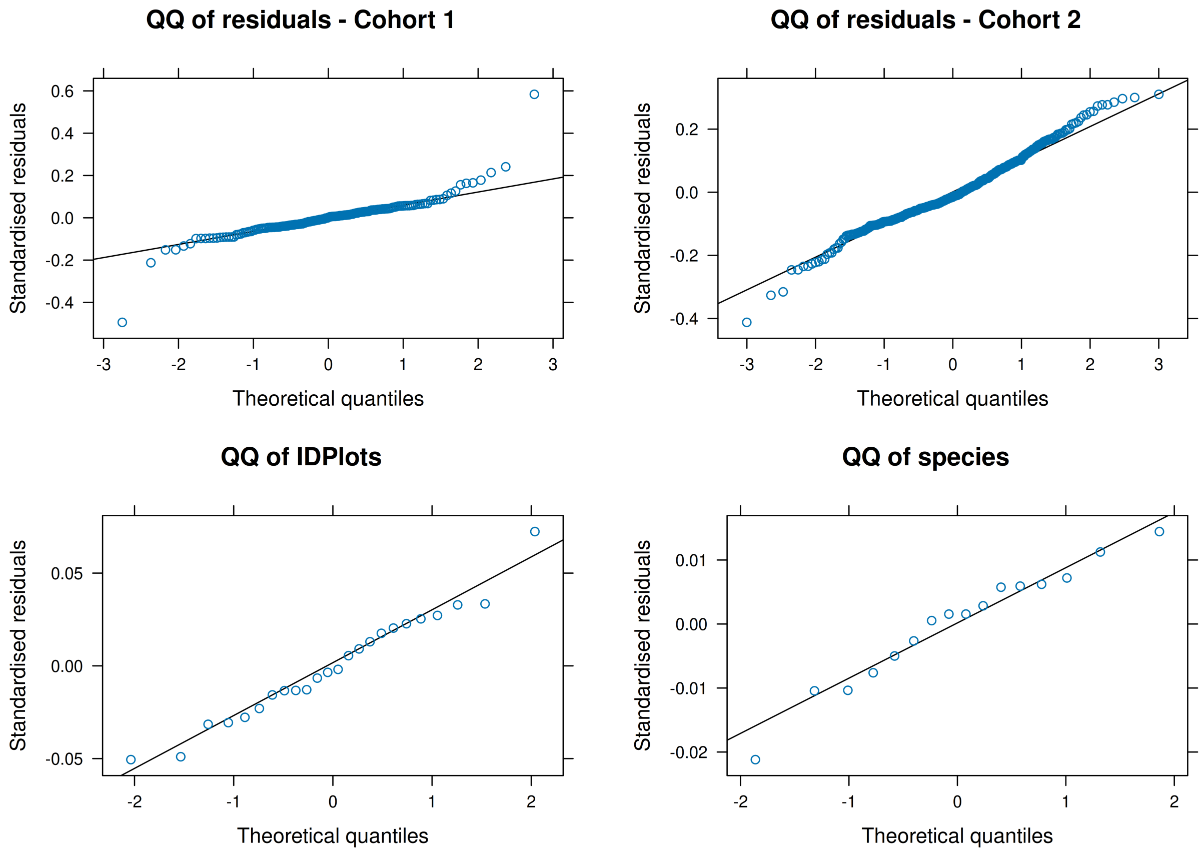

Like our survival model, the variance explained by random effects is very small:

```
mean(ranef(growth_mod)$IDPlots[, 1])
```

```
## [1] -1.172205e-16
```

```
mean(ranef(growth_mod)$species[, 1])
```

```
## [1] -3.546696e-17
```

Residuals of the random effects:

```
growth_mod_augmented <- augment(growth_mod) %>%
  left_join(dplyr::select(seedlings, genus, species), by = "species") %>%
  mutate(sciname = paste(strtrim(genus, 1), species, sep = ". "))

p1 <- ggplot(growth_mod_augmented, aes(IDPlots, .resid)) +
  theme_doc +
```

```r
  coord_flip() +
  labs(y = "Residual", x = "Plot ID") +
  geom_boxplot()

p2 <- ggplot(growth_mod_augmented, aes(sciname, .resid)) +
  theme_doc +
  theme(axis.text.y = element_text(face = "italic")) +
  coord_flip() +
  labs(y = "Residual", x = "Species") +
  geom_boxplot()

grid.arrange(p1, p2, ncol = 2)
```

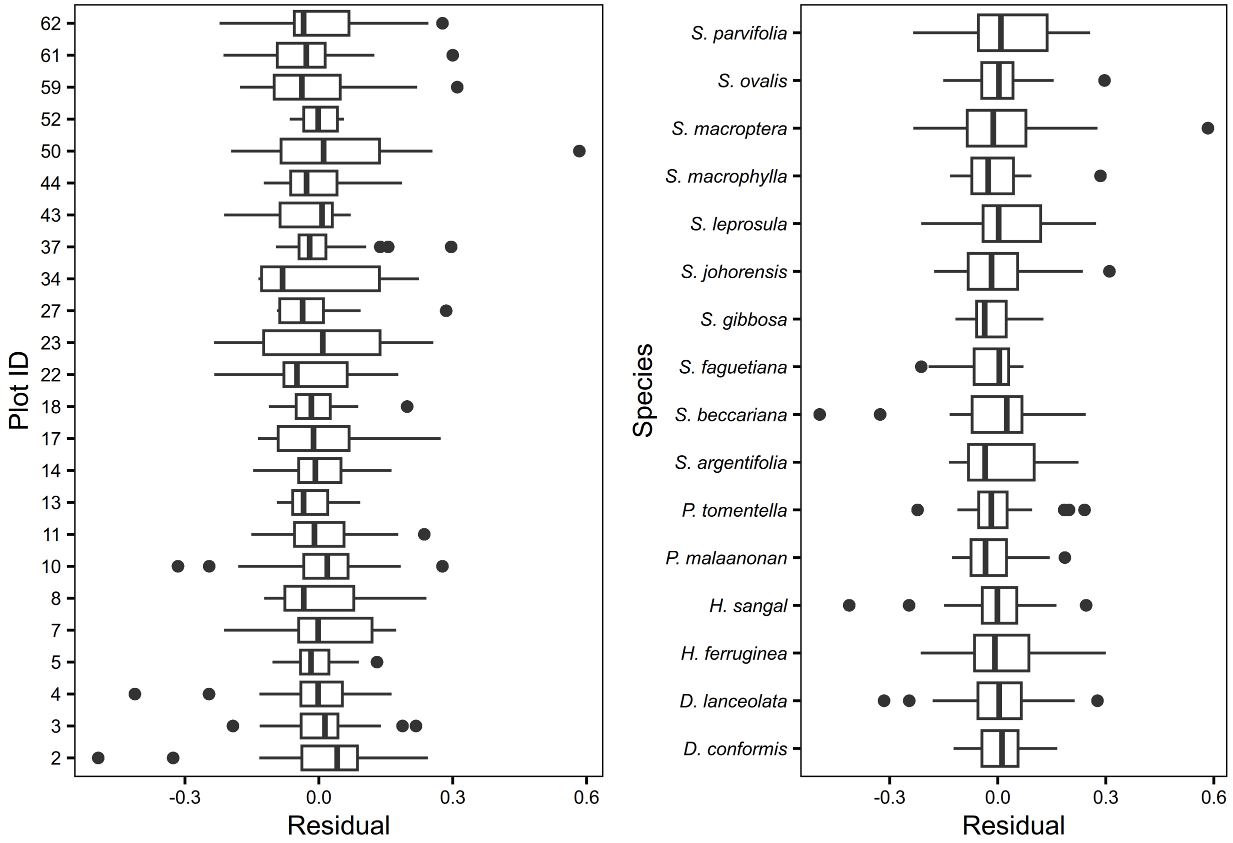


```r
### Save for later
fig_s5b <- p2
```

We combine the species estimates from the survival and growth models:

```r
### Construct final figure
fig_s5 <- fig_s5a + fig_s5b +
  plot_layout(ncol = 2, axes = "collect") +
  plot_annotation(tag_levels = "A") &
  theme(plot.tag = element_text(family = "Arial", face = "bold",
                                colour = "black", size = 10))

fig_s5
```

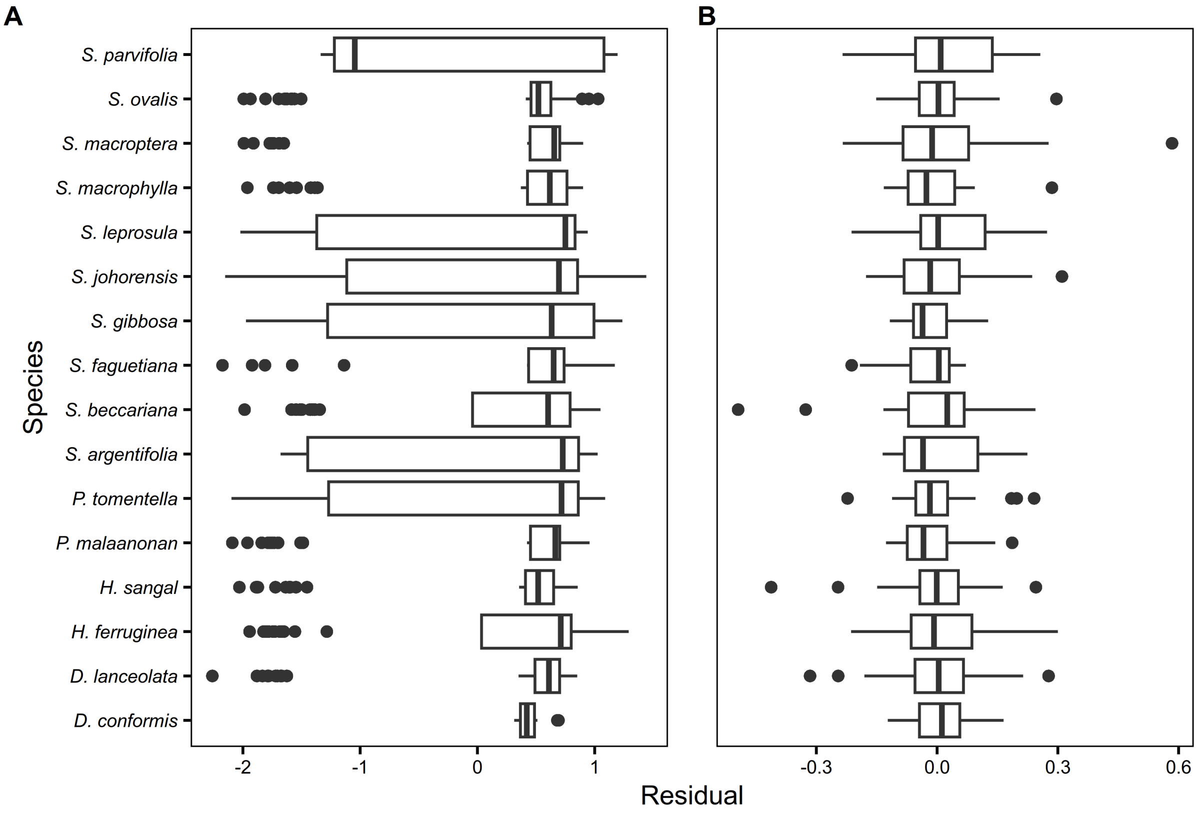


```
### Save
fig_s5 <- fig_s5 + theme_pdf
ggsave("Figures/Final/SI/fig_s5.pdf", fig_s5,
       width = 180, height = 150, units = "mm", dpi = 600, bg = "white", cairo_pdf)

ggsave("Figures/Final/SI/fig_s5.png", fig_s5,
       width = 180, height = 150, units = "mm", dpi = 600, bg = "white")
```

8 Results

8.1 Survival

Starting by looking at the results of our model (`survival_mod`).

8.1.1 Effect of cohort

Effect sizes and 95% confidence intervals. Cohort 1 had higher survival rates:

```
### Hold all other variables constant
sp_all <- expand.grid(openness_log         = mean(seedlings$openness_log),
                      ba_total_log_scaled = mean(seedlings$ba_total_log_scaled),
                      cohort               = factor(c("1", "2"),
                                                    levels = unique(seedlings$cohort)),
                      survival             = 0)

### Predicted values and bootstrapped CIs
sp_all$pred <- predict(survival_mod, newdata = sp_all, re.form = ~ 0, type = "response")
```

```r
bb <- bootMer(survival_mod,
              function(x) predict(x, newdata = sp_all, re.form = NA),
              nsim = 200)
bb$t <- plogis(bb$t)
bb_se <- apply(bb$t, 2, function(x) quantile(x, probs = c(.025, .975), na.rm = T))
sp_all$low <- bb_se[1,]
sp_all$upp <- bb_se[2,]

### Print
sp_all[, c("cohort", "pred", "low", "upp")]
```

```
##   cohort      pred       low       upp
## 1      1 0.8655991 0.8112049 0.9041439
## 2      2 0.7227225 0.6258919 0.7939025
```

8.1.2 Effect of canopy openness

Our predicted plots, holding the values of all other variables constant:

```r
### Hold all other variables constant
sp_all <- expand.grid(openness_log        = seq(min(seedlings$openness_log),
                                                 max(seedlings$openness_log),
                                                 length.out = 50),
                      ba_total_log_scaled = mean(seedlings$ba_total_log_scaled),
                      cohort              = factor(c("1", "2"),
                                                   levels = unique(seedlings$cohort)),
                      survival            = 0)

### Predicted values and bootstrapped CIs
sp_all$pred <- predict(survival_mod, newdata = sp_all, re.form = ~0, type = "response")
bb <- bootMer(survival_mod,
              function(x) predict(x, newdata = sp_all, re.form = NA),
              nsim = 200)
bb$t <- plogis(bb$t)
bb_se <- apply(bb$t, 2, function(x) quantile(x, probs = c(.025, .975), na.rm = T))
sp_all$low <- bb_se[1,]
sp_all$upp <- bb_se[2,]
```

First the plot for cohort 1 and save for later for figure A2:

```r
sp_cohort1 <- filter(sp_all, cohort == "1")
sp_cohort1_poly <- tibble(
  openness = c(exp(sp_cohort1$openness_log), rev(exp(sp_cohort1$openness_log))),
  pred_survival = c(sp_cohort1$low, rev(sp_cohort1$upp))
)

fig_s2a <- ggplot() +
  theme_doc +
  labs(y = "Proportion survival", x = "Canopy openness (%)") +
  scale_x_log10(breaks = c(2, 5, 10, 20, 40)) +
  geom_polygon(data = sp_cohort1_poly, aes(x = openness, y = pred_survival),
               fill = cols_2[2], colour = NULL, alpha = 0.5) +
  geom_line(data = sp_cohort1, aes(exp(openness_log), pred), lwd = 1,
            colour = cols_2[2]) +
  geom_point(data = seedlings, aes(exp(openness_log), survival),
```

```
              colour = cols_2[2], alpha = 0.15)
```

And then for cohort 2 (figure 4a):

```
sp_cohort2 <- filter(sp_all, cohort == "2")
sp_cohort2_poly <- tibble(
  openness = c(exp(sp_cohort2$openness_log), rev(exp(sp_cohort2$openness_log))),
  pred_survival = c(sp_cohort2$low, rev(sp_cohort2$upp))
)

fig_4a <- ggplot() +
  theme_doc +
  labs(y = "Proportion survival", x = "Canopy openness (%)") +
  scale_x_log10(breaks = c(2, 5, 10, 20, 40)) +
  geom_polygon(data = sp_cohort2_poly, aes(x = openness, y = pred_survival),
               fill = cols_2[2], colour = NULL, alpha = 0.5) +
  geom_line(data = sp_cohort2, aes(exp(openness_log), pred), lwd = 1,
            colour = cols_2[2]) +
  geom_point(data = filter(seedlings, cohort == "2"), aes(exp(openness_log), survival),
             colour = cols_2[2], alpha = 0.15)
```

Plot them side-by-side

```
(fig_s2a + labs(title = "Cohort 1")) + (fig_4a + labs(title = "Cohort 2")) +
  plot_layout(ncol = 2, axes = "collect")
```

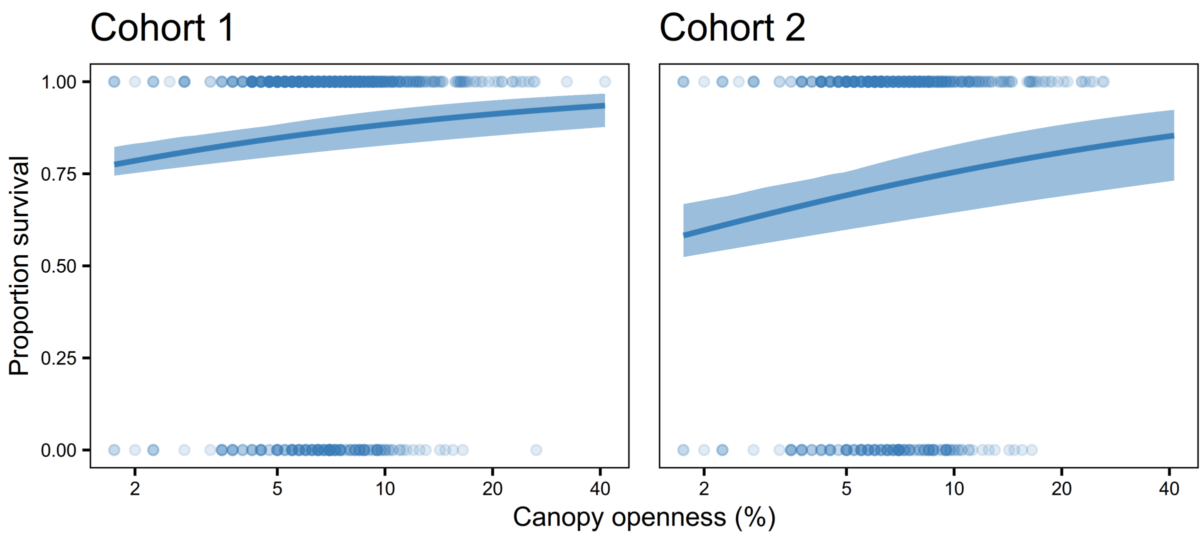

8.1.3 Effect of basal area

Our predicted plots, holding the values of all other variables constant:

```
### Hold all other variables constant
sp_all <- expand.grid(ba_total_log_scaled = seq(min(seedlings$ba_total_log_scaled),
                                                 max(seedlings$ba_total_log_scaled),
                                                 length.out = 50),
                      openness_log        = mean(seedlings$openness_log),
                      cohort              = factor(c("1", "2"),
                                                   levels = unique(seedlings$cohort)),
```

```r
                      survival            = 0)

### Predicted values and bootstrapped CIs
sp_all$pred <- predict(survival_mod, newdata = sp_all, re.form = ~0, type = "response")
sp_all$ba_total_log <- sp_all$ba_total_log_scaled + mean(seedlings$ba_total_log)
bb <- bootMer(survival_mod,
              function(x) predict(x, newdata = sp_all, re.form = NA),
              nsim = 200)
bb$t <- plogis(bb$t)
bb_se <- apply(bb$t, 2, function(x) quantile(x, probs = c(.025, .975), na.rm = T))
sp_all$low <- bb_se[1,]
sp_all$upp <- bb_se[2,]
```

First the plot for cohort 1 and save for later for figure A4:

```r
sp_cohort1 <- filter(sp_all, cohort == "1")
sp_cohort1_poly <- tibble(
  ba_total = c(exp(sp_cohort1$ba_total_log), rev(exp(sp_cohort1$ba_total_log))),
  pred_survival = c(sp_cohort1$low, rev(sp_cohort1$upp))
)

fig_s4 <- ggplot() +
  theme_doc +
  labs(y = "Proportion survival", x = expression(paste("Total basal area ","(", m^2,")"))) +
  scale_x_log10(breaks = c(0.05, 0.14, 0.35, 1, 2.7)) +
  geom_polygon(data = sp_cohort1_poly, aes(x = ba_total, y = pred_survival),
               fill = cols_2[2], colour = NULL, alpha = 0.5) +
  geom_line(data = sp_cohort1, aes(exp(ba_total_log), pred), lwd = 1,
            colour = cols_2[2]) +
  geom_point(data = seedlings, aes(exp(ba_total_log), survival),
             colour = cols_2[2], alpha = 0.15)

fig_s4
```

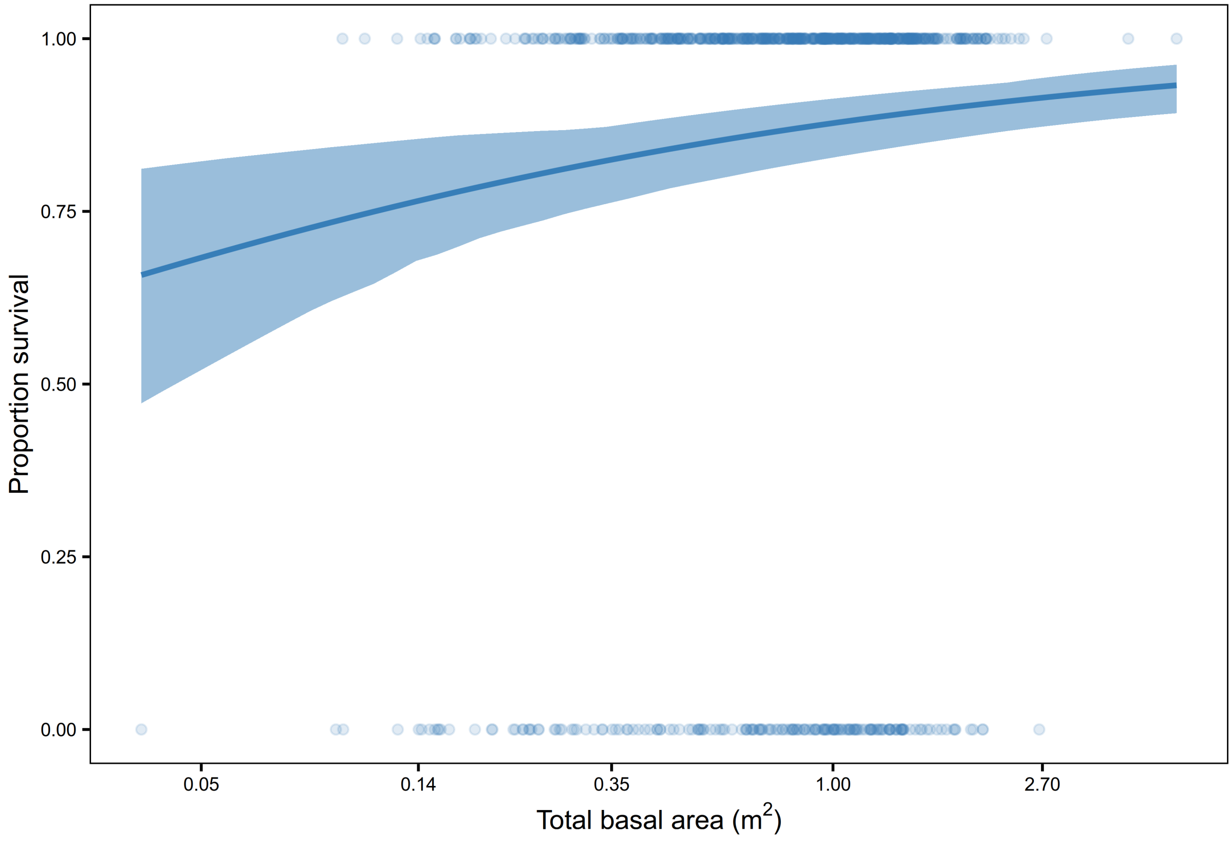


```r
### Save
fig_s4 <- fig_s4 + theme_pdf
ggsave("Figures/Final/SI/fig_s4.pdf", fig_s4,
       width = 180, height = 100, units = "mm", dpi = 600, bg = "white", cairo_pdf)

ggsave("Figures/Final/SI/fig_s4.png", fig_s4,
       width = 180, height = 100, units = "mm", dpi = 600, bg = "white")
```

And then for cohort 2 (figure 5):

```r
sp_cohort2 <- filter(sp_all, cohort == "2")
sp_cohort2_poly <- tibble(
  ba_total = c(exp(sp_cohort2$ba_total_log), rev(exp(sp_cohort2$ba_total_log))),
  pred_survival = c(sp_cohort2$low, rev(sp_cohort2$upp))
)

fig_5 <- ggplot() +
  theme_doc +
  labs(y = "Proportion survival", x = expression(paste("Total basal area ","(", m^2,")"))) +
  scale_x_log10(breaks = c(0.05, 0.14, 0.35, 1, 2.7)) +
  geom_polygon(data = sp_cohort1_poly, aes(x = ba_total, y = pred_survival),
               fill = cols_2[2], colour = NULL, alpha = 0.5) +
  geom_line(data = sp_cohort1, aes(exp(ba_total_log), pred), lwd = 1,
            colour = cols_2[2]) +
  geom_point(data = seedlings, aes(exp(ba_total_log), survival),
             colour = cols_2[2], alpha = 0.15)
```

```
fig_5
```

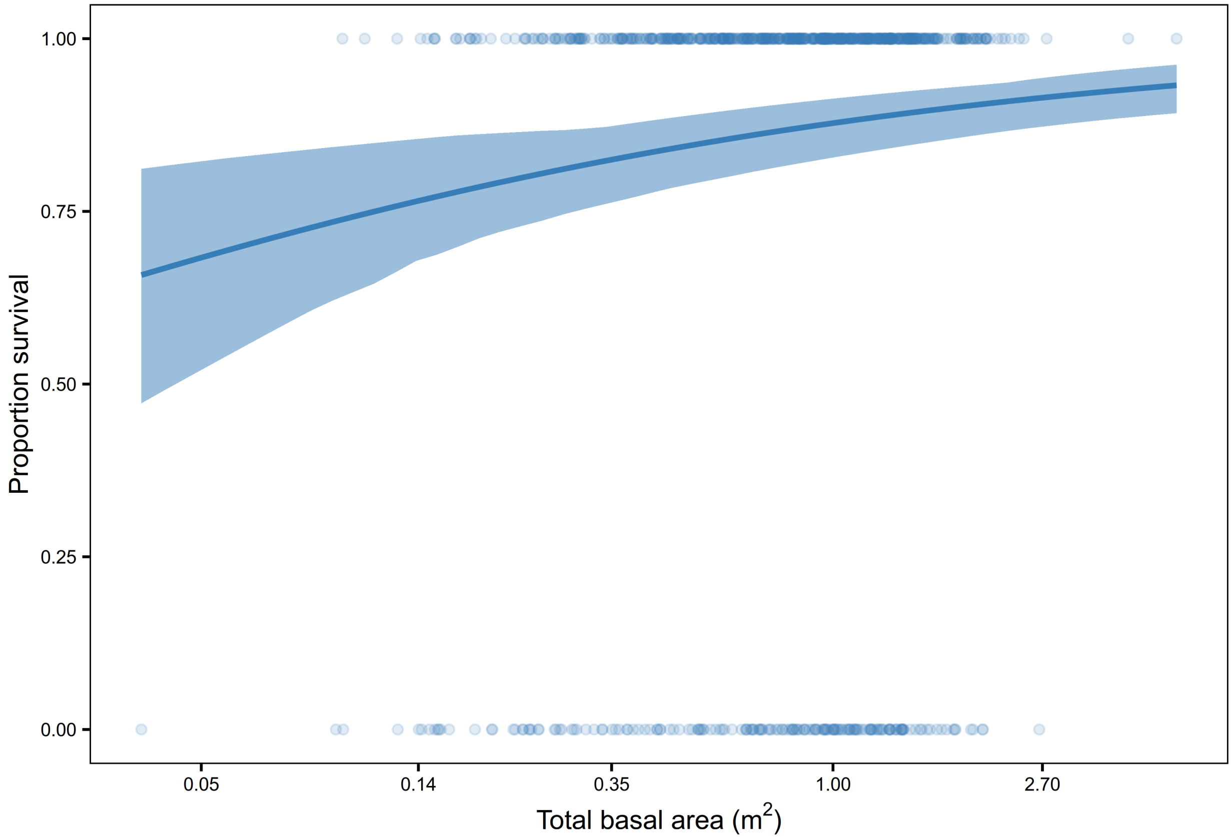


```
### Save
fig_5 <- fig_5 + theme_pdf
ggsave("Figures/Final/fig_5.pdf", fig_5,
       width = 180, height = 100, units = "mm", dpi = 600, bg = "white", cairo_pdf)

ggsave("Figures/Final/fig_5.png", fig_5,
       width = 180, height = 100, units = "mm", dpi = 600, bg = "white")
```

8.2 Growth

`growth_mod` is a simpler model than `survival_mod`, with only canopy type and canopy openness being retained.

8.2.1 Effect of cohort

```
### Hold all other variables constant
sp_all <- tibble(openness_log = mean(growth$openness_log),
                 cohort = factor(c("1", "2"),
                                 levels = unique(growth$cohort)),
                 rgr = 0)

### Predictions and CIs
sp_all$pred <- predict(growth_mod, newdata = sp_all, re.form = ~0, type = "response")
bb <- bootMer(growth_mod,
```

```
              function(x) predict(x, newdata = sp_all, re.form = NA),
              nsim = 200)
bb_se <- apply(bb$t, 2, function(x) quantile(x, probs = c(.025, .975), na.rm = T))
sp_all$low <- bb_se[1,]
sp_all$upp <- bb_se[2,]
```

```
sp_all[, c("cohort", "pred", "low", "upp")]
```

```
## # A tibble: 2 x 4
##   cohort   pred    low    upp
##   <fct>   <dbl>  <dbl>  <dbl>
## 1 1      0.0756 0.0516 0.0971
## 2 2      0.117  0.0982 0.135
```

8.2.2 Effect of canopy openness

Our predicted plots, holding the values of all other variables constant:

```
### Hold all other variables constant
sp_all <- expand.grid(openness_log        = seq(min(growth$openness_log),
                                                max(growth$openness_log),
                                                length.out = 50),
                      ba_total_log_scaled = mean(growth$ba_total_log_scaled),
                      cohort              = factor(c("1", "2"),
                                                   levels = unique(growth$cohort)),
                      survival            = 0)

### Predicted values and bootstrapped CIs
sp_all$pred <- predict(growth_mod, newdata = sp_all, re.form = ~0, type = "response")
bb <- bootMer(growth_mod,
              function(x) predict(x, newdata = sp_all, re.form = NA),
              parallel = "snow", nsim = 200)
bb_se <- apply(bb$t, 2, function(x) quantile(x, probs = c(.025, .975)))
sp_all$low <- bb_se[1,]
sp_all$upp <- bb_se[2,]
```

First the plot for cohort 1 and save for later for figure A2b:

```
sp_cohort1 <- filter(sp_all, cohort == "1")
sp_cohort1_poly <- tibble(
  openness = c(exp(sp_cohort1$openness_log), rev(exp(sp_cohort1$openness_log))),
  pred_rgr = c(sp_cohort1$low, rev(sp_cohort1$upp))
)

fig_s2b <- ggplot() +
  theme_doc +
  labs(y = expression(paste("Relative growth rate (mm ",mm^-1, year^-1 ,")")),
       x = "Canopy openness (%)") +
  scale_x_log10(breaks = c(2, 5, 10, 20, 40)) +
  geom_polygon(data = sp_cohort1_poly, aes(x = openness, y = pred_rgr),
               fill = cols_2[1], colour = NULL, alpha = 0.5) +
  geom_line(data = sp_cohort1, aes(exp(openness_log), pred), lwd = 1,
            colour = cols_2[1]) +
  geom_point(data = growth, aes(exp(openness_log), rgr),
             colour = cols_2[1], alpha = 0.15)
```

Repeat for cohort 2 (Fig. 4b):

```
sp_cohort2 <- filter(sp_all, cohort == "2")
sp_cohort2_poly <- tibble(
  openness = c(exp(sp_cohort2$openness_log), rev(exp(sp_cohort2$openness_log))),
  pred_rgr = c(sp_cohort2$low, rev(sp_cohort2$upp))
)

fig_4b <- ggplot() +
  theme_doc +
  labs(y = expression(paste("Relative growth rate (mm ",mm^-1, year^-1 ,")")),
       x = "Canopy openness (%)") +
  scale_x_log10(breaks = c(2, 5, 10, 20, 40)) +
  geom_polygon(data = sp_cohort2_poly, aes(x = openness, y = pred_rgr),
               fill = cols_2[1], colour = NULL, alpha = 0.5) +
  geom_line(data = sp_cohort2, aes(exp(openness_log), pred), lwd = 1,
            colour = cols_2[1]) +
  geom_point(data = growth, aes(exp(openness_log), rgr),
             colour = cols_2[1], alpha = 0.15)
```

Look at cohorts 1 and 2 side-by-side:

```
(fig_s2b + labs(title = "Cohort 1")) + (fig_4b + labs(title = "Cohort 2")) +
  plot_layout(ncol = 2, axes = "collect")
```

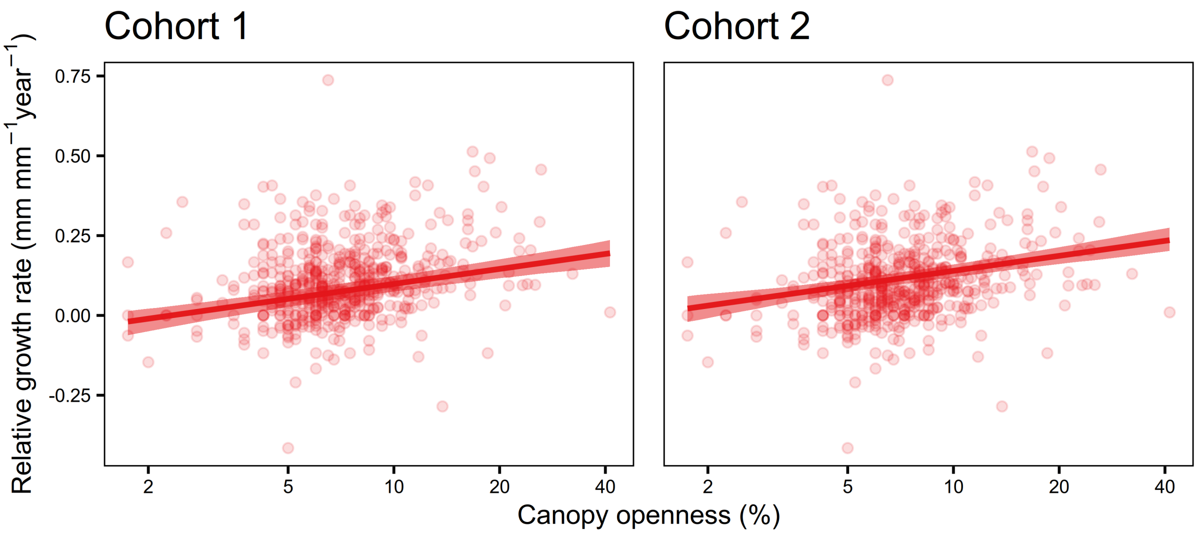

Construct final figure 4:

```
fig_4 <- (fig_4a + theme_pdf) + (fig_4b + theme_pdf) +
  plot_layout(ncol = 2) +
  plot_annotation(tag_level = "A") &
  theme(plot.tag = element_text(family = "Arial", colour = "black",
                                face = "bold", size = 10))

### Save
ggsave("Figures/Final/fig_4.pdf", fig_4,
       width = 180, height = 70, units = "mm", dpi = 600, bg = "white", cairo_pdf)
```

```r
ggsave("Figures/Final/fig_4.png", fig_4,
       width = 180, height = 70, units = "mm", dpi = 600, bg = "white")
```

Construct final figure A2:

```r
fig_s2 <- (fig_s2a + theme_pdf) + (fig_s2b + theme_pdf) +
  plot_layout(ncol = 2) +
  plot_annotation(tag_level = "A") &
  theme(plot.tag = element_text(family = "Arial", colour = "black",
                                face = "bold", size = 10))

### Save
ggsave("Figures/Final/SI/fig_s2.pdf", fig_s2,
       width = 180, height = 70, units = "mm", dpi = 600, bg = "white", cairo_pdf)

ggsave("Figures/Final/SI/fig_s2.png", fig_s2,
       width = 180, height = 70, units = "mm", dpi = 600, bg = "white")
```

9 Session information

First we tidy up:

```r
rm(list = ls())
gc()
```

```
##            used  (Mb) gc trigger  (Mb) max used  (Mb)
## Ncells 3443925 184.0    5941338 317.4  5941338 317.4
## Vcells 6467552  49.4   14787234 112.9 14787174 112.9
```

And print session info:

```r
sessionInfo()
```

```
## R version 4.5.2 (2025-10-31)
## Platform: x86_64-pc-linux-gnu
## Running under: Linux Mint 22.2
##
## Matrix products: default
## BLAS:   /usr/lib/x86_64-linux-gnu/blas/libblas.so.3.12.0
## LAPACK: /usr/lib/x86_64-linux-gnu/lapack/liblapack.so.3.12.0  LAPACK version 3.12.0
##
## locale:
##  [1] LC_CTYPE=en_SG.UTF-8       LC_NUMERIC=C
##  [3] LC_TIME=en_SG.UTF-8        LC_COLLATE=en_SG.UTF-8
##  [5] LC_MONETARY=en_SG.UTF-8    LC_MESSAGES=en_SG.UTF-8
##  [7] LC_PAPER=en_SG.UTF-8       LC_NAME=C
##  [9] LC_ADDRESS=C               LC_TELEPHONE=C
## [11] LC_MEASUREMENT=en_SG.UTF-8 LC_IDENTIFICATION=C
##
## time zone: Asia/Singapore
## tzcode source: system (glibc)
##
## attached base packages:
## [1] stats     graphics  grDevices utils     datasets  methods   base
##
```

```
## other attached packages:
##  [1] faraway_1.0.9      arm_1.14-4         MuMIn_1.48.11
##  [4] blmeco_1.4         MASS_7.3-65        lattice_0.22-7
##  [7] lme4_1.1-37        Matrix_1.7-4       plotrix_3.8-4
## [10] patchwork_1.3.2    ggplot2_4.0.0      gridExtra_2.3
## [13] RColorBrewer_1.1-3 sf_1.0-21          broom.mixed_0.2.9.6
## [16] forcats_1.0.1      stringr_1.5.2      tibble_3.3.0
## [19] tidyr_1.3.1        dplyr_1.1.4
##
## loaded via a namespace (and not attached):
##  [1] tidyselect_1.2.1   vipor_0.4.7        farver_2.1.2       S7_0.2.0
##  [5] fastmap_1.2.0      digest_0.6.37      lifecycle_1.0.4    magrittr_2.0.4
##  [9] compiler_4.5.2     rlang_1.1.6        tools_4.5.2        utf8_1.2.6
## [13] yaml_2.3.10        knitr_1.50         labeling_0.4.3     classInt_0.4-11
## [17] abind_1.4-8        KernSmooth_2.23-26 withr_3.0.2        purrr_1.1.0
## [21] grid_4.5.2         stats4_4.5.2       e1071_1.7-16       future_1.67.0
## [25] globals_0.18.0     scales_1.4.0       dichromat_2.0-0.1  cli_3.6.5
## [29] rmarkdown_2.30     ragg_1.5.0         reformulas_0.4.2   generics_0.1.4
## [33] rstudioapi_0.17.1  minqa_1.2.8        DBI_1.2.3          ggbeeswarm_0.7.3
## [37] proxy_0.4-27       splines_4.5.2      parallel_4.5.2     s2_1.1.9
## [41] vctrs_0.6.5        boot_1.3-32        beeswarm_0.4.0     listenv_0.10.0
## [45] systemfonts_1.3.1  units_1.0-0        glue_1.8.0         parallelly_1.45.1
## [49] nloptr_2.2.1       codetools_0.2-20   stringi_1.8.7      gtable_0.3.6
## [53] furrr_0.3.1        pillar_1.11.1      htmltools_0.5.8.1  R6_2.6.1
## [57] textshaping_1.0.4  wk_0.9.4           Rdpack_2.6.4       evaluate_1.0.5
## [61] rbibutils_2.3      backports_1.5.0    broom_1.0.10       class_7.3-23
## [65] Rcpp_1.1.0         coda_0.19-4.1      nlme_3.1-168       mgcv_1.9-3
## [69] xfun_0.54          pkgconfig_2.0.3
```
